# Electrodialysis Applications in Wastewater Treatment for Environmental Protection and Resources Recovery: A Systematic Review on Progress and Perspectives

**DOI:** 10.3390/membranes10070146

**Published:** 2020-07-09

**Authors:** Luigi Gurreri, Alessandro Tamburini, Andrea Cipollina, Giorgio Micale

**Affiliations:** Dipartimento di Ingegneria, Università degli Studi di Palermo, viale delle Scienze Ed. 6, 90128 Palermo, Italy; luigi.gurreri@unipa.it (L.G.); andrea.cipollina@unipa.it (A.C.); giorgiod.maria.micale@unipa.it (G.M.)

**Keywords:** electro-membrane process, electrodialysis reversal, bipolar membrane electrodialysis, selectrodialysis, electrodialysis metathesis, electrodeionisation, reverse electrodialysis, monovalent selective membranes, water reuse, brine valorisation

## Abstract

This paper presents a comprehensive review of studies on electrodialysis (ED) applications in wastewater treatment, outlining the current status and the future prospect. ED is a membrane process of separation under the action of an electric field, where ions are selectively transported across ion-exchange membranes. ED of both conventional or unconventional fashion has been tested to treat several waste or spent aqueous solutions, including effluents from various industrial processes, municipal wastewater or salt water treatment plants, and animal farms. Properties such as selectivity, high separation efficiency, and chemical-free treatment make ED methods adequate for desalination and other treatments with significant environmental benefits. ED technologies can be used in operations of concentration, dilution, desalination, regeneration, and valorisation to reclaim wastewater and recover water and/or other products, e.g., heavy metal ions, salts, acids/bases, nutrients, and organics, or electrical energy. Intense research activity has been directed towards developing enhanced or novel systems, showing that zero or minimal liquid discharge approaches can be techno-economically affordable and competitive. Despite few real plants having been installed, recent developments are opening new routes for the large-scale use of ED techniques in a plethora of treatment processes for wastewater.

## 1. Introduction

The growing water demand in urban, rural and industrial sites poses serious ecological and economic concerns in water management linked to resources depletion and wastes disposal. Several industrial processes use large water volumes, thus producing high quantities of wastewater or spent streams with contaminants and valuable components. In addition, municipal wastewater treatment plants effluents are not directly reusable.

Water recovery offers the possibility of sustainable development. On the other hand, it requires the design and implementation of advanced treatment methods, which represent a techno-economic challenge. In this framework, the zero liquid discharge (ZLD) concept aims at developing strategies to close the material loop, thus minimizing the liquid waste [1,2,3]. This approach is a specific accomplishment of circular economy [4,5], which proposes “business models based on reducing, alternatively reusing, recycling and recovering materials in production/distribution and consumption processes”, by replacing the old perspective of *end of life* [6].

Membrane processes are attracting a great deal of interest, and several studies have led to significant advances [7]. Among them, electro-membrane technologies separate ions by the selective transport through ion-exchange membranes (IEMs) under the influence of an electric field. A large variety of electro-membrane processes has been developed [8,9,10,11,12,13,14].

In particular, electrodialysis (ED) produces two streams with different concentrations flowing in alternate compartments separated alternatively by cation-exchange membranes (CEMs) and anion-exchange membranes (AEMs). ED may be cost-effective thanks to properties favourable to the attainment of large selectivity and product recovery, and to the avoided, or limited, need for chemicals [8,9,15,16,17]. At industrial scale, ED is mainly applied to desalinate brackish water for drinking water production. There have been installed also some ED plants to produce table salt from seawater desalination. However, many studies have focused on application of ED techniques in the (bio)chemistry, food processing, and pharmaceutical industries [8,9,10,11,13,17,18,19,20,21], encompassing wastewater treatment, recovery of chemicals or other valuable products, and removal of toxic components [8,9,13,17,20,22,23].

**Figure 1:**
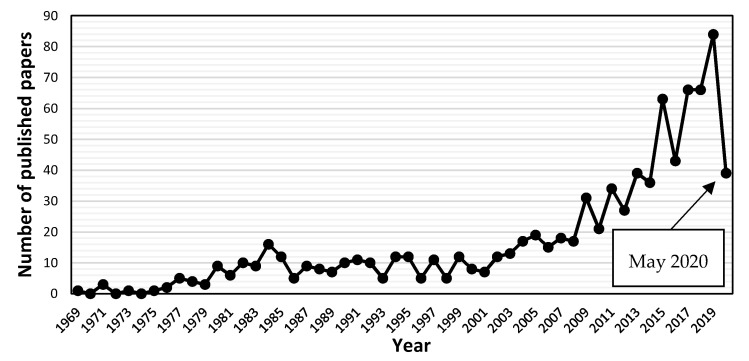
Scientific documents chronology reported on Scopus with “electrodialysis” and “wastewater” search words (article title, abstract or keywords). Source: www.scopus.com, accessed on 7 May 2020.

In regard to wastewater treatment via ED, the research exhibits an exponential growth in the last 20 years (Figure 1). More than 75% of the 879 scientific documents published since 1969 up until now fall in this period.

Many industrial effluents (e.g., from metal finishing, tanning, pulp and paper processing) have a complex composition with contaminants and/or valuable components, e.g., heavy metal ions, acids, organic matter, etc. Similarly, treated effluents from municipal or animal farming sources contain, for example, nutrients, as well as water. Finally, desalination plants’ reject brines may provide water and/or salt. Thanks to its ability in separating charged particles, ED methods can effectively recover water and/or other products from these effluents, including electrical energy.

Despite the fact that ED has a long history over more than 120 years, and that intense research activity has been developed, especially in recent years, only a few review articles have been published so far. In 2010, Strathmann [15] described the principle of ED, its operating conditions, and its design features, focusing on water desalination and recent advancements (profiled membranes). ED-related processes were included, by highlighting advantages and limitations. In 2018, our research group published a review paper on ED updated with the most recent developments for water desalination [16]. The main topics were IEMs progress and characterisation, hydrodynamics and transport phenomena, process models, other modelling tools, and ED-related technologies. Recently, Sajjad et al. [17] provided an overview on (waste)water treatment via ED, by discussing the main technological limitations.

The lack of an organic review on ED for wastewater treatment motivated the present work. For the first time, to the best of the authors’ knowledge, this paper presents a comprehensive and systematic review of studies on ED applications in wastewater treatment for environmental protection and recovery of resources, outlining the current status and the future prospect. The large variety of uses of conventional ED and similar technologies is discussed by analysing experimental results, process performance, strengths and drawbacks, and techno-economic competitiveness. Recent advances and emerging applications are reviewed, along with examples among the few well-established implementations in real environments.

## 2. Research Method, Rationale and Structure of the Review

The investigation of the review topic was based on a literature search in the Scopus electronic database. The search words listed in Table 1 were used without limits of date and by excluding only conference papers among the document types. This search found a total number of studies amounting approximatively to 1000, by excluding duplicate results. For the selection process, the eligibility criteria were relevance/consistency for/with the review topic, full text availability and accessibility, number of citations (except for most recent results), and journal metrics. The selected papers were organised in a bibliography on *Mendeley desktop*. The screening by full-text analysis filtered about 400 relevant higher-quality scientific papers to be reviewed and discussed (excluding those used for the fundamentals, Section 3).

**Figure 2:**
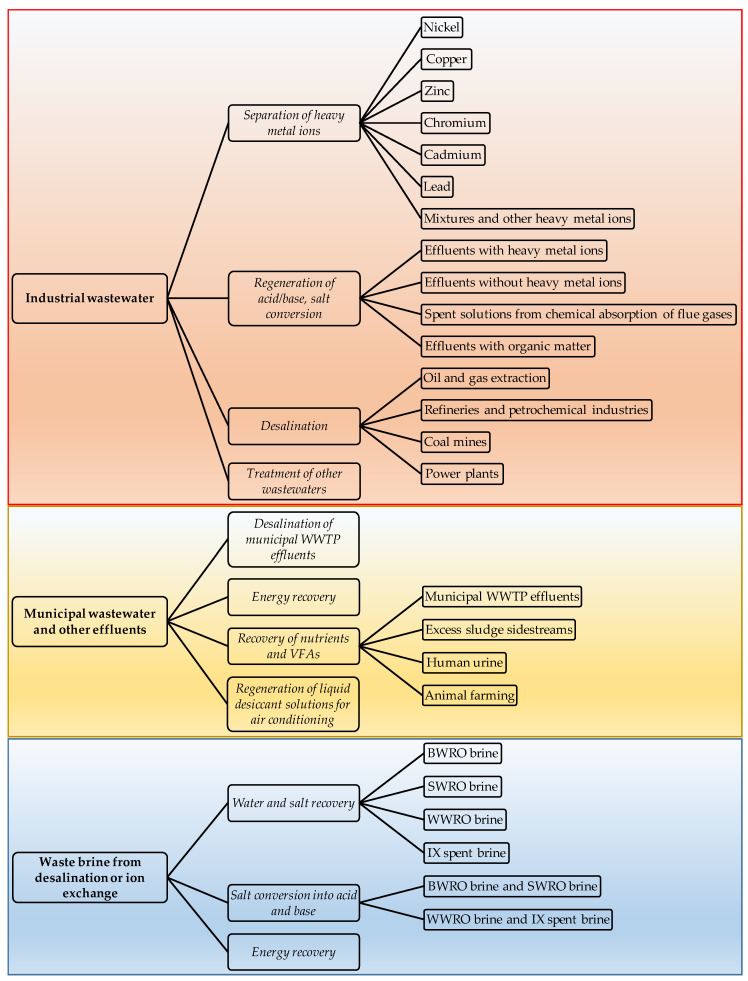
Classification of the selected papers, reflected in the review structure (Section 4, Section 5 and Section 6 and their sub-sections).

The selected articles were classified into three main categories according to the wastewater origin, and into sub-categories based on the treatment aim, as shown in Figure 2. A third level of sub-classification was used in some cases in order to distinguish among different waste effluents. Sections and sub-sections of the paper will reflect exactly this classification. Therefore, the paper is structured as follows. After a brief description of the process fundamentals (Section 3), Section 4, Section 5 and Section 6, which are the core of the review, correspond to the three main categories of the classification, and their sub-sections to the sub-categories. Where not specified, the data reported in the review will refer to lab-scale experiments. Otherwise, pilot plants and installations in real environments will be explicitly indicated throughout the paper. Finally, Section 7 provides discussion, conclusions and outlook, highlighting the main technical challenges, the current status of the process scale in the various applications, and the key points for future R&D.

## 3. Electrodialysis Process Fundamentals

### 3.1. Working Principle and Design/Operating Features of ED Processes

**Figure 3:**
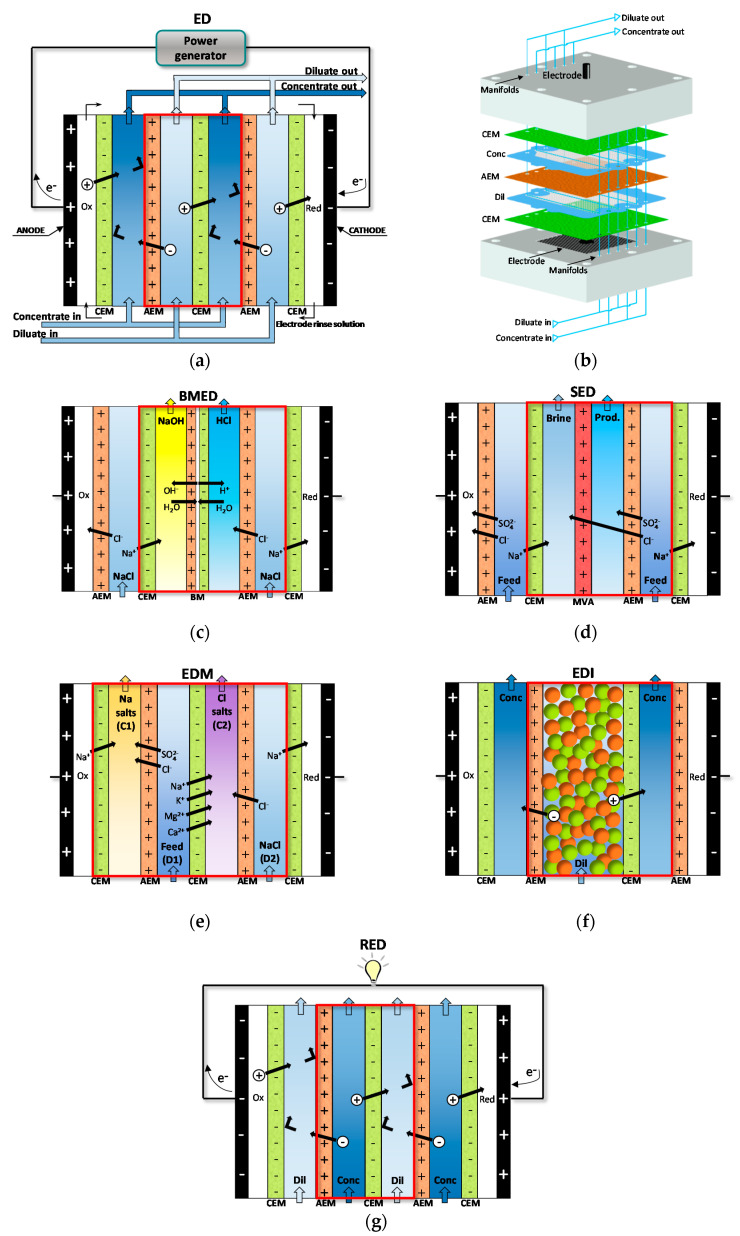
Schematics of ED techniques: (**a**) Conventional electrodialysis (ED); (**b**) Lab-scale ED stack with sheet-flow design (exploded view of one cell pair with an additional CEM); (**c**) Three-compartment bipolar membrane electrodialysis (BMED); (**d**) Selectrodialysis (SED) with MVA (the illustration refers to the fractionation of ${\mathrm{SO}}_{4}^{2-}$ from Cl^−^); (**e**) Electrodialysis metathesis (EDM); (**f**) Electrodeionisation (EDI) with ion-exchange resins filling the diluate; (**g**) Reverse electrodialysis (RED). Red rectangles indicate the repeating units. Panels (**a**,**b**,**g**) are reproduced (adapted) with permission from [16], published by Elsevier, 2018.

Figure 3a depicts a scheme of conventional ED. A pile of alternating AEMs and CEMs is arranged with alternating diluate and concentrate channels. At the extreme sides, the ED stack is completed by electrode compartments. Here, the external power supply establishes an electric potential difference, which causes redox reactions. A direct electric current flows through the external circuit as electronic current, and through the stack as ionic current, with cations and anions migrating towards the cathode and the anode, respectively. The co-ion block by the IEMs leads to a selective transport with a resulting salt concentration reduction/increase in the diluate/concentrate channels, respectively. The repetitive unit in a conventional stack, namely the “cell pair”, includes an AEM, a diluate, a CEM, and a concentrate. The two inlet feeds may differ each other.

The number of cell pairs in electrodialyzers ranges from few or some tens at bench- or pilot-scale up to hundreds in commercial stacks for real applications. The active area spans roughly from 0.01 to 1 m^2^ for the single membrane.

The plate-and-frame configuration is by far the most used. Net spacers (with typical thickness of ~300 μm to ~2 mm [16]) equipped with gaskets are placed between the IEMs in order to create the feed compartments (Figure 3b). The two main designs of the spacer-filled channels are sheet flow and tortuous path [15,16]. In the former pattern, the fluid flows roughly straight along a rectangular channel (Figure 3b). In the latter pattern, the feed moves along a serpentine. U-shaped channels with halfway features are almost common in large units, similarly to tortuous path layouts, while sheet flow channels are more used for small stacks. Membranes with built-in profiles avoid the use of net spacers [15,16], but they have been used only for theoretical or experimental studies [24,25,26,27,28].

The typical range of fluid velocity is 1–10 cm/s. However, along tortuous path layouts, the velocity may be increased to ~50 cm/s to counteract the poorer mixing. In most cases, flow regimes are steady, but turbulence may occur at higher velocities [16].

Batch operations with solution recirculation are typical for lab-scale studies, while continuous processes are basically limited to industrial plants. The “feed and bleed” operation (partial continuous recirculation) is commonly practiced to control water recovery and outlet concentrations [8]. Multi-stage schemes can be devised with several configurations (e.g., multiple hydraulic and/or electrical steps) in order to attain the wanted product features.

IEMs suffer from fouling phenomena less than semi-permeable membranes (e.g., for reverse osmosis). However, depending on the solutions treated, IEMs may experience serious deterioration, resulting in a higher electrical resistance and even in a physical damage. Both suspended and dissolved solids (organic and inorganic) can cause membrane fouling. Organic anions and inorganic compounds can often imply fouling of AEMs and CEMs, respectively [29]. Fouling caused by sparingly soluble salt precipitates is called scaling. Electrodialysis reversal (EDR) is commonly practiced for fouling mitigation. It is applied by periodic switching (cycles of minutes/hours) of electrode polarity (with simultaneous switch of feed solutions). As a result, charged components are removed from the IEM surface by migration in the contrary direction. In addition, feed pre-treatment and stack cleaning-in-place methods (acidic and/or alkaline solutions) can prevent and remove fouling, respectively.

Bipolar membrane electrodialysis (BMED) uses both monopolar membranes and bipolar membranes (BMs) to generate acid and base by water dissociation (Figure 3c). A BM consists of the overlapping of a cation-exchange layer (CEL) and an anion-exchange layer (AEL), whose inter-layer (thinner than 10 nm [8,30]) promotes water dissociation when a voltage (>0.83 V) is applied, thus releasing H^+^ and OH^−^ [8,10,12,30] at a rate that is six (or more) orders of magnitude larger than in solution [8,12]. This is caused by the catalytic role of the functional groups and/or of the catalyst in the bipolar region, and to the strong electric field (second Wien effect) [31]. The mechanisms of ion transfer and water dissociation are still under study via theoretical approaches and numerical models [31]. Novel preparation techniques based on electrospinning methods can produce high-performing BMs [32,33].

Figure 3c depicts the three-compartment BMED arrangement, which converts salt into acid and base. The repeating cell consists of: AEM, acid compartment, BM, base compartment, CEM, and diluate salt compartment. Protons and hydroxyl ions, generated in the bipolar region by the electric field, cross the CEL and the AEL and migrate to the acid and base channel, respectively, while salt anions and cations (e.g., Cl^−^ and Na^+^) in the salt channel cross the monopolar IEMs and migrate to the acid and base compartment, respectively. Nevertheless, other BMED arrangements have been developed with two-compartment repetitive units, with either BM-AEM or BM-CEM membranes for either acid or base production (and salt feed alkalisation or acidification). These configurations are used when it is possible or desired to obtain only one solution at high purity, with applications in regeneration processes [34,35,36,37,38]. Further BMED configurations include cell triplets with two monopolar IEMs of the same type. The outlet salt stream is sent to the acidic or alkaline channel to attain higher recovery rates [34,35].

Selective ED occurs within electrodialyzers containing monovalent selective membranes (MVMs), which may be anionic (MVAs) and/or cationic (MVCs) and segregate monovalent and multivalent ions. Specifically, the selectrodialysis (SED) process has a three-compartment configuration with an MVM and two conventional IEMs, and fractionates ions by using three different streams [39]. Figure 3d provides a sketch of SED fractionation of ${\mathrm{SO}}_{4}^{2-}$ from Cl^−^ contained in a feed solution. The results of the process are the mixture desalination, the product enrichment in divalent anions, and the brine concentration in monovalent ions.

ED stacks may be arranged to perform a metathesis of salts, known also as “double decomposition” (interchange of cations and anions between salts). With a couple of salts, one has:

*MX + M^′^X^′^ → M^′^X + MX^′^*. (1)

Electrodialysis metathesis (EDM) [40,41] has a four-compartment repeating unit which includes two diluate and two concentrate channels, all being different from each other, divided by two AEMs and two CEMs (Figure 3e). The feed (D1) contains a salt or a salts mixture, while a substitution solution flows along the other diluate channel (D2). From the D1 solution, anions move to the C1 concentrate, and cations move to the C2 concentrate; while from the D2 substitution solution, anions are transferred to the C2 concentrate, and cations are transferred to the C1 concentrate. As a result, the metathesis of salts between feed and substitution solution occurs in the concentrate products. In the example of Figure 3e, Na^+^ salts and Cl^−^ salts are generated inside compartments C1 and C2, respectively.

To boost the ED performance, ion-exchange resins (IXRs) can be inserted inside the channels. The hybridisation of ED and ion exchange (IX) is referred to as electrodeionisation (EDI) or continuous electrodeionisation (CEDI) [8,9,42,43] (Figure 3f). In EDI units, continuously regenerated IXRs beds within the diluate (sometimes also within the concentrate) cause a conductivity increment and a concentration polarisation reduction. Improved ion transport is obtained, thus making it possible to effectively treat very diluted solutions thanks to lower electrical resistances and higher limiting currents. The regeneration in situ of IXRs is carried out by H^+^ and/or OH^−^ from water dissociation occurring at bipolar contacts of IXRs particles or between IXRs and IEMs [42,43]. EDI is more suitable than its source technologies for producing industrial ultra-pure water [44] and for treating some kinds of wastewater, e.g., wastewater containing metal ions [42]. Moreover, fully regenerated IXRs can ionize and remove weakly ionized species (SiO_2_, CO_2_, boron, and NH_3_) [9,43]. The complex transport mechanism has been argued for several years through various models [42]. It involves the following steps [8,42,43]: ion diffusion through the solution (controlling step), IX, migration across the IXR bed and the IEM, and regeneration of the IXR.

EDI units are arranged with several configurations [8,9,42] by changing the IXRs bed composition and structure (mixed, separate or layered) and the IEMs number, placement and type, also including the employment of BMs as locations for water dissociation. EDI modules may be assembled with several repeating units between the electrodes, similarly to ED stacks. However, other arrangements with few compartments (three or some more) in total, including the electrode ones, were developed. Among them, some units exploit electrode water electrolysis to deliver H^+^ ad OH^−^ for the IXRs electro-regeneration [45,46,47,48,49,50], thus differing considerably from conventional ED. Limitations in EDI performance may derive from small current efficiencies in operations with high water dissociation [9], and from inhomogeneous flow distributions [42]. The former issue can be solved by identifying optimal values of the applied voltage, thus resulting in the co-existence of water dissociation and electroconvection in the overlimiting regime, which can enhance the process efficiency [51]. The latter issue can be addressed by adopting fixed resin wafers [52].

Reverse electrodialysis (RED) is the opposite process with respect to ED. RED produces electricity by converting the mixing free energy of two streams with different salt concentration (salinity gradient energy, or blue energy or osmotic energy), and is carried out with stacks equivalent to ED units [53,54,55,56,57] (Figure 3g). A high-salinity solution (concentrate, which is actually diluted along the channel) and a low-salinity solution (diluate, which is actually concentrated along the channel) flow through the two compartments of an RED cell pair. The most conventional solutions are seawater and river water, which would provide a maximum theoretical energy density of ~880 kJ/m^3^ (equal amounts of both solutions). However, recent studies have assessed the use of waste effluents.

The working principle of RED relies on the electrochemical equilibrium of the co-ion exclusion theorized by Donnan (see Section 3.2), which generates an electrical potential over IEMs immersed between two solutions at different concentration (i.e., different chemical potential). The sum of all membrane potentials of a stack is its electromotive force. It can be measured as the electric potential difference under open circuit conditions (open circuit voltage). When the circuit is closed with an external load, redox reactions at the electrode compartments convert the internal ions flux into the external electrons current. This implies that the voltage over the stack, which corresponds to the voltage over the external load, is reduced when the circuit is closed. Please note that in RED (generator), the cathode and anode are positive and negative, respectively, i.e., with the opposite charge with respect to ED (user). In addition to provide electricity, RED units may produce H_2_ via cathode reduction.

The power output depends on electromotive force and stack resistance. Therefore, a trade-off between them, maximizing the power supplied, is due to the effects of the diluate concentration. The RED performance may be significantly affected by the presence of divalent ions, which increase the membrane resistance and reduce the membrane permselectivity [58].

RED stacks are often operated with single pass (once-through), even in lab-scale experiments.

### 3.2. Ion Exchange Membranes and Mass Transfer

IEMs are dense membranes made by polymeric material with fixed charged groups and movable ions of opposite charge (counter-ions) [12]. IEMs allow counter-ions to pass, while blocking co-ions, which have the same sign of fixed charges. Cation-exchange membranes (CEMs) contain negative fixed groups such as SO_3_^−^, COO^−^, PO_3_^2−^, PO_3_H^−^, and C_6_H_4_O^−^; anion-exchange membranes (AEMs) contain positive fixed groups such as NH_3_^+^, NRH_2_^+^, NR_2_H^+^, NR_3_^+^, PR_3_^+^, and SR_2_^+^ [10]. As mentioned above, bipolar membranes (BMs) consist of an anion-exchange layer (AEL) overlapped to a cation-exchange layer (CEL). Monovalent selective membranes (MVMs) allow monovalent counter-ions to pass, whereas retain multivalent counter-ions [19,28,29,30]. IEM categories are distinguished on the basis of materials, functional groups, and microstructure [9]. For details on IEM features and methods of preparation, see [8,9,10,11,12,59,60].

The theorisation of co-ion exclusion by IEMs was introduced by Donnan equilibrium [8,61], which implies an electric double layer (EDL) at the membrane–solution boundary [8,9,59,61]. A membrane between two salt solutions with different concentration generates a voltage difference (Teorell-Meyer-Sievers) [9,59]:

(2) $$∆\varphi^{IEM}=\frac{RT}{F}\int^{}\sum^{}\frac{t_{i}^{IEM}}{z_{i}}d\ln a_{i},$$

where $∆\varphi^{IEM}$ is the “membrane potential”, R is the universal gas constant, T is the absolute temperature, F is Faraday’s constant, $z_{i}\;$ is the valence, $t_{i}^{IEM}$ is the transport number in the IEM, $a_{i}$ is the ion activity. Transport numbers are usually assumed constant [62], and, for a single salt, the following well-known expression is obtained [8,9,59,61]

(3) $$∆\varphi^{IEM}=\left(2\;t_{counter}^{IEM}-1\right)\frac{RT}{z_{i}F}ln\frac{a^{SOL,R}}{a^{SOL,L}},$$

where $a^{SOL,R}$ and $a^{SOL,L}$ are the salt activities at the right and left solution, respectively. Transport phenomena in IEMs and electrolyte solutions are often described through the Nernst–Planck equation, which encompasses the ion flux with three contributions (diffusion, migration and convection):

(4) $${\vec{J}}_{i}=-D_{i}\vec{\nabla }C_{i}-z_{i}FD_{i}C_{i}\vec{\nabla }\varphi +C_{i}\vec{u},$$

where *D_i_* is the ion diffusivity, *C_i_* is the ion concentration, φ is the electric potential and $\vec{u}$ is the velocity. For strong binary electrolytes, it can become [61]

(5) $${\vec{J}}_{i}=-D_{el}\vec{\nabla }C_{i}+\frac{t_{i}\vec{i}}{z_{i}F}+C_{i}\vec{u},$$

where $D_{el}$ is the electrolyte diffusivity, *t_i_* is the ion transport number and $\vec{i}$ is the current density. The Nernst–Planck formalism assumes negligible interactions among ions, thus being strictly valid for dilute solutions. Nevertheless, the Maxwell–Stefan and other rigorous, but more complex, approaches are less used [63,64,65].

From Faraday’s law, the electric current is

(6) $$\vec{i}=F\sum_{i}z_{i}{\vec{J}}_{i}.$$

The transport number of an ionic species is the relative portion of electric current that it carries [8,9,59,60]:

(7) $$t_{i}=\frac{z_{i}J_{i}}{\sum_{i}z_{i}J_{i}}.$$

The IEM permselectivity for a counter-ion is [8,11,60]:

(8) $$P=\frac{t_{i}^{IEM}-t_{i}^{SOL}}{1-t_{i}^{SOL}}.$$

The IEM permselectivity between two ions (A and B) is [9,59,60,66]:

(9) $$P_{B}^{A}=\frac{t_{A}^{IEM}/t_{B}^{IEM}}{N_{A}^{DIL-IEM}/N_{B}^{DIL-IEM}},$$

where $N^{DIL-IEM}$ is the equivalent concentration (of A or B) at the membrane–solution interface in the diluted side.

Several methods can evaluate permselectivity and transport numbers. Static techniques measure the membrane potential, while dynamic methods consist of electrodialysis experiments (current efficiency) [11,59,60] or chronopotentiometric measurements [11,60,67,68].

Electrical resistance is another important membrane property. It may be measured by either direct current [11,68,69,70] or alternating current [11,69,71,72,73,74,75] techniques. The last ones (electrochemical impedance spectroscopy) are more complex, but make it possible to separate EDL and polarisation contributions. At lower solution concentration, membrane resistance exhibits an increasing vertical asymptote [69,72,75]. This behaviour was associated with the IEM morphology by following the micro-heterogeneous model [75,76], which represents the membrane with multi-phase structure [77], also containing the solution from outside. There are various physical models, and also phenomenological models for membrane resistance [78]. Moreover, a diverse behaviour was found by some measurements [74], thus this topic could deserve further investigation.

Salt and water permeabilities are other IEM properties [9,59,79,80,81,82,83] that affect the ED efficiency. The diffusive fluxes of salt and water (from concentrate to diluate and vice versa, respectively) are driven by gradients of concentration and osmotic pressure, respectively. An additional water flux originates from electro-osmosis (water molecules in the salt ions solvation shell).

Concentration polarisation in ED is caused by the difference in the transport numbers between membrane and solution, so that a diffusive transport in solution maintains a constant overall flux [15,16,84]. In particular, salt depletion occurs at the IEM-diluted side, and salt enrichment takes place at the IEM-concentrated side. The Nernst model (film theory) can be used to study transport phenomena in IEM-solution systems [85,86]. As the electric current increases, the salt depletion is possible until reaching a null interface concentration, corresponding to the condition of diffusion-limited current. The theoretical diffusion-limited current density may be related to the mass transfer characteristics in the fluid channel, i.e., to the Sherwood number.

However, current–voltage curves exhibit three regions, including the manifestation of overlimiting currents [87,88,89,90,91,92]. The first region starts following a linear trend, but more and more pronounced polarisation effects (and larger Ohmic resistances in ED stacks) cause a reduced slope at higher currents. The second region is a low-slope transition step indicating the limiting current achievement (high resistance). In the third region, a secondary current growth occurs. When the change in slope is not evident, Cowan’s method (apparent resistance) can identify the limiting current [16,93,94].

The current that is carried by H^+^ and OH^−^ generated through water dissociation can partially explain the appearance of overlimiting currents [84,95,96,97,98,99]. Instead, other overlimiting mechanisms involve counter-ions by current-induced convection [67,88,91,100,101,102,103,104,105,106,107,108]. Electroconvection is the primary process that alters the depleted region for dilute solutions. An extended space charge region develops near the IEM, where the solution is not electroneutral, and inhomogeneous electric fields cause dynamic vortices [87,88,89,109,110,111,112,113,114,115,116]. The conductive and geometrical heterogeneity in the IEM surface [67,105,111,112,113,117,118,119,120,121] and other features, e.g., roughness, grade of hydrophobicity, and superficial charge density [102,103,122,123,124,125], affect overlimiting mechanisms and current–voltage curve. Another overlimiting mechanism is gravitational convection, which is caused by temperature or concentration gradients [96,111,114,119,126].

Overlimiting regimes may lead to an enhancement of mass transfer [127], and surface modifications can improve IEMs performance [102]. Therefore, ED operations at overlimiting conditions may be considered to enhance the process efficiency. Nevertheless, maintaining ED stacks below the limiting current is a traditional practice [8,103,128] for avoiding dangerous pH values posing risks of fouling and IEM damage [84,95,129,130].

Despite the actual limiting conditions depend significantly on the membrane properties, experimental correlations of the Sherwood number [63,128,131,132,133,134] or of the limiting current density [90,131,135,136,137] are given by similar expressions:

(10) Sh=aRebScc,

(11) $$i_{lim}=dC_{i}^{bulk}{}^{e}u^{b},$$

where Re and Sc are the Reynolds and Schmidt number, respectively, *u* is the solution velocity, and *a–e* are coefficients. Many experiments show *b* values around 0.5, but it can span in a broader range. Actually, power laws fit experimental data well in narrow Reynolds number ranges [128,135], while more complex trends develop at wider ranges (a similar behaviour is exhibited by the friction factor [138,139,140]). The coefficient *c* was found to be equal to 1/3 [141,142], but it can assume different values [139,143,144].

Please note that the current–voltage curve of a BM exhibits two limiting zones. At low currents, the first limit is due to salt ions transport. Then, water dissociation occurs. At high currents, the second limit is due to water transport, eventually implying membrane damage.

### 3.3. Performance Parameters

The performance of ED processes is governed by membrane selectivity and transport properties, non-Ohmic voltage drop given by the membrane potential (“back” electromotive force in most cases, electromotive force in RED), Ohmic voltage drop, and pumping power consumption. The voltage drop over the stack can be computed as [16,145,146]:

(12) $$V_{stack}=V_{ru}N_{ru}+Ir_{el}=\left(\pm r_{Ohm,\;ru}I+V_{non-Ohm,ru}\right)N_{ru}+Ir_{el},$$

where $V_{ru}$ is the voltage drop over a single repeating unit (e.g., cell pair or triplet), *N_ru_* is the number of repeating units, *I* is the electric current, *r_el_* is the resistance of the electrode compartments (negligible in stacks with many repetitive units), $r_{Ohm,ru}$ and $\;V_{non-Ohm,ru}$ are the Ohmic resistance and non-Ohmic voltage drop in the repeating unit, respectively, the sign “+” applies for ED methods using an electric current provided by an external power supply, i.e., all ED methods except for RED, where the sign “−” applies. The Ohmic resistance encompasses the contributions from all compartments and IEMs (counting also spacer shadow effects [71,147]). $V_{non-Ohm,ru}$ consists of membrane potentials, including polarisation effects. The electric power consumption (or production in RED) can be simply calculated by multiplying the stack voltage drop by the electric current. Actually, the overall power would include the pumping power [16] (it has to be added to the power consumption in most cases, and has to be subtracted to the power production only in RED). The specific energy consumption expresses the energy consumed per product unit volume (e.g., kWh/m^3^):

(13) $$E_{spec}=\frac{V_{stack}\;I\;}{Q_{prod}},\;\mathrm{for}\;\mathrm{continuous}\;\mathrm{operations}$$

(14) $$E_{spec}=\frac{\int_{0}^{\tau }V_{stack}\;I\;dt}{v_{prod}},\;\mathrm{for}\;\mathrm{batch}\;\mathrm{operations}$$

where *Q_prod_* is the product volume flow rate exiting the module, *t* is the time, *v_prod_* is the product volume at time *τ*. *E_spec_* can be expressed with reference to the transported mass of electrolyte (e.g., kWh/kg):

(15) $$E_{spec}=\frac{V_{stack}\;I\;}{\left|C_{el,prod,\;in}Q_{prod,\;in}-C_{el,prod}Q_{prod}\right|M_{el}},\;\mathrm{for}\;\mathrm{continuous}\;\mathrm{operations},$$

(16) $$E_{spec}=\frac{\int_{0}^{\tau }V_{stack}\;I\;dt}{\left|C_{el,prod,\;in}v_{prod,\;in}-C_{el,prod}v_{prod}\right|M_{el}},\;\mathrm{for}\;\mathrm{batch}\;\mathrm{operations},$$

where $C_{el,prod,\;in}$, $Q_{prod,\;in}$ and $v_{prod,\;in}$ are the inlet or initial product concentration, flow rate and volume, respectively, $C_{el,prod}$ is the concentration in the product outgoing from the stack or at time τ, and *M_el_* is the molar mass of electrolyte.

The current efficiency quantifies the utilization of the applied current by an ion species:

(17) $$\eta_{i}=\frac{z_{i}F\left|C_{i,prod,\;in}Q_{prod,\;in}-C_{i,prod}Q_{prod}\right|}{N_{ru}I},\;\mathrm{for}\;\mathrm{continuous}\;\mathrm{operations},$$

(18) $$\eta_{i}=\frac{z_{i}F\left|C_{i,prod,\;in}v_{prod,\;in}-C_{i,prod}v_{prod}\right|}{N_{ru}\int_{0}^{\tau }\;I\;dt},\;\mathrm{for}\;\mathrm{batch}\;\mathrm{operations}\;,$$

where $C_{i,prod,\;in}$ is the inlet or initial concentration of *i* in the product, $C_{i,prod}$ is the outlet or final (at time τ) concentration of *i* in the product. The total *η* for ion mixtures is the sum over all anions or cations. The current efficiency is less than 100% because of unwanted salt and water transport phenomena, water splitting, and current leakage (parasitic or shunt currents through manifolds [146]) [8]. Further performance parameters such as removal efficiency, concentration factor and water recovery, result from easy calculations.

In the special case of RED, an important performance parameter is the electromotive force, given by the open circuit voltage of the stack, which can be estimated as:

(19) $$V_{OC}=N_{ru}\left(\alpha_{CEM}+\alpha_{AEM}\right)\frac{RT}{zF}ln\frac{a^{CONC}}{a^{DIL}},$$

where $\alpha_{CEM}$ and $\alpha_{AEM}$ are the apparent permselectivity of CEM and AEM, respectively, and $a^{CONC}$ and $a^{DIL}$ are the salt activity in the concentrate and diluate, respectively. As *V_OC_* is usually estimated with the inlet concentrations, it may differ from the actual (local or average) non-Ohmic voltage drop used in Equation (12). When *V_OC_* is measured, the average permselectivity can be obtained from Equation (19).

The power density in RED is defined as the power divided by the total membrane area:

(20) $$P_{d}=\frac{V_{stack}I}{2N_{ru}A},$$

where *A* is the active area of one membrane. The voltage over the external load, and thus over the stack, depends on the external resistance (*V_stack_ = I·r_ext_*). By reducing the external resistance, the stack voltage decreases and the current increases up to short-circuit conditions, where the electromotive force is completely consumed inside the stack. The power *V_stack_·I* theoretically follows a parabolic trend as a function of *I* or *V_stack_*, exhibiting a maximum under conditions halfway between open-circuit and short-circuit, in which the external resistance is equal to the stack resistance. The theoretical maximum power density is given by:

(21) $$P_{d,max}=\frac{E_{OCV}^{2}}{8r_{stack}N_{ru}A},$$

where *r_stack_* is the stack resistance (including cell pairs and electrode chambers). With the conventional couple seawater-river water at ambient temperature, *P_d,max_* is in the order of ~1 W/m^2^, with the highest measured value being 2.4 W/m^2^ [54,148]. As the power density produced by RED units is modest, the pumping power consumption cannot be neglected. Therefore, the net power *P_d,net_* (and, more specifically, the net power corresponding to the maximum gross power, *P_d,max,net_*) is an important performance parameter [149]. As a function of the fluid velocity, the net power (both *P_d,net_* and *P_d,max,net_*) first exhibits an increasing trend, then reaches a maximum, and finally decreases. Similarly to Equation (13), the specific energy production, or energy density, may be calculated for RED operations.

## 4. Industrial Wastewater

Waste effluents from industry may have different compositions. However, they often contain dissolved ions. Electrodialytic treatments for industrial wastewater can be classified in: separation of heavy metal ions (Section 4.1); regeneration of acid/base, salt conversion (Section 4.2); desalination (Section 4.3). The applications studied for the main types of industrial wastewater are examined first. Then, further studies on other waste effluents are collected in Section 4.4.

### 4.1. Separation of Heavy Metal Ions

Heavy metals are harmful pollutants characterized by toxicity, carcinogenicity, non-biodegradability, and persistence in the environment and in living beings. Among the treatment processes proposed for wastewater containing heavy metal ions [150], electrodialytic methods have been tested for industrial effluents from several processes (metal finishing, leather industry, etc.) aiming at reuse. For instance, ED can recuperate water and metals from spent baths or rinse waters of plating processes [151], and different EDI configurations offer several alternatives to ED [42,152].

Several studies have focussed on transport phenomena by assessing IEMs properties, pH effect, complexes formation and ions competition, and by developing modified or novel IEMs. Among them, experiments with aqueous solutions of Ni [153,154,155,156,157], Cu [158,159,160,161,162], Zn [157,163,164], Cr [154,156,165,166,167,168,169,170], Fe [154,156] and Pb [155,157] have been conducted, as well as with mixtures (e.g., Ni, Cu and Pb, or Cu and Zn) [171,172], thus providing important insights on basic phenomena and ED processes. Another crucial aspect is the identification of optimal operative conditions. With this aim, Taguchi’s method was adopted for experiments with metal ions present as single salts or salt mixtures [173,174]. It is a powerful method of design of experiments with optimisation of control parameters, and is based on orthogonal arrays that reduce the number of tests. The statistical analysis of the experimental results was performed by analysis of variance, evaluating error variance and relative importance of the various factors. Moreover, validated models can be adopted for sensitivity analyses and optimisation studies [173]. 

Each of the next sections, from Section 4.1.1, Section 4.1.2, Section 4.1.3, Section 4.1.4, Section 4.1.5 and Section 4.1.6 focuses on main wastewaters with a single heavy metal ion. Finally, Section 4.1.7 focuses on mixtures of metal ions and waste effluents with other metal ions.

#### 4.1.1. Nickel

**Figure 4:**
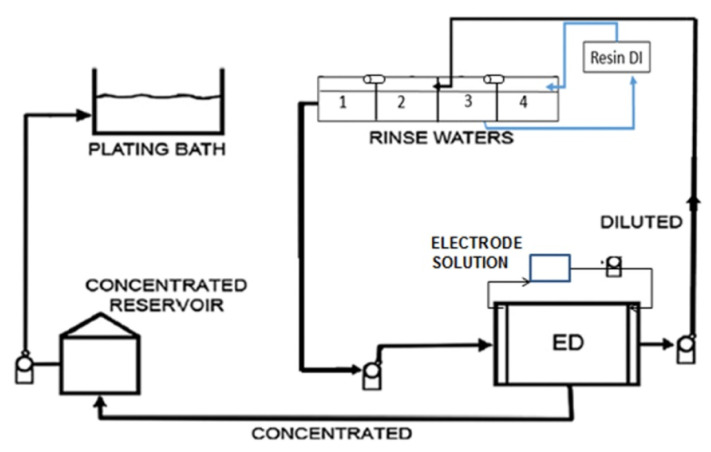
Flow-chart of the ED treatment for Ni electroplating rinse wastewater (the IXR extended the water cycle within the third and fourth tank). Reproduced with permission from [177], published by Elsevier, 2017.

Nickel is used for the plating processes of metal pieces with a galvanic bath, which is followed by multiple rinse stages with water. ED can be used to treat the first rinse solution, thus recovering a Ni^2+^ concentrate recycled to the galvanic bath, and a diluate recycled to the rinse stages (Figure 4). In an early study with pilot ED testing, Ni was recovered by 90% (5 g/L rinse wastewater) [175].

Recent experiments with artificial solutions of electroplating Watts’ bath (65 g/L NiCl_2_, 275 g/L NiSO_4_, 45g/L H_3_BO_3_, organic additives) recovered ~95–99% of ions and exhibited an acceptable quality of plated pieces [176]. A scale-up was then performed [177], by operating an ED plant introduced within an industrial plating process for 30 days (Figure 4), demonstrating techno-economic feasibility (saving of 3800 US$/y with *E_spec_* of 2.8 kWh/m^3^ for treating 480 L/day).

An interesting alternative for ED units with enhanced current efficiencies is given by corrugated membranes [178]. ED was also tested with electroless plating spent solutions, removing harmful ions (${\mathrm{HPO}}_{3}^{2-}$, ${\mathrm{SO}}_{4}^{2-}$, and Na^+^), and maintaining useful ions (Ni^2+^, $H_{2}{\mathrm{PO}}_{2}^{-}$, and organic acids) at high concentration [179].

An electrolysis-ED-EDI combined system (13-cell-pair EDI equipped with mixed IXRs in all channels) yielded ~99.8% of Ni^2+^ recovery with 93.9% of purity from a synthetic solution [180]. A simpler two-stage EDI was developed to mitigate the back diffusion [181]. By using a model Ni electroplating rinse solution at 50 mg/L, the first stack diluate effluent (~3 mg/L) was the initial feed of both compartments of the second stack. Mixed beds in the concentrate channels minimized the metal hydroxide precipitation in the 1st stage by limiting the contact probability of OH^−^ with Ni^2+^ (the lower concentration did not require this measure in the 2nd stage concentrate). Concentration and enhanced purification were accomplished. Ni^2+^ was separated by over 99.8% with *E_spec_* of 0.64 kWh/m^3^, and the solutions produced were suitable for use in plating and rinsing operations. An economic analysis prospected significant savings compared to chemical precipitation.

Numerous EDI configurations deviating from ED stacks have been tested. They include three-compartment electro-regenerated devices [45,49,50,182], among which there are a hybrid system coupling EDI with capacitive deionisation [183], and a unit without membranes and with electrostatic shielding regions made of graphite powder filling the concentrate compartments [184].

#### 4.1.2. Copper

The ED efficacy in Cu^2+^ separation has been proved. For example, removal percentages of ~97% were attained under optimal conditions [185]. ED can treat and recycle rinsing water from electroless plating [186], and baths and rinse solutions from cyanide electroplating (flowing through concentrate and diluate compartments, respectively) [187].

Non-toxic cyanide-free electroplating baths can be reclaimed by ED. Model rinse waters with 683–1281 mg/L 1-hydroxyethane 1,1-diphosphonic acid and 32–48 mg/L Cu^2+^ (from Cu alkaline strike bath) were used [188]. Despite various ionic complexes formed as the pH was changed, diluate and concentrate products were suitable for recycle in the bath and rinsing processes, respectively (maximum recovery of 99.7% for Cu, 94.4% for organic acid). Further experiments again achieved high recoveries, and pieces of electroplated Zamak alloy exhibited good-quality coatings [189]. However, AEMs properties were worsened (electrical resistance and limiting current), likely because of interactions between organic acids or their chelates and fixed groups. Transport properties were restored only in part by cleaning procedures.

Overlimiting regimes were shown to lead to high values of separation percentage and *η* [159]. However, copper hydroxide may precipitate causing scaling [190].

**Figure 5:**
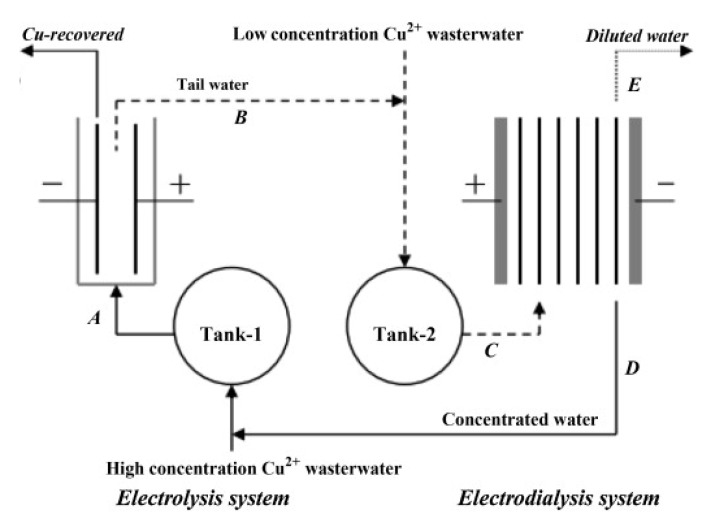
Electrolysis-ED integrated system treating CuSO_4_ solutions. Reproduced with permission from [191], published by Elsevier, 2011.

An integrated electrochemical process was developed by a pilot system with ED and electrolysis (Figure 5), recovering over 99% of Cu^2+^ and all the water from synthetic solutions (165–504 mg/L), with *E_spec_* of ~2 kWh/m^3^ in the ED stage [191].

Further combined systems aim at treating multiple wastewaters. For example, a process integrating microbial desalination cell, precipitation and ED was developed to treat simultaneously domestic wastewater, Cu wastewater and salt water [192]. In particular, ED finalized the removal of Cu residues and the desalination.

Metal–organic complexes can be separated by ED, which, for example, showed higher efficiencies with Cu–EDTA complexes compared to other electrochemical technologies [193].

EDI processes are suitable for Cu diluted wastewaters, such as plating rinse solutions, as shown, e.g., by a three-compartment device equipped with electro-regenerated layered IXR bed [47].

#### 4.1.3. Zinc

Different plating processes are performed with zinc. Among them, Zn_3_(PO_4_)_2_ coating layers are produced via phosphate plating baths with H_3_PO_4_. Rinse solutions contaminated by different ions (Zn^2+^, Fe^2+^, ${\mathrm{PO}}_{4}^{3-}$, ${\mathrm{NO}}_{3}^{-}$, etc.) can be treated by ED. For example, 6.5 ppm Zn^2+^ were reduced to ~0.5 ppm, with *E_spec_* of ~3.5 kWh/m^3^ [186]. ED can also be carried out for Zn cyanide electroplating solutions [187].

#### 4.1.4. Chromium

Hexavalent chromium is another heavy metal commonly used in electroplating. It can exist in the form of several ion species (${\mathrm{Cr}}_{2}O_{7}^{2-},{\mathrm{HCr}}_{2}O_{7}^{-},\;{\mathrm{HCrO}}_{4}^{-},\;{\mathrm{CrO}}_{4}^{2-}$) affected by concentration and pH, and is characterized by high toxicity and carcinogenicity.

In ED experiments with Cr(VI) model solutions, operating conditions (i.e., flow rate, initial concentration, pH, and voltage) were varied, attaining removal percentages of ~79% and ~99% for 50 ppm and 10 ppm as initial concentration, respectively, with *E_spec_* ≈ 2–4 kWh/m^3^ [194]. Therefore, the diluted water can be reclaimed. However, the concentrate has to be cleaned from impurities in order to be recycled.

**Figure 6:**
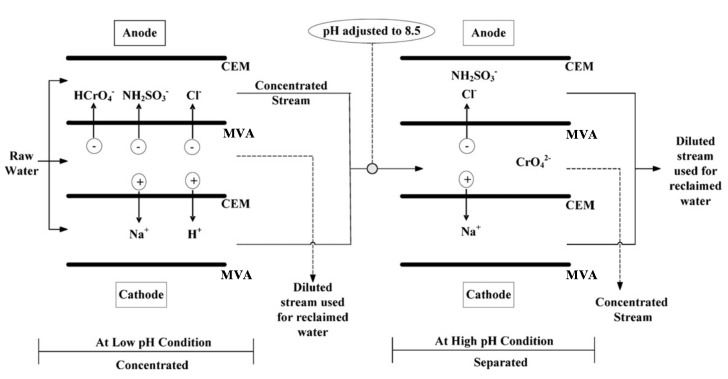
Two-stage selective ED with MVAs for recovering water and concentrate solution from Cr(VI) electroplating wastewater. Reproduced (adapted) with permission from [195], published by Elsevier, 2009.

With this aim, a two-stage selective ED with MVAs was developed [195]. The real wastewater contained Cr(VI) as ${\mathrm{HCrO}}_{4}^{-}$ at pH of 2.2, so that it was possible to concentrate it in the first stage along with other ions. By adjusting the pH of the concentrate at 8.5, the most stable form of chromate was the divalent ${\mathrm{CrO}}_{4}^{2-}$. MVAs of the second stage retained this in the diluate, while letting monovalent ions to pass (Figure 6). From a 418 mg/L Cr(VI) feed, the first stage achieved a maximum concentration factor of ~1.9, while the second one removed Cl^−^ by ~45%.

ED was proposed as a post-concentration step of Cr(VI) diluted solutions after biological treatment [196]. The maximum initial concentration was 100 ppm, simulating the residual concentration of an anaerobic degradation process. A large Cr(VI) retention in the membranes was observed. However, some metal ions were transported to the concentrate channels, leading to a maximum concentration of 570 ppm. The Cr(VI) removal from the feed solution was ~99%, and the volume of concentrate was only ~5.3% of that of the feed. These results suggested the possible reuse of the diluate and concentrate streams.

**Figure 7:**
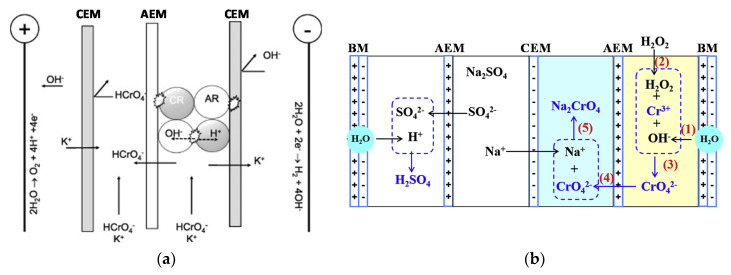
Treatment processes for Cr wastewater: (**a**) EDI with diluate compartment filled by mixed IXRs for separating Cr(VI), water dissociation is highlighted at the bipolar contacts; (**b**) BMED for recovering Cr(III) after oxidation to Cr(VI). Panel (**a**) is reproduced (adapted) with permission from [198], published by Elsevier, 2013. Panel (**b**) is reproduced (adapted) with permission from [203], published by Elsevier, 2020.

Cr(VI) separation by EDI processes was studied [197,198,199,200,201]. For example, EDI (four-compartment device, Figure 7a) was combined with IX [198]. After mixed IXRs saturation, electric current was applied by exceeding by 10% the limiting value, removing 98.5% of 100 ppm Cr(VI) with *E_spec_* ≈ 0.07 kWh/m^3^.

Cr(III) is less hazardous than Cr(VI). However, it is used in various industrial processes, including plating, and thus can be present in waste effluents. Complexation ED removed simultaneously Cr(III) from a synthetic electroplating wastewater and acetylacetone from a model pharmaceutical waste [202]. The two solutions were mixed, obtaining clathrates formation. These charged chelates were concentrated with removal efficiencies of 99.4–99.5% for Cr and 97.8–99.9% for acetylacetone. The proposed strategy of joint treatment was very promising, but further studies should focus on fouling.

Cr(III) was oxidized and recovered as Cr(VI) by BMED [203] (Figure 7b). Cr_2_(SO_4_)_3_ synthetic wastewater (50–1000 mg/L Cr(III)) received OH^−^ from the BM (1), forming ${\mathrm{CrO}}_{2}^{-}$. By adding H_2_O_2_ (2), it was oxidized to ${\mathrm{CrO}}_{4}^{2-}$ (3), which migrated to the recovery chamber (4), where Na^+^ came from the buffer chamber (5). H_2_SO_4_ in the acid chamber was an additional product. Under optimal conditions (e.g., 5.0 g/L Na_2_SO_4_ in 100–1000 mg/L Cr(III) solution) ~70% of Cr was recovered. At 500 mg/L Cr(III), *η* was 67.6% and *E_spec_* was 730 kWh/kg Cr in a stack with three repetitive units. By repeating the experiment three times, the recovery increased to ~88%, because of Cr adsorption and release in/by the AEM (removal decreased from ~98% to ~92%).

Cr(VI) and Cr(III) coexisting in wastewater can be removed by electrodialytic techniques. For example, EDI was able to remove both Cr^3+^ and ${\mathrm{HCrO}}_{4}^{-}$ from a model solution with 100 ppm of both contaminants, but with higher removal for the latter [204]. The separation from salt mixtures with monovalent or divalent ions is more difficult [205].

Industrial treatments of hides and skins require massive consumption of process water containing chemicals for several manufacturing steps. In particular, salts of Cr(III) are used for tanning processes, which produce wastewater at large volumes and with different contaminants (organics, tannins, and salts). Tanning effluent treatment for recycling purposes has to separate Cr(III) from other ions. ED has been used to recover salt water and Cr(III)-solution by taking advantage of the different selectivity of the membranes towards different ions. For instance, filtered spent tanning effluents flowed through ED diluate, where the membranes retained most of Cr(III), which was present in different ionic and non-ionic forms (total concentration of ~0.27%), while removing other ions (Cl^−^ by ~91% and ${\mathrm{SO}}_{4}^{2-}$ by ~51% from concentrations of 3.4% and 3.3% as NaCl and Na_2_SO_4_, respectively) [206]. Fouling and chromium leakage were alleviated by dosing EDTA in small quantities and applying EDR. The overall process economics was promising, as the chromium load was lowered by ~33% and the concentrate salt water was usable in pickling operations.

Another strategy for recovering Cr tanning solutions was developed in two steps with (i) monovalent selective ED and (ii) conventional ED [207]. From model tanning wastewaters with concentrations in the order of ~0.1 eq/L, MVCs retained Cr(III) in the diluate and separated NaCl in the concentrate. Then, chromium was concentrated by conventional ED. Optimal pH was around 3, avoiding precipitation and also the competing transfer of H^+^. Thus, *η* approached 100% at the second stage. Further experiments on the first step with salt mixtures (Na_2_SO_4_, MgCl_2_ and CaCl_2_) showed that most of Na^+^ could be removed (*η* ≈ 70%), with a global *η* of ~97% [208].

Treatment of tannery wastewaters has been based on integrated processes for water recycling where the first operation degraded organics. Photoelectrochemical oxidation [209] or electrocoagulation [210] have been proposed for this purpose, followed by ED or BMED, respectively. The former combined process removed 87.3% of COD and more than 98.5% of ions (Cr was present only in traces in the raw effluent, i.e., with concentration < 0.01 mg/L); the latter combined process removed ~90% of COD and almost all Cr (from 570 mg/L), ammonium and colour, with *E_spec_* of 14–30 kWh/m^3^.

#### 4.1.5. Cadmium

Cadmium electroplating is a galvanic process performed via alkaline baths with cyanide, and ED represents again an option as recovery process from waste effluents [211]. A simulated wastewater (CdO, NaCN and NaOH at 0.0089, 0.081 and 0.018 mol/L, respectively) flowed through the diluate of a five-compartment ED module. Maximum removals of 86% of ${\mathrm{CdCN}}_{4}^{2-}$ (which was the predominant complex) and 95% of CN^−^ were achieved. However, the process efficiency was affected by Cd(OH)_2_ precipitation on the CEM at the diluate side.

Solutions with similar composition simulating diluted baths (Cd concentration between 1 and 3 g/L) were used in further experiments [212]. No precipitation was observed, with removals of 21.6% and 46.1% for ${\mathrm{CdCN}}_{4}^{2-}$ and CN^−^, respectively (*η* of 13.2% and 59.6%). The process was performed in the same way when feeding the model wastewater in both diluate and concentrate channel, i.e., in a configuration more similar to conventional ED. To recycle the concentrate into electroplating baths, while avoiding efficiency losses and risks of membrane damage, the diluate feed was changed four times. This led to a concentrate concentration increase by 56% for cadmium and 250% for cyanide.

The selective separation between metal ions by EDI, referred to as electropermutation, was achieved in a solution containing Cd^2+^ and Na^+^ [213]. A cation-exchange resin, modified by natural polyelectrolyte, fixed selectively Cd^2+^ in the central channel of a five-compartment electro-regenerated unit.

#### 4.1.6. Lead

ED has been tested for wastewater containing lead, which originates from industrial processes (regarding, for example, batteries, electronics, printing pigments, explosives, metallurgical processes). With a Pb(NO_3_)_2_ model solution, experiments assisted by analysis of variance assessed the effect of concentration (100–1000 ppm), flow rate, voltage and temperature [214]. The separation was affected mostly by the flow rate, and reached ~95% under optimal conditions. Modelling tools validated against experiments showed that the artificial neural network model was more accurate than the simplified model, the former being able to predict the non-linearity of transport phenomena and thus of ED [215].

Another important operating parameter is the pH. Optimal values of 3–5 were found in experiments with a solution at 800 mg/L Pb^2+^ performed with an ED pilot stack [216]. Lower voltages were effective in increasing *η* (up to ~35%) and maintaining low *E_spec_* values (~0.1 kWh/m^3^), while higher voltages could regenerate the membranes from the adsorbed ions. The same ED stack was used in a combined treatment process developed with electrolysis and a further ED unit for adsorption in CEMs [217]. The initial concentration had the most significant effects on the ED performance. With optimized parameters (Taguchi method), ED reduced the initial Pb^2+^ concentration of 600 mg/L to ~16 mg/L in the ED diluate. This was further reduced by adsorption to ~1 mg/L, reaching the target required by the Chinese regulation. The electrolysis process recovered ~90% of Pb via cathode deposition from the ED concentrate. Other ED experiments reduced the concentration from 500–1000 mg/L to 1–2 mg/L [218]. Under optimal operating conditions, high values of *η* were obtained (82.8–72.4%) with *E_spec_* of 0.16–0.36 kWh/m^3^. Despite several promising results, the feasibility for real Pb-wastewaters has still to be demonstrated.

#### 4.1.7. Mixtures and Other Heavy Metal Ions

Real industrial wastewaters often contain mixtures of metal ions (either ions with similar concentration or impurities), and ED processes can effectively recover water, concentrate ions, and, in some cases, separate different ion species from each other.

Brass (Cu and Zn) electroplating was evaluated by cyanide-free baths with EDTA, by using ED for treating the rinsing water [219]. By adjusting the ED concentrate concentration to that of the original bath, good deposits were obtained. In the overlimiting regime, the recovery of metals and EDTA was more advantageous [220]. The rinse water was prepared with 0.0006 M CuSO_4_, 0.0014 M ZnSO_4_, 0.0015 M EDTA, 0.03 M NaOH (conductivity of 5.3 mS/cm), corresponding to 1% of the concentrations in the bath. The diluate solution was replaced once reached the conductivity of ~0.2 mS/cm, and the concentrate solution was not replaced to maximise its concentration. Cu, Zn and EDTA were concentrated more with overlimiting operation (concentration factor of ~3.45 against ~2.94 with underlimiting operation), likely due (i) to water dissociation generating protons that reacted with complexes and insoluble species, and (ii) to electroconvective mass transfer enhancement. Moreover, fouling and scaling were reduced. 

High removals were achieved by ED from a real electroplating effluent containing Ni^2+^ and Cu^2+^ (~23 mg/L for both) [221]. A tertiary treatment line was developed for reclaiming a plating wastewater effluent with a mixture of heavy metal ions at low concentration (~1 mg/L) [222]. Microfiltration (MF) and ultrafiltration (UF) removed organics and suspended solids, then ED desalination was conducted, and finally, the concentrate was treated by nanofiltration (NF) or reverse osmosis (RO) to increase water recovery. The ED step removed 97% of Cr^3+^, Cu^2+^, and Zn^2+^, 95% of ${\mathrm{SO}}_{4}^{2-}$ and Cl^−^ (from initial concentration of 1000 mg/L), and 85% of COD (300 mg/L initial concentration). In another study, ED was conducted after chemical precipitation of a real Cr(VI) electroplating wastewater (19 mg/L) with minor concentrations of Cu^2+^, Zn^2+^ and Cd^2+^ [223]. Cr(VI) was reduced to Cr^3+^ by Na_2_S and FeCl_2_, and then precipitated with other metal ions by NaOH dosage (pH = 9). Then, ED diminished the Cr(VI) concentration in the effluent (1–8 mg/L) by up to more than 95%, thus producing a water reusable for rinsing operations. By treating synthetic rinse waters of Cd cyanide electroplating (1000 mg/L Cd) contaminated by either Cu (50 mg/L), Fe (50 mg/L) or Cr (100 mg/L), the non-selective transport made the ED concentrate not reusable for electroplating baths [212].

**Figure 8:**
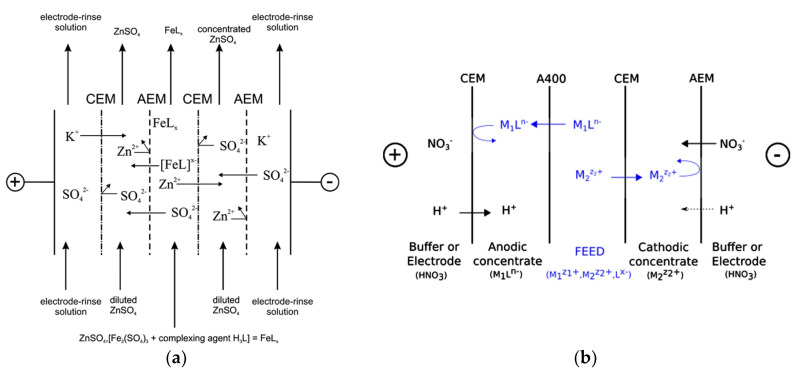
Schemes of complexation-enhanced ED: (**a**) Zn^2+^ recovery from a model Fe^3+^-contaminated electroplating bath by Fe-citrate neutral complex retention; (**b**) selective separation of metal cations (Ag^+^/Zn^2+^ or Cu^2+^/Cd^2+^). The feed solution contains initially the two metal ions $M_{1}^{z_{1}+}$ and $M_{2}^{z_{2}+}$, and the ligand $L^{x-}$, which forms complexes only with $M_{1}^{z_{1}+}$ (i.e., $M_{1}L^{n-}$ ) in situ. The A400 AEM allows for the transport of anion complexes of ~400D without fouling. Panel (**a**) is reproduced (adapted) with permission from [224], published by Elsevier, 2018. Panel (**b**) is reproduced with permission from [226], published by Elsevier, 2017.

However, metals from a mixture can be selectively separated in different channels by complexation–ED. A simulated Zn electroplating bath was prepared with 48.9 g/L Zn^2+^ and 1 g/L Fe^3+^, and treated by testing different chelating agents [224]. These solutions were circulated through the diluate of a five-compartment ED stack with two concentrate channels (Figure 8a), obtaining the separation between Zn^2+^ and Fe complexes. The process was more efficient when heterogeneous IEMs and citric acid were used. A high retention of Fe (~92%) in the feed channel was caused by the generation of an electrically neutral citrate complex, while ~87% of Zn^2+^ was removed with *η* ≈ 85%. These results suggest that recovering concentrate solutions from contaminated baths can be feasible in conventional ED with one diluate and one concentrate. Further experiments confirmed these results [225], while finding problematic the selective separation for a Zn^2+^ solution contaminated by Cu^2+^. This occurred due to a partial formation (~65%) of Cu–citric acid anions.

By taking advantage of the actual formation of anion chelates, complexation–ED was carried out by a three-compartment configuration (Figure 8b) in order to separate metals from mixtures of Ag^+^/Zn^2+^ or Cu^2+^/Cd^2+^ [226]. The two ions of each mixture were transported to two different concentrate compartments. In fact, Zn or Cd formed anion complexes, instead Ag^+^ or Cu^2+^ persisted as free ions. EDTA was found to be the best among various complexing agents, allowing for removal percentages higher than 99% from initial concentrations of 0.1–1 meq/L with *E_spec_* between 0.28 and 0.55 kWh/m^3^, thus enhancing the process compared to previous results reported in [227]. BMED was suggested for separating the complexed cation and regenerating the ligand.

Ni and Co were separated by complexation with EDTA [178]. A Ni-EDTA negative complex was preferentially formed and retained, while Co^2+^ ions migrated through the CEM.

Waste mixtures with heavy metal ions can be purified by EDI processes. A five-compartment EDI device with electro-regenerated cation and anion IXRs in separated beds was tested with a real electroplating waste rinse with Ni^2+^, Cu^2+^, Zn^2+^, Cd^2+^ and Cr^3+^ [46]. A similar unit was tested with a waste solution from a Zn electrolysis process containing Zn^2+^ and other metals [48].

Electrodialytic technologies have been studied for treating various other industrial waste effluents containing metal ions. H_2_SO_4_-CuSO_4_ solutions with impurities (As(III), As(V) and Sb(III)) typical of Cu-electrorefining electrolytes were reclaimed via ED by separating and concentrating metal ions [228]. Similarly, a Cu–electrowinning model solution containing 50 g/L H_2_SO_4_ and 9 g/L Cu^2+^ (as CuSO_4_ salt) with 0.5 g/L Fe^2+^ impurities was reclaimed by removing 96.6% of copper and 99.5% of iron with *E_spec_* ≈ 1 kWh/kg [229]. Cr(VI) was recovered as H_2_CrO_4_ by BMED from chromite ore processing residue (chromate production byproduct) [230]. The waste effluent contained a mixture of ~3728 mg/kg Cr(VI) and ~2650 mg/kg Cr(III), along with other metal ions (Fe, Al, As, etc.). The BM-AEM configuration was used, and MF membranes were placed in the wastewater compartment to protect the BM and the AEM from clogging. Recoveries of ~90% were obtained with *η* of 2.3% *E_spec_* of 395 kWh/kg. 

**Figure 9:**
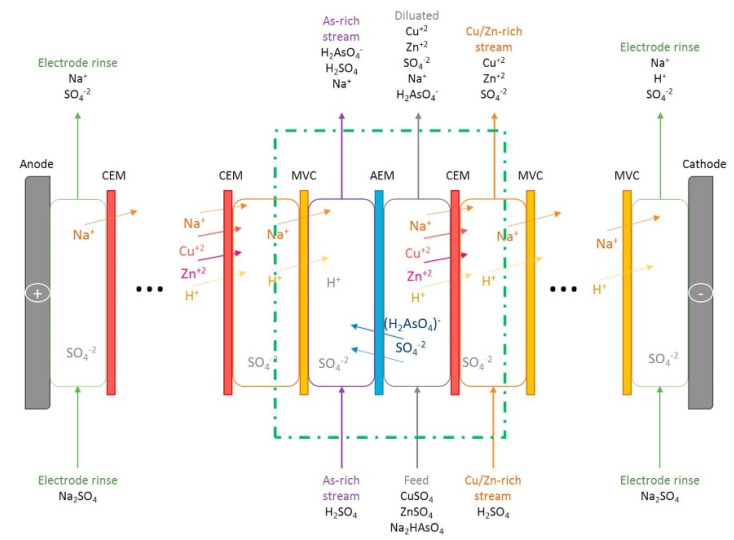
SED configuration for recovering Cu^2+^ and Zn^2+^ from acidic metallurgical wastewater containing As(VI). Reproduced (adapted) with permission from [231], published by Elsevier, 2018.

Selectrodialysis (SED) with MVC was used with synthetic solutions simulating an acidic metallurgical wastewater (pH = 2.3) with 47 mM CuSO_4_, 146.8 mM ZnSO_4_, and 31.6 mM Na_2_HAsO_4_ [231] (Figure 9). The process recovered 80% of Cu^2+^ and 87% of Zn^2+^ in a solution, and 95% of As(V) in another solution, at *η* of ~38% for Cu^2+^ and Zn^2+^ and *E_spec_* of ~2.6 kWh/kg for their salts. The solution rich in Cu^2+^ and Zn^2+^ was pure by 99.8% (over 80% due to Zn^2+^), while the product rich in As(V) contained a comparable concentration of Zn^2+^. To solve this problem, the authors suggested recirculating the As(V) product to the feed.

ED arrangements either with or without MVAs were proposed for reclaiming alkaline gold mine wastewater containing heavy metals (copper and zinc), sodium and cyanide [232].

**Figure 10:**
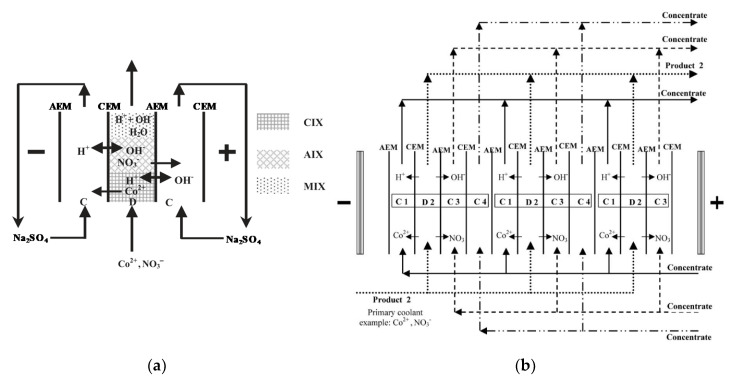
EDI configuration with diluate compartment filled by layered bed for the treatment of a Co^2+^ solution simulating the primary coolant from nuclear power plants: (**a**) sketch of the bed packing; (**b**) stack with three repetitive units with four channels each. Reproduced (adapted) with permission from [233], published by Elsevier, 2004.

EDI is suitable for purifying nuclear power plants’ primary coolants, which contain low concentrations of Co^2+^ [233,234]. Among several arrangements tested with model solutions (e.g., 0.34 mM), a five-compartment EDI module was developed by using a layered bed within the diluate to prevent the precipitation of metal hydroxide, remove both anions and cations, and control the pH (Figure 10a) [233]. Starting from this EDI arrangement, a stack with four-compartment repetitive units was assembled (Figure 10b). Removals of over 99% at *η* ≈ 30% and *E_spec_ =* 14 kWh/m^3^ were obtained. Other experiments conducted with a four-compartment EDI device removed up to ~99.9% of Cs^+^ from model waste solutions (e.g., 50 mg/L) [235]. Different radionuclides (Cs^+^, Sr^2+^ and Co^2+^) in traces were removed by 77.1–99.7% [236]. Th^4+^ was removed at rates of up to ~99% (from 30–90 mg/L) in experiments optimized by response surface methodology [237]. Overall, EDI processes are very promising for treating low radioactive effluents.

### 4.2. Regeneration of Acid/Base, Salt Conversion

Many manufacturing processes produce large quantities of acidic/alkaline waste streams, and their neutralisation is commonly practiced for disposal. In other cases, spent alkaline/acidic solutions result in waste salt streams. In all cases, economic and environmental benefits can be drawn from reuse/recycling approaches. From this perspective, electrodialytic processes (mainly ED and BMED) can be used for treating different industrial effluents, such as acidic wastewaters with heavy metal ions from pickling ant other processes (Section 4.2.1), waste solutions without heavy metal ions (Section 4.2.2), spent alkaline solutions from flue gases chemical absorption (Section 4.2.3), and wastewaters with organic matter, including organic acids (Section 4.2.4).

#### 4.2.1. Effluents with Heavy Metal Ions

Waste acidic effluents are produced form pickling and other processes of metal manufacturing and metallurgical industry. In particular, pickling is a surface treatment that removes impurities (oxides, rust, and others) before metal pieces go through painting, plating, etc. The main application is steel acid pickling. Pickle liquors contain sulphuric, hydrochloric, nitric or hydrofluoric acid, which react with oxides, thus dissolving metal ions. They are regarded as being spent once the acid concentration diminishes by 75–80%, and the metal concentration rises to 150–250 g/L [238]. Pickling processes produce large quantities of spent solutions. For instance, steelwork plants generate ~3 × 10^5^ m^3^/y waste pickle liquors in the only Europe.

The regeneration (recovery and purification) of pickling operations effluents can be accomplished by several methods, including ED for acid concentration and metal separation [238,239]. For example, 60–70% of H_2_SO_4_ was recovered from a pickling rinse water (9100 ppm) with a ten times increased acid/iron concentration ratio (from 7.4:1 to 74.6:1) [186]. Optimal operating conditions and selective membranes are crucial [240]. Proton leakage through AEMs, which limits the acid concentration [241], can be alleviated by purposely developed proton-blocking membranes [240,242,243,244,245,246,247,248]. On the other hand, the passage of metal ions across the membranes may impair the concentrate purity [249] and cause fouling [240]. However, using MVCs that retain multivalent metal cations allows for the acid recovery with high purity [250].

**Figure 11:**
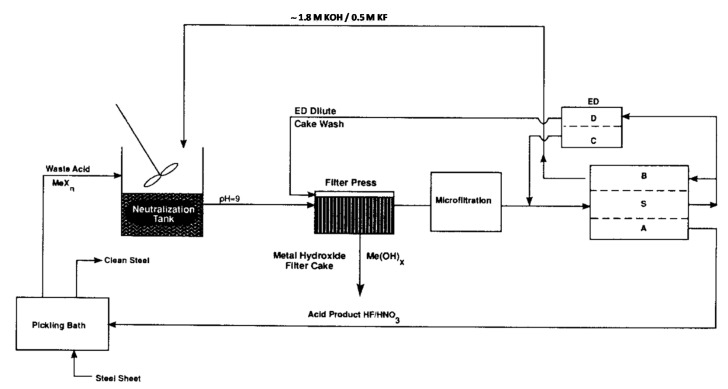
Flowsheet of the pickling liquor recovery by BMED-ED coupling. Reproduced (adapted) with permission from [38], published by Elsevier, 1991.

After neutralisation of the spent pickling solution and precipitation of metals, BMED can regenerate the acid stream in combination with an ED salt concentration step [251]. A BMED-ED integrated pilot has been used since 1987 [34] in an industrial treatment plant at the Washington Steel Corporation facilities (Pennsylvania) [37], where a pickling solution with mixed acids (8–15 wt% HNO_3_ and 2–5 wt% HF) was used. The process scheme is depicted in Figure 11, and can be described as follows [37]. Metals in the spent liquor were removed by neutralisation/precipitation (KOH dosage) and filtration. The KF/KNO_3_ solution obtained (1.1–1.5 M, with metal ions at concentration < 1 ppm) went through the salt compartment of BMED, which produced the base used for neutralisation, and the mixed acids (HF + HNO_3_) recycled into the pickling bath. ED recovered water (for filter cake washing) and salt from the BMED diluate (0.3–0.5 M) stream. The base was diluted with a fraction of the BMED diluate. The BMED and ED stacks were assembled with 25 and 15 cell units, respectively, totalling 2.33 m^2^ and 1.4 m^2^ of membrane area. During a long-term run with 240 L/day waste acid, *η* was ~80% for acid and base, and remained quite stable over time, while *E_spec_* was ~0.25 kWh/L acid product (180 L/day). 93% of F^−^, 99% of ${\mathrm{NO}}_{3}^{-}$, and 96% of K^+^ were recovered. The economic analysis for a scaled-up system (6 × 10^6^ L/y) found high investment costs, but with a 4-year payback period.

Several metallurgical processes produce spent acidic solutions with metal ions, and the reclamation via ED or BMED has been tested. ED concentration of a spent solution from a metallurgical industry, containing Ni^2+^, Cu^2+^ and other ions, recovered more than 80% of H_2_SO_4_, showing the impact of the membranes (MVMs, proton-blocking AEMs) [252]. Similarly to the example previously reported for waste pickling effluents, BMED can produce acid and base from waste mixtures of heavy metals and salts, after pre-treatment removing metals. From a Ni washing wastewater, crystallisation (fluidized pellet reactor) removed up to 74% and 94.4% of Ni^2+^ and Ca^2+^, respectively (with filtration), thus minimizing scaling in the BMED [253]. The feed salt solution of BMED contained ~45 g/L Na^+^ and ~80 g/L ${\mathrm{SO}}_{4}^{2-}$ as main ions, with ~10 mg/L Ni^2+^, ~16 mg/L Ca^2+^, and other minor components. *η* was 69% and 80% for acid (H_2_SO_4_) and base (NaOH), respectively, while *E_spec_* was 5.5 kWh/kg acid and 4.8 kWh/kg base. In a long-term test, acid and base at 1.76 N and 2.41 N, respectively, were obtained starting from 0.2 N, with small scaling.

**Figure 12:**
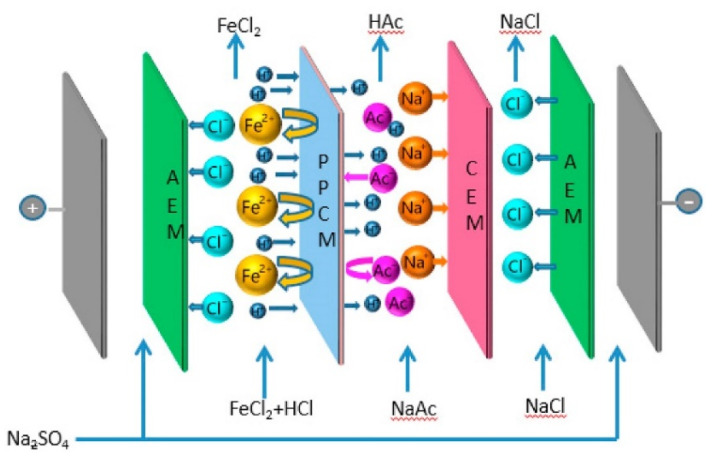
Schematics of ion substitution ED with three-compartment repeating units for the conversion of metal finishing waste acid into organic acid. Reproduced with permission from [255], published by Elsevier, 2019.

An alternative way for reclaiming acidic streams from metal finishing is the conversion to organic acid via ion substitution ED [254]. The wastewater feed maintains metal ions retained by an MVC, and releases protons and anions to the organic salt stream and the inorganic salt stream, respectively (Figure 12). From a model waste acid (0.4 M HCl, 0.1 M FeCl_2_) and a 0.3 M sodium acetate solution, acetic acid at high purity was produced (0.2 mM Fe^2+^) with average *η* of 91%. A proton selective composite CEM was then developed, showing a possible process enhancement [255].

**Figure 13:**
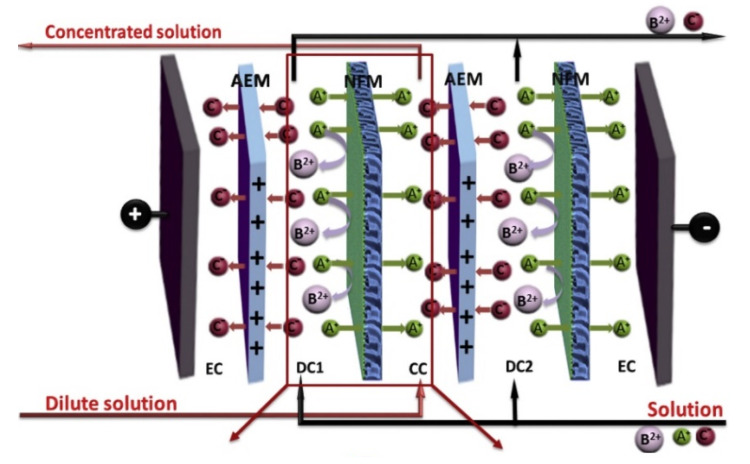
Modified ED equipped with NF membrane in place of CEM. Monovalent cations A^+^ (e.g., H^+^) can move across the NF membrane, while divalent cations B^2+^ (e.g., Zn^2+^) are retained. Reproduced with permission from [261], published by Elsevier, 2016.

Other ED applications regard spent acids produced by Zn hydrometallurgy [256,257,258]. Again, in order to prevent acid and metal leakage, proton-blocking AEMs and MVCs are crucial elements for performing feasible recovery processes via ED [256]. MVCs were prepared by different methods [259,260], reaching $P_{Zn^{2+}}^{H^{+}}$ of 34.4 [260]. An interesting alternative is provided by replacing CEMs with NF membranes (Figure 13) [261]. Homemade NF membranes were used for acid recovery from a Zn^2+^-containing synthetic solution (diluate feed with 0.5 mol/L H_2_SO_4_ and 0.23 mol/L ZnSO_4_, concentrate with 0.05 mol/L H_2_SO_4_). The modified ED system exhibited better performance compared to the stack equipped with MVCs, increasing the permselectivity $P_{Zn^{2+}}^{H^{+}}$ from 15 to 354.

BMED of acidic raffinate from Cu ore hydrometallurgical processing was proposed [262]. From the raffinate (11,800 mg/L Fe, 336 mg/L Zn, 135 mg/L Cu, etc.) heavy metals were separated (from ~70% to ~99%) as precipitates in the base, and ${\mathrm{SO}}_{4}^{2-}$ (45.2 g/L) was transported to the acid (by ~86%), thus recovering H_2_SO_4_, albeit with some impurities. With *E_spec_* below 0.1 kWh/L and high values of *η*, the treated raffinate reached a metal concentration below 100 mg/L, thus being reusable as leachate.

ED tests were performed to recover nitric acid from rinsing-wastewater from aluminium anodizing industry (acidity of 4085 mg/L CaCO_3_) [263]. Despite some issues of Al precipitation and leakage, most of the waste acid was recovered with a conductivity removal of ~86–91% and *E_spec_* values of ~0.11–0.3 kWh/mol acid. Acid recovery can be obtained also by combined membrane processes. HCl from acidic wastewater produced by aluminium foil industry was recovered by integrating diffusion dialysis and ED (model solution with 1.35 mol/L HCl and 0.15 mol/L AlCl_3_) [264] or BMED (model solution with 4.7 mol/L HCl and 0.59 mol/L AlCl_3_) [265], showing that cost-effective schemes can be devised. In the former combined process, up to ~75% of HCl was recovered with a metal leakage of ~12%. In the latter, after acid recovery by diffusion dialysis, the dialysate was fed to the base compartment of the BMED, thus allowing for the aluminium recovery.

Experiments showed the ED effectiveness for acidic wastewater with various metal ions (including Cu, Fe, Zn, Cd and As) from the chalcopyrite (CuFeS_2_) mining industry [266]. An effluent from the SO_2_ wet purification process was cleansed from metal ions by IX, then was fed into the ED diluate to concentrate H_2_SO_4_ in the concentrate (95–98% recovery from a feed concentration of ~17 g/L) and to provide reusable water.

Sulphuric acid and sodium hydroxide were produced by BMED from IX spent regenerant (containing ~0.75 M H_2_SO_4_, ~0.55 M Na_2_SO_4_, and metal ions) coming from hydroxy acids liberation from alkaline kraft black liquor [267]. Feeding acid and base compartments with initial concentrations of 0.1 M, the BMED provided a solution with 1 mol/L H_2_SO_4_ at 95% purity, and a solution with 0.79 mol/L NaOH at 93% purity, thus presenting a promising perspective to reduce the chemicals consumption in the overall process.

A rare example of ED application for base recovery from an alkaline solution was reported in a study on a synthetic wastewater of 0.1 mol/L Na_2_WO_4_ and 1 mol/L NaOH [268]. Composite AEMs were prepared and tested, showing OH^−^ recovery ratios up to ~65%, *E_spec_* of ~7 kWh/kg, and low tungstate leakages (5–14%).

#### 4.2.2. Effluents without Heavy Metal Ions

**Figure 14:**
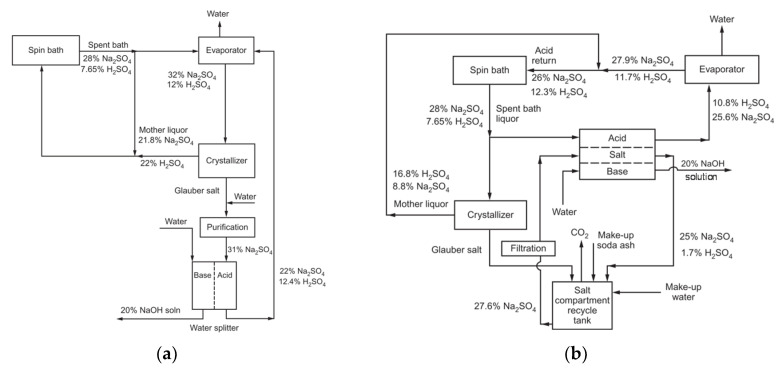
Rayon process flowsheet with BMED: (**a**) Two-compartment configuration (BM-CEM); (**b**) Three-compartment configuration. Panel (**a**) is reproduced with permission from [37], published by Elsevier, 1988. Panel (**b**) is reproduced with permission from [38], published by Elsevier, 1991.

BMED can recover acidic/alkaline solutions from several saline wastewaters produced in industrial processes. In rayon production plants, BMED can restore the acidity of spin baths by converting part of the Na_2_SO_4_ from spent baths into H_2_SO_4_, and produce NaOH reusable in cellulose dissolution [37,38] (Figure 14). The crystallisation of Na_2_SO_4_ produced Glauber salt. After purification and dissolution in water, the solution passed through the acid compartment of a two-cell BMED unit or through the salt compartment of a three-cell BMED unit (receiving a spent bath portion in the acid compartment). *η* values of 80–95% were reported. For a production of 10,000 Mt/y NaOH, a payback period of 2−5 years was estimated.

BMED was cost-effective for desalinating cooling tower blowdown and producing acid and base, which could be reused on-site, e.g., for IXRs regeneration [269]. From NaCl synthetic solutions (48–390 mM, with the lower part of the range being representative of cooling tower blowdown), 73–81% of salt was converted into acid/base, at *η* higher than 75% and *E_spec_* of 0.02–0.09 kWh/mol. The maximum estimated cost (12.6 $/kmol, under the assumption that the total cost is 1.7 times the energy cost) was less than the minimum cost of purchase (21 $/kmol).

The technical feasibility of BMED was proven for recycling several other saline wastewaters. Tests on BMED stacks were performed with NH_4_NO_3_ nuclear fuel processing effluents [270,271] or NaNO_3_ dying industry effluents [272] to produce HNO_3_ and NaOH. Wastewaters from UF_6_ production can be recycled as HF and KOH [251]. Phosphogypsum (CaSO_4_) by-product from phosphoric acid production can be converted by NaOH into Ca(OH)_2_ and Na_2_SO_4_, thus splitting the salt into base and sulphuric acid via BMED [273]. Other applications were proposed to convert NH_4_Cl into HCl and NH_3_ [274,275], NH_4_HCO_3_ into NH_3_ and CO_2_ [276], Na_3_PO_4_ into H_3_PO_4_ and NaOH [277], NaBr into HBr and NaOH [278,279], Na_2_SO_4_/(NH_4_)_2_SO_4_ into H_2_SO_4_ and NaOH/NH_3_ [280], NaCl/KCl into HCl and NaOH/KOH [281], boron into boric acid [282,283,284].

**Figure 15:**
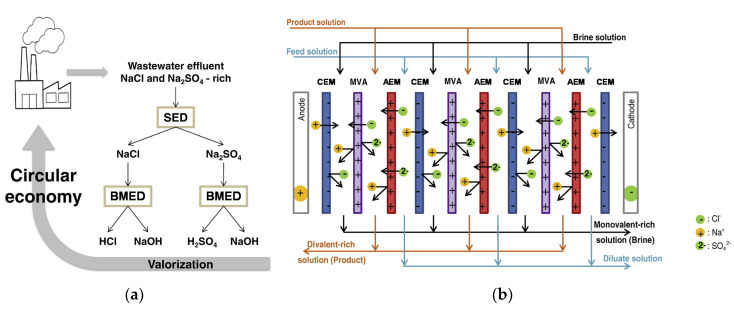
Valorisation of salt mixture wastewater by SED-BMED integration: (**a**) Scheme of the process; (**b**) Sketch of SED with MVAs. Reproduced (adapted) with permission from [285], published by Elsevier, 2016.

A SED-BMED coupled process was developed for waste salt mixtures (NaCl and Na_2_SO_4_, originated from dye synthesis) conversion into NaOH and separated acids (HCl and H_2_SO_4_) [285] (Figure 15). Different feed solutions were used, with sulphate concentration from 26 to 840 mM and chloride concentration from 63 to 497 mM. SED with MVAs fractionated the salts into two product streams at purity of ~90% for Cl^−^ and over 90% for ${\mathrm{SO}}_{4}^{2-}$. From these solutions, the BMED processes yielded pure NaOH and acid solutions rich in HCl or H_2_SO_4_ by 87% or 93%, respectively. *E_spec_* was ~6.4 kWh/kg product on average for the SED with solutions at medium to high concentration, while it was ~5 kWh/kg NaOH for the BMED.

#### 4.2.3. Spent Solutions from Chemical Absorption of Flue Gases

**Figure 16:**
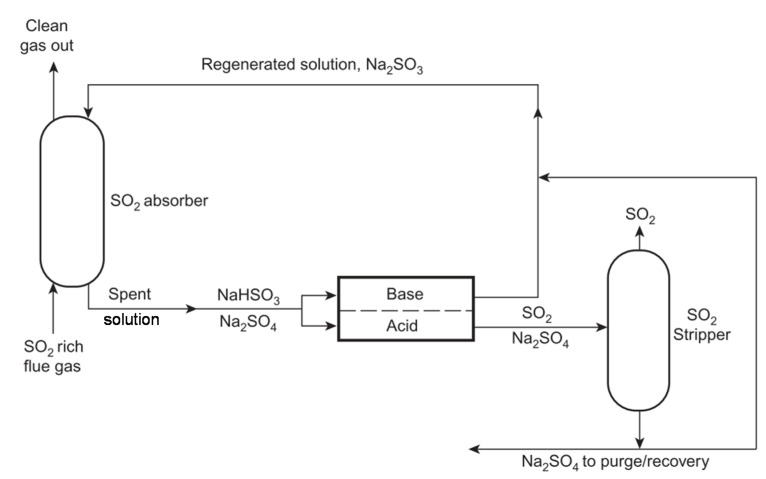
BMED Soxal^TM^ process for flue gas desulfurisation. Reproduced (adapted) with permission from [38], published by Elsevier, 1991.

Chemical absorption through wet scrubbers is used in treatment lines for waste gases produced by combustion at power plants (e.g., coal-fired) and by other processes. BMED or ED can be adopted for regenerating spent alkaline or acidic solutions from flue gases chemical absorption, thus recycling the absorbent for the scrubbing tower. Two different processes were developed to recover spent alkaline absorbents for SO_2_. One of them was based on the three-compartment BMED fed by the Na_2_SO_4_ solution from the stripper to produce NaOH, which is reused for absorption [8,286]. The other process was developed by the two-compartment BMED unit with BM and CEM [286,287], and took the name of Soxal^TM^ as an industrial process [38] (Figure 16). The spent solution is an NaHSO_3_/Na_2_SO_4_ mixture that is converted into a regenerated stream of Na_2_SO_3_ (in the base channel), and into a stream with SO_2_ (in the acid channel) that is then stripped. These regeneration processes led to significant economic advantages, exhibiting *η ≈* 90% and *E_spec_* ≈ 1.3 kWh/kg base [9].

The coupling of IX with BMED was tested to treat limestone-gypsum wet flue gas desulfurisation wastewater after chemical precipitation of Ca^2+^ and Mg^2+^ [288]. A synthetic wastewater (35–140 g/L NaCl and Na_2_SO_4_ mixed salts at mass ratio of 2:1, and 40–250 mg/L Ca^2+^ or Mg^2+^) was softened by chelating IXRs to remove residual hardness, thus preventing scaling. The BMED obtained 99.3% pure acid and 99.0% pure base. The former will regenerate the saturated IXRs, and the latter will be dosed for the precipitation step.

BMED powered by solar organic Rankine cycle was tested with saline wastewater from flue gas desulfurisation in fluid catalytic cracking [289]. Experimentally assisted simulations were performed for a process in which the NaOH absorption spent solution contains ${\mathrm{HSO}}_{3}^{-}$ and ${\mathrm{SO}}_{3}^{2-}$ that are oxidized (in an aeration tank) to ${\mathrm{SO}}_{4}^{2-}$. Thus, the Na_2_SO_4_ solution was treated by BMED. Simulation results showed that the salt solution was converted into H_2_SO_4_ (7.6 wt%) and NaOH (6.4 wt%), by reducing the salt content in the wastewater from 8.0 wt% to 0.37 wt% with *η ≈* 52% and *E_spec_* ≈ 2.7 kWh/kg salt.

A two-step ED treatment removed fluoride and chloride from ammonia-based flue gas desulfurisation slurry [290]. The slurry was pre-treated by MF and IX to remove fly ash and metals. A synthetic solution (10,000 mg/L F^−^, 20,000 mg/L Cl^−^, and 50% (NH_4_)_2_SO_4_, by dissolving NH_4_F, NH_4_Cl, and (NH_4_)_2_SO_4_) was also used for comparison purposes. After the first stage, Cl^−^ was almost completely transported to the concentrate (tap water) with *η* ≈ 54% and *E_spec_* ≈ 0.9 kWh/kg, while a small amount of F^−^ was removed, mainly remaining with ammonium and sulphate in the slurry (diluate). A double second stage was then carried out to treat the two outlet solutions from the first stage. One was fed with the previous concentrate in the diluate channels to further separate Cl^−^, another was fed with the previous diluate in the diluate channels to separate F^−^ and purify the slurry. The MVAs were crucial for ${\mathrm{SO}}_{4}^{2-}$ retention. A solution with Cl^−^ purity larger than 95% and a solution with F^−^ maximum purity of 51.4% were obtained.

ED can be cost-effective for regenerating spent alkanolamine effluents used for H_2_S absorption, by removing inorganic and organic degradation by-products that form heat stable salts [291,292]. The estimated cost was 14.6 $/ton with *E_spec_* of 39.4 kWh/ton for a spent amine wastewater from the H_2_S desulfurisation stripper of a thermoelectric factory (20.38 wt% N-methyldiethanolamine and 2.54 wt% salts, 36 L/day [291]). The selective removal of heat stable salts along with the minimisation of N-methyldiethanolamine loss was attained by developing ED or EDI stacks equipped with three-compartment repeating units [293]. This configuration comprised: CEM, concentrate, AEM, diluate (with or without anion-exchange resin), AEM, NaOH solution. Hydroxyl ions of the base compartment migrated to the diluate and reacted with binding amine, and thus neutral amine was regenerated. Moreover, the hydrolysed (cationic) amine was retained in the diluate. This solution was depleted in anions migrating to the concentrate. The spent solution coming from an H_2_S desulfurisation stripper in an integrated gasification combined cycle power plant contained 21.06 wt% N-methyldiethanolamine and 5.19 wt% heat stable salts, at pH of 9.4 and conductivity of 12.32 mS/cm. Salts were removed by ~94%, 86% and 65% in the three-compartment EDI, three-compartment ED, and conventional ED, respectively, which exhibited losses of amine of ~3.8%, 5.6% and 21.1%, with *E_spec_* of 71.7, 66.6, and 56.25 kWh/m^3^ wastewater and estimated total cost of 0.88, 0.92, and 1.04 US$/kg heat stable salt (treatment of 48 L/h). Moreover, AEM fouling was reduced in the EDI.

**Figure 17:**
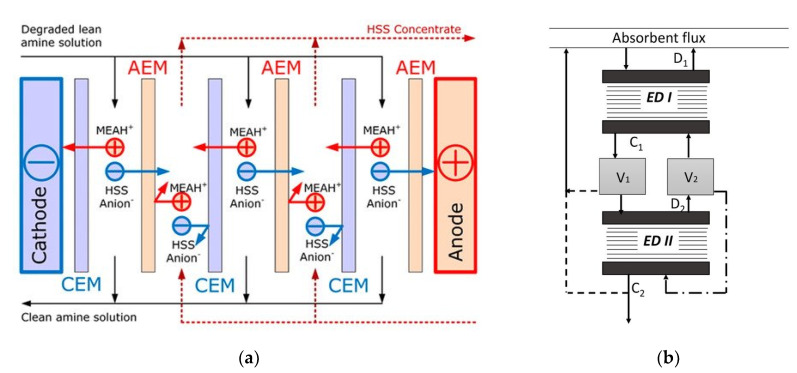
ED for heat stable salt removal from monoethanolamine solvent: (**a**) principle of the process; (**b**) flow scheme of the two-stage ED, where the concentrate stream after the first stage goes to the second stage to reduce monoethanolamine loss. Reproduced from [295].

Similarly, ED can regenerate spent alkanolamine absorbents for CO_2_. Pilot-scale studies were conducted with a spent solution at 30 wt% monoethanolamine, showing stable performances during long-term operations [294]. The effect of CO_2_ loading (from 0 to 0.2 mole/mole amine) on heat stable salts (48 meq/L) removal from monoethanolamine-based solvent (30 wt%) was studied by two-stage ED [295] (Figure 17). An increase in recovery of salts was observed as the CO_2_ concentration decreased, due to the smaller content of amine charged species and their lower competitive transport. An optimum CO_2_ loading of 0.1 mole/mole amine and the associated *E_spec_* of 25.9 MJ/kg solvent were estimated, considering that the change in CO_2_ loading would require additional power for further solvent regeneration in the stripping column.

A BMED stack with BM-CEM configuration recovered CO_2_ and regenerated NaOH from model carbonate solutions [296], recording *η* values of 46–80% and *E_spec_* values of 1–3 kWh/kg CO_2_. The economic analysis highlighted the importance of the membrane cost for the process competitiveness. A coupled system was developed with the BM-AM two-cell BMED configuration and a hollow fibre membrane contactor aiming at regenerating spent absorbers (1 M monoethanolamine, piperazine or NaHCO_3_), removing heat stable salts and separating CO_2_ [297]. From BMED, the alkaline stream was recirculated into the flue gas absorber, while the acidic stream was recirculated into the membrane module for CO_2_ separation. The developed mathematical model predicted *E_spec_* = 2 MJ/kg CO_2_, but the actual consumption in the experiments was 3–4 times higher due to the low *η* (~40%).

#### 4.2.4. Effluents with Organic Matter

Salt or acidic wastewaters may have organic compounds. BMED or ED have been studied for acid/base recovery and organic matter separation, despite such solutions are complex and bring possible issues of organic fouling.

In a BMED stack used to regenerate ${\mathrm{NH}}_{4}^{+}$ and H_2_SO_4_ from glutamate wastewater, CEM scaling at the base side was caused by Ca^2+^ and Mg^2+^, along with minor fouling on the other surface [298]. However, acid–ultrasound cleaning restored the membrane properties. Glyphosate recovery and HCl/NaOH production were obtained by BMED of alkaline glyphosate (12.8 g/L, with ~175 g/L NaCl) neutralisation liquor from pesticide industry [299,300]. Glyphosate was recovered by 98.2%, and the NaOH solution (~1.45 M with ~96.5% purity) was produced with maximum *η* of 80.8% and minimum *E_spec_* of 2.15 kWh/kg [300]. Prospecting the base reuse for CO_2_ sequestration, the overall balance estimation was positive only if employing renewable energies.

A three-stage BMED process was developed to remove aniline (1000–3000 ppm) and salt (0.1 M NaCl) from a model wastewater and to simultaneously capture CO_2_, through the reaction of the amine group with carbon dioxide that results in positively charged amine [301]. Aniline was completely transported to the base compartment (at *η* up to 80% and *E_spec_* of ~3 kWh/kg), and the desalination exceeded 94% (at *η* = 90% and *E_spec_* ≈ 1 kWh/kg). The possible use of conventional ED for aniline-H_2_SO_4_ wastewater was suggested by performing an analysis of mass transfer and current–voltage characteristics [302].

Measures for counteracting the adverse effects of leakage currents (parasitic currents flowing through the manifolds) were suggested in a BMED process fed by high ammonium chloride organic wastewater (2.46 M${\;\mathrm{NH}}_{4}^{+}$, 1.95 M Cl^−^, 6 g/L carbocystein) [303]. Experiments and simulations showed that, to enhance the process efficiency and reduce overheating phenomena, a proper stack design should be devised, increasing the relative resistance of the parasitic pathways. In particular, using low-resistance membranes, thin spacers, sufficiently long slots in the spacer gasket, and implementing a two-stage scheme can be fruitful to this aim.

BMED with BM-CEM configuration reclaimed waste solutions with polymeric bonding agents and sorbed heavy metals from, e.g., polymer-enhanced UF [304]. Bonding agents were regenerated (84–95%) in the acid compartment by protonation, while heavy metals (Cu^2+^, Ni^2+^, Co^2+^ and Pb^2+^) were transported to the base compartment and separated by formation of hydroxides.

Spent caustic reclamation for NaOH regeneration via BMED was demonstrated by testing a BM-CEM unit [305]. From a spent caustic with 0.44 M NaOH, 0.29 M Na_2_CO_3_, and 0.048 M Na_2_SO_4_, optimal conditions yielded a 0.11 M base at *η* approaching 100%, *E_spec_* of ~8 kWh/kg NaOH, and an estimated cost of 0.97 US$/kg (process capacity of 18.9 kg/year), without observing effects due to oil. 

ED concentration was reported for HCl from waste effluents originating from hydrolysis of palm oil by-products [306], and for NaOH from cellulose mercerisation wastewater [307].

ED technologies have been widely studied for production of organic acids, with development for some industrial applications [19,308]. New opportunities are derived from the recovery of organic acids from wastewaters via ED [309,310,311,312,313,314,315], or BMED [316,317,318] or both [319,320], including systems combined with biotechnologies.

**Figure 18:**
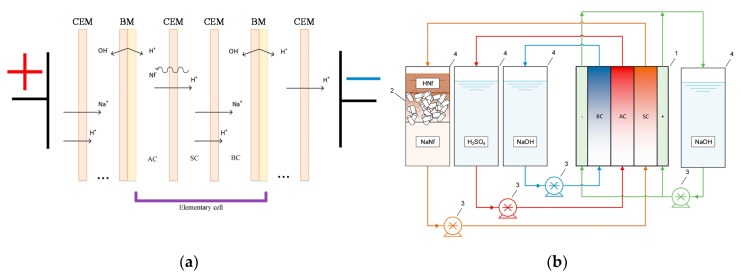
BMED recovering naphthenic acids from sodium naphtenates: (**a**) BM-CEM-CEM configuration; (**b**) flow scheme with electrodialyzer (1), Raschig rings (2), pumps (3) and tanks (4). Reproduced (adapted) with permission from [321], published by Elsevier, 2019.

Naphthenic acids were recovered by BMED from sodium naphtenate solutions [321]. Naphthenic acids are valuable chemical raw materials, which negatively affect the quality of petroleum distillates. They are removed by alkaline extraction, thus generating a solution with salts of naphthenic acids. The BMED with BM-CEM-CEM three-chamber unit cell formed insoluble naphthenic acids (salt compartment fed with 18 wt% sodium naphtenate, 24 wt% NaOH, 1.5 wt% oil), which are separated in a sodium naphthenate reservoir filled with Raschig rings (Figure 18). The same process was conducted by introducing a cation-exchange resin (EDI) and sodium sulphate in the salt chamber, obtaining (i) a reduction in the electrical resistance, (ii) gains in limiting current density, *η* (up to ~80%) and *E_spec_* (0.38 kWh/L), and (iii) a total conversion.

### 4.3. Desalination

Several industrial processes originate salty wastewater, which needs to be desalinated before its reuse or discharge. To this end, the use of ED has been studied for the following main types of wastewater from industrial activities: produced water from oil and gas extraction (Section 4.3.1), wastewater from refineries and petrochemical industries (Section 4.3.2), drainage wastewaters from coal mining (Section 4.3.3), and wastewater from power plants (Section 4.3.4).

#### 4.3.1. Oil and Gas Extraction

Operations of oil and gas extraction produce large volumes of effluents. The amount of water produced worldwide as a by-product of oil and gas production is ~250 × 10^6^ barrels/day, i.e., approximatively triple the produced oil [322]. During some extractions of oil and gas, water can be brought to the surface, as present in (or nearby) the hydrocarbons reservoir, or because intentionally injected with additives to enable the withdrawal. Water is pumped during extraction of unconventional gases, such as coal seam gas and shale gas. The former (known also as coal bed methane), which is adsorbed to the coal surface, is released up to ~1 km underground by effect of a depressurisation applied by wells pumping water from the seams. The latter is extracted from greater depths via hydraulic fracturing, i.e., rocks fracturing by injecting a high-pressure liquid (water with chemicals) from vertical or horizontal wells, thus producing flow-back water. The produced water is also generated during oil recovery enhanced by the polymer flooding technique, which consists of injecting a high-viscosity aqueous solution with soluble polymers that improves the oil sweep efficiency by a less mobile phase.

The produced water composition is strongly site-specific. Total Dissolved Solids (TDS) may amount from some to ~300,000 mg/L [322], being 200–40,000 mg/L in coal seam gas produced water [323] and more in shale gas produced water [324,325]. Polymer flooding produced water falls in the lower part of this range. Additionally, produced waters contain oil, organic compounds, suspended solids, heavy metals and natural radioactive materials. Therefore, treatments (e.g., biological and physico-chemical processes) are needed before reuse/discharge. Membrane processes [322,323,324,325,326,327], including ED for desalination, can be adopted.

Two ED applications are documented in [328]. After de-oiling and removal of dissolved organics (floatation and fluidized bed reactors), the produced water from a conventional well was desalinated, attaining a TDS removal of ~89% from 9100 ppm. Coalbed methane produced water (TDS up to 27,000 ppm) was recovered by 80–90% by mobile ED units, and was reused for fracturing.

ED experiments with simulated produced waters (TDS from ~4400 mg/L to ~97,600 mg/L through the diluate, 25 g/L NaCl through the concentrate) were carried out in order to evaluate the attainment of standards for different reuses (targets of 500–5000 mg/L TDS) [329]. At low feed concentrations, regardless of the composition, it was possible to attain the concentration targets with *E_spec_* of ~1.2 kWh/m^3^, while at high concentrations it was not feasible due to exaggerated *E_spec_* values (more than 20 times higher) or even to unattainable targets. Several lab-scale tests exhibited promising results, even for hypersaline solutions [330,331,332]. Cost analyses (based on experiments with NaCl solutions) and models showed that ED is a cost-effective method for brackish water [333], but, if optimized, it can be competitive also for high salinity feeds [331]. However, the system behaviour should be characterized by tests with real feeds, where other ions are present.

With simulated produced waters from shale gas fracking (3% or 6% NaCl with 1000 or 4000 mg/L Ca^2+^), scaling at the cathode chamber was mitigated by an MVC end-membrane (Ca^2+^ flux decreased by 47–73%), thus obtaining a current density increase of ~40% [334]. Both simulated and real produced waters from shales were then used [335]. After pre-treatment (NaOH dosage, settlement and MF) and, in some cases, dilution, the field samples were partially desalinated by ED (feeds with 25,000–44,600 mg/L TDS, removals up to ~60%) showing similar performance compared to simulated effluents. Operations with a periodic pulse polarity-reversal enhanced the ion migration by temporarily disrupting the stagnant layer of Ca^2+^, Mg^2+^ and Ba^2+^ retained by the MVC. Nevertheless, the occurrence of some precipitation of Fe(OH)_3_ on any IEM suggested to boost the pre-treatment.

**Figure 19:**
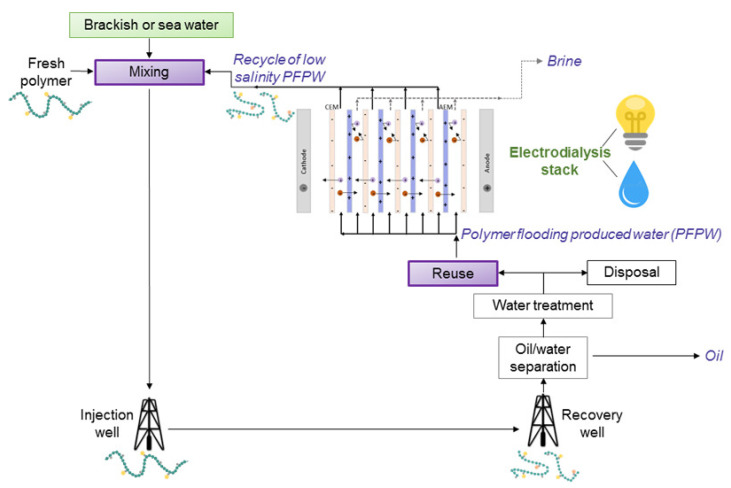
Scheme of ED desalination of polymer flooding produced water. Reproduced from [343], published by Elsevier, 2018.

For reusing polymer flooding produced water, TDS must be reduced at 500–1000 ppm because higher concentrations could lessen the viscosity of the solution. ED desalination can be applied (Figure 19), and has been demonstrated by pilot-/large-scale plants [336,337]. Serious fouling issues occur, and its mechanisms were investigated along with fouled membranes characterisation [338,339,340,341], proposing chemical cleaning strategies [337,342]. Synthetic solutions at 5000 mg/L (brackish water) or 32,000 mg/L (seawater) TDS and with 1.0 g/L partially hydrolysed polyacrylamide (HPAM) were reusable after ED desalination (concentrate feed with 5 g/L NaCl) with small replenishment of polymer (~25% was withhold in the stack) [343]. *η* was 85–99% and *E_spec_* was 0.5–6 kWh/m^3^. Moreover, a preferential removal of divalent ions was feasible, especially at low current densities [344]. Separating multivalent ions is desirable to allow polymer-flooding produced water to be reused, since they have the most significant effects in reducing the solution viscosity (calcium and magnesium) and could lead to scaling and reservoir souring. Tests with different solution compositions showed that fouling issues were associated mostly to HPAM adsorption on AEM and formation of a gel layer favoured by divalent cations [345]. However, the gel layer was significantly removed by application of current reversal and use of foulant-free solution. The minimisation of the gel layer formation was then obtained by applying pulsed electric fields [346]. Oily compounds (synthetic solution with 53.3 mM NaCl plus ${\mathrm{HCO}}_{3}^{-}$, ${\mathrm{SO}}_{4}^{2-}$, Ca^2+^ and Mg^2+^, 250 mg/L HPAM and 2 mg/L crude oil) increased slightly membrane fouling, but made the HPAM gel layer less stable. The best condition (1 s/1 s of pulse/pause) led to a reduction of ~35% in *E_spec_* (~0.6 kWh/m^3^).

One study proposed reverse electrodeionisation (REDI) for simultaneous energy recovery and water reuse by controlled mixing between treated fracturing produced water and fresh water (needed for replenishment) [347]. The REDI diluate contained IXR-wafers to lessen the electrical resistance. The highest *P_d_* was 0.9 W/m^2^ along with a *P_d,net_* of 0.79 W/m^2^ by using produced water at 162 mS/cm (~130 g/L, after NF) coupled with fresh water at 1 g/L, without observing fouling. Assuming the use of 5 × 10^6^ gallons and 60% water recovery from drilling, rough economic calculations show an average increase in revenue over 300,000 $/(year·well). However, the profit will depend strongly on the process (mainly capital) cost. 

#### 4.3.2. Refineries and Petrochemical Industries

Petroleum refineries use water for many processes [348,349]. However, cooling processes are responsible for ~90% of overall water consumption [349]. Refinery wastewaters, including cooling tower blowdown, are sent to treatment plants [350], and ED can be adopted for desalination.

A pilot EDR was installed (late 1980s) at STANIC Industria Petrolifera (Livorno, Italy) [351]. The effluent from the biological treatment (~1500 ppm TDS) was desalinated by ~90%, thus providing cooling tower makeup and boiler feeding, along with a concentrate suitable for discharge, and kicking off the construction of a 180 m^3^/h full-scale plant.

A pilot EDR was used as pre-desalination step followed by RO for a tertiary effluent (~1150 mg/L TDS) from petrochemical industry [352]. Two ED modules (75 cell pairs per each, with a total area of 28.8 m^2^) were used either in series or in parallel mode. The EDR step removed up to ~90% of TDS, while the EDR-RO hybrid system achieved overall removals above 90% for several physico-chemical parameters, with 41% water recovery (75% in EDR, and 50% in RO). Water recovery can be boosted by hybrid schemes in which ED treats the RO brine retentate (see Section 6.1.3).

#### 4.3.3. Coal Mines

Coal mining drainage waters are brines with high salt content that must be reduced to allow water reuse. An industrial EDR application at Tutuka power station (South Africa) was upgraded to a more than doubled capacity (13,200 m^3^/day) to desalinate mine water as well (2500 mg/L TDS) [353] (see Section 4.3.4).

EDR tests with coal mine effluent at low concentration (~2100 mg/L TDS) were performed at single pass with a diluate velocity ten times the concentrate velocity [354]. Despite the high super-saturation level of calcium sulphate and calcium carbonate in the concentrate, crystallisation was avoided by the insufficient residence time, thus preventing scaling. Water recovery of ~90% and salts removal of ~70% were obtained. ED brine valorisation by salt production via two-stage ED (prior to evaporation-crystallisation) was studied with a coal mine solution with 32.8 g/L Cl^−^, finding *E_spec_* values in the order of 10 kWh/m^3^, and producing a sufficiently pure concentrate [355]. Then, a combined NF-ED-RO system was proposed [356], supported by additional experiments [357]. An alternative way of coal mine brine valorisation could be represented by energy recovery through RED [358]. Artificial solutions simulating coal mine brine (111 g/L NaCl) and fresh water (0.56 g/L NaCl) produced a *P_d,max_* of 0.87 W/m^2^ and a corresponding *P_d,max,net_* of 0.71 W/m^2^. An investment cost of 3 $/kWh was estimated by assuming a peak power of ~1 W/m^2^ (low-resistance membranes), showing that the economic feasibility is strongly dependent on the membrane cost.

Sulfide minerals (pyrite, FeS_2_) can be oxidized when in contact with water and oxygen, thus resulting in acid mine drainage that contains sulphate, iron and other (heavy) metals. Samples collected from different locations in a carboniferous, with pH < 3 in most cases and different compositions (conductivity from 1155 to 15,300 μS/cm, ${\mathrm{SO}}_{4}^{2-}$ from ~500 to ~8000 mg/L, various cations) were desalinated by ED (after settling and MF) achieving removals of 97–99%, thus recovering the diluate [359]. Iron precipitation was observed on CEMs, thus long-term operations could require pre-treatment to prevent scaling. A reduction in membrane resistance was found at higher current densities with solutions of Fe_2_(SO_4_)_3_, attributing this behaviour to the ${\mathrm{FeSO}}_{4}^{+}$ dissociation into more mobile Fe^3+^ and ${\mathrm{SO}}_{4}^{2-}$ ions at the boundary layer [360]. Moreover, a significantly different selectivity was observed between homogeneous and heterogeneous CEMs immersed in mixtures with Na_2_SO_4_.

#### 4.3.4. Power Plants

Power plants’ cooling tower blowdown can be desalinated by ED. The industrial EDR with 7-year operation at Tutuka power station cited in Section 4.3.3 was accomplished in ZLD approach [353]. The plant, upgraded (13,200 m^3^/day) also to treat mine water, received a feed with 2500 mg/L TDS with ~50% CaSO_4_ saturation. After pre-treatment with HCl dosage for scaling inhibition, chlorine dosage against organics, and coagulation–filtration for suspended solids removal, the EDR plant recovered water by 75% at *η* of 86%, with attractive costs and long membrane life.

Cooling tower blowdown (conductivity from 2.3 to 3.5 mS/cm, flow rate of 2.3 m^3^/h) was treated by including EDR desalination in the pilot facility (lamella separator, UF, MF, EDR) in Terneuzen, The Netherlands [361]. The ED stack comprised four hydraulic stages and two electrical stages. Normalized parameters were introduced for pressure drop, IEM resistance and *η* to control, monitor and optimize the process. In a 2-month operation, *η* was stable.

### 4.4. Treatment of Other Wastewaters

This section reports studies on ED methods (for separation, desalination, concentration, regeneration or energy recovery) applied to other industrial wastewaters that have not been presented above. Table 2 and Table 3 regard effluents with and without organic matter, respectively. 

## 5. Municipal Wastewater and Other Effluents

Desalination via ED can make treated municipal wastewater reusable, as shown by several field plant applications (Section 5.1). As an alternative, it could be used as a low-salinity solution coupled with seawater for recovering salinity gradient energy (Section 5.2). Additionally, ED methods are under study to recover nutrients (as well as water) and, in some cases, volatile fatty acids (VFAs) from treated wastewater and related/similar effluents (Section 5.3). Another ED application studied is the regeneration of liquid desiccant solutions for air conditioning (Section 5.4).

In addition, ED methods have been proposed for desalinating drainage wastewaters for agricultural reuse [408], and the experimental screening/optimisation has been studied [409].

### 5.1. Desalination of Municipal WWTP Effluents

Secondary or even tertiary effluents from wastewater treatment plants (WWTPs) are normally not reusable in irrigation, aquifer recharge, or industrial processes. When the salinity of treated effluents is relatively high, it can be suitably reduced by ED. MF is often used before ED to remove suspended solids and microorganisms. Pre-treatments, EDR operation and cleaning procedures against fouling can maintain or restore, at least partially, IEMs properties.

A rapid sand filtration-activated carbon-EDR plant of ~1100 m^3^/day capacity supplied treated wastewater to Moody Gardens plants and fishponds (Galveston, Texas) [410]. Other EDR plants (Middle East) were cited in the same paper. In Gran Canaria, a pilot MF-EDR plant was tested for irrigation water reuse [411]. The EDR achieved reductions of conductivity and TDS of 89% and 74%, respectively, from a feed with 2.7 mS/cm average conductivity and 1565 mg/L average TDS. Among the other physico-chemical parameters, the following removals were obtained: 79% ammonia, 88% nitrate, 59% phosphate, 83% BOD_5_, 40% COD, 50% faecal coliforms, from feed concentrations of 24 mg/L, 50 mg/L, 56 mg/L, 18 mg/L, 65 mg/L, 4 colonies/100 mL, respectively. The chance of slightly reducing capital costs compared to RO (by ~6%) was shown.

In another plant with UF and RO for irrigation reuse (Las Palmas, Gran Canaria), the pilot EDR produced 100–140 m^3^/day of desalted water (<500 mg/L) with 82–90% recovery [412]. Metal membrane MF and ED provided a stable effluent quality over a 6-month testing, reducing by more than 90% most physico-chemical parameters, including nutrients [413]. A pilot plant with 144 m^3^/day capacity consisted of 500 μm pre-filtration, coagulation-disinfection (Fe_2_(SO_4_)_3_ and NaClO), 15 μm multimedia filtration and EDR [414]. EDR performed a desalination of ~70% (1104 mg/L TDS in the EDR feed), thus providing an effluent for horticultural reuse with TDS below the 375 mg/L target identified by guidelines. *E_spec_* was 1 kWh/m^3^ (60% of which was in EDR) and 82% of wastewater was recovered with an estimated operating cost of 18 $cents/m^3^. A further benefit from ED is the hypochlorite production in the anolyte, to be used then for disinfection [415]. Pilot MF-RO and MF-EDR plants were compared for a tertiary effluent containing endocrine disrupting chemicals, and pharmaceuticals and personal care products, showing that only RO was capable of removing them, as expected [416]. The same conclusion was drawn from another study, which exhibited moderate removals of some compounds (48–58%) and low removals for other compounds [417].

ED desalination was tested for 930 h during one year with a macrophytes pilot system effluent [418]. The conductivity reduced from ~0.67 mS/cm to 0.2 mS/cm, obtaining an effluent appropriate for reuse (e.g., in cooling towers) with only slight fouling. Further tests were conducted with the secondary effluent from a small WWTP for a university campus sewage [419]. The conductivity of ~1 mS/cm reduced to 0.05–0.1 mS/cm, with removals above 80% for cations and 70% for anions, and with *E_spec_* values of 0.104 kWh/m^3^ increased by fouling to 0.119 kWh/m^3^. Other physico-chemical features (colour, turbidity, COD, BOD, etc.) were mildly cut down, thus providing an effluent suitable for fish farming after simple pH correction, but requiring some further treatment to reduce BOD and turbidity in case of urban and agricultural reuse.

**Figure 20:**
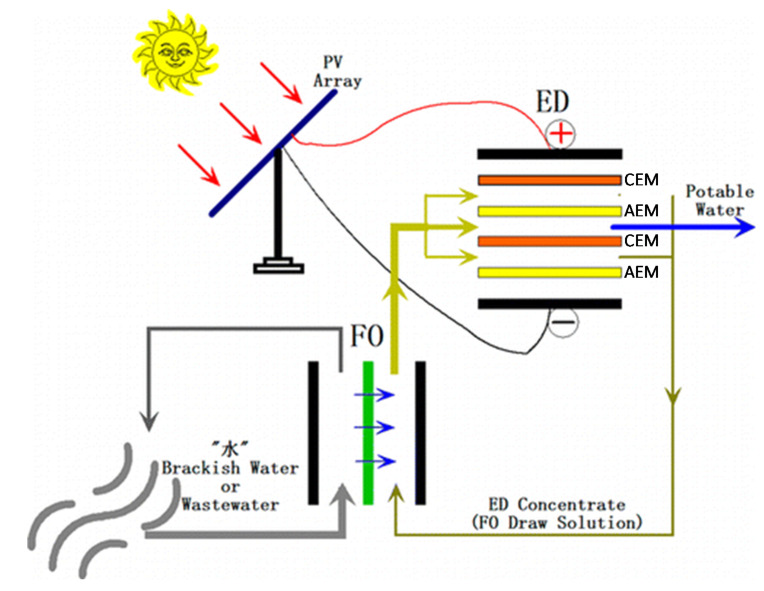
Scheme of FO–ED integrated process for wastewater (or brackish water) reclamation. Reproduced (adapted) with permission from [420], published by American Chemical Society, 2013.

Different schemes coupled ED and forward osmosis (FO). For example, FO extracted water from a secondary effluent (0.05 M salt concentration) to provide it to a draw NaCl solution then sent to the ED stage [420] (Figure 20). Ions, organic and inorganic substances were rejected in the FO retentate, while the draw stream enriched in water (diluted from 0.5 M to 0.2 M) by osmosis went to the ED. This yielded high-quality water (0.81–0.88 mS/cm) and the draw solution for the FO. ED driven by photovoltaic energy exhibited *E_spec_* of 4.98–5.57 kWh/m^3^ and *η* of 78.2–100%, while the estimated cost for a system producing 130 L/day potable water was ~3–5 €/m^3^.

An osmotic membrane bioreactor–ED system treated a synthetic primary effluent (300 mg/L COD, 0.51 g/L salts, 1.1 mS/cm) [421]. FO and biological degradation by activated sludge occur in the bioreactor, which thus suffers from salt accumulation caused by osmosis to the draw side and contrary solute flux. This would result in increased costs for draw replenishment, as well as in discharge issues and microbial growth inhibition. In this study, ED desalted the treated wastewater, by achieving a salinity build-up mitigation (conductivity maintained at 8 mS/cm), which allowed for (i) an increase by 6 times of the biological treatment duration (24 days), and (ii) the waste salt recovery, thus providing the concentrate draw solution. *η* was 41.6–76.2% and *E_spec_* 1.88−4.01 kWh/m^3^. In another hybrid process, ED mitigated the salinity build-up in the FO (submerged module) feed (secondary wastewater with 29.3 mg/L COD, ~0.5 mS/cm) by using a fertilizer draw solution with 0.5–2 M (NH_4_)_2_HPO_4_ [422]. A diluted fertilizer was recovered by FO from wastewater, while 96.6% of the fertilizer lost by reverse flux (63 mg/L ammonium and 83 mg/L phosphate) to the feed was recovered through ED, which also returned the desalinated feed to the FO module. *E_spec_* of the system was 0.72–1.49 kWh/m^3^.

### 5.2. Energy Recovery

WWTP effluents can be used as diluate coupled with salty waters as concentrate in RED stacks recovering energy. The most abundant high-salinity solution is represented by seawater, thereby implying possible applications in coastal areas.

RED experiments with artificial NaCl solutions (diluate 0.002–0.08 M, concentrate 0.6 M) showed that the optimal diluate concentration maximizing the power output (*P_d,max_* of 0.39 W/m^2^) was in the range 0.01–0.02 M [423], which often corresponds to the concentration range of WWTP effluents from biological treatment. By increasing the temperature from 25 °C to 60 °C, *P_d,max_* increased by 60%, suggesting that co-locating RED with a thermal power plant where the solutions are pre-heated (e.g., cooling tower seawater) can boost the energy recovery. Moreover, the seawater–WWTP effluent RED process may be used as a pre-desalination step before RO, by providing great potential of reducing the energy consumption of seawater desalination plants [424]. The RED unit may also be operated under “assisted” conditions, in which the applied electrical current overcomes the short-circuit current, thus requiring a lower membrane area [425]. Simulation results showed that hybrid RED-RO systems either with assisted or conventional RED yielded cost savings compared to standalone SWRO [426]. However, a critical issue affecting these RED-RO systems is the potential contamination of the seawater by organic micropollutants that may be adsorbed and transported from the impaired water [427]. In contrast, this problem is less important in RO-RED schemes (Section 6.3), where the RED process is fed by the RO reject brine and the WWTP effluent.

Several studies tested RED with real WWTP effluents and seawater. After filtration by a 10 μm filter, a treated wastewater at conductivity of 0.44 mS/cm (~0.002 M) coupled with seawater (46.2 mS/cm) produced a *P_d,max_* of 0.15 W/m^2^ [398]. The low concentration of the diluate led to a high electromotive force (*V_OC_* of 1.66 V with 10 cell pairs, 70% permselectivity), but limited the power density due to the high electrical resistance. The presence of natural organic matter (NOM) in the WWTP effluent (16.3 mg/L dissolved organic carbon) increased the resistance, causing a reduction in *P_d,max_* of ~17% with respect to a model solution lacking NOM.

Higher values of *P_d,max_*, i.e., up to 0.38 W/m^2^, were obtained by testing a pilot plant [428]. The WWTP effluent was treated by dual-media filtration, bag filter (50 μm) and cartridge filters (5 μm), while the seawater underwent only the 5 μm filtration. The RED feed solutions had conductivity of 1.3–5.7 mS/cm and 52.9–53.8 mS/cm, respectively. The transport of inorganic solutes and NOM (4.3 mg/L dissolved organic carbon in the treated wastewater) was investigated. Over 12 days, the power density was on average ~20% lower than the highest one. It was observed a slight increase of the IEMs resistance, which can be attributed to fouling and effects of divalent ions. However, the reduction of *P_d_* was mainly caused by precipitates clogging the cathode chamber, where the wastewater was used as electrolyte (high pH due to the hydrogen evolution reaction). Organics at low molecular weight were transported towards the seawater compartments. Therefore, attention should be paid to this aspect in case RED is followed by RO. Pressure drops increased continuously over 12 days up to almost 3 and 1.5 times in the wastewater and seawater compartment, respectively, due to spacer-filled channels clogging at the inlet regions. This may affect significantly the net power (not calculated). Therefore, suitable pre-treatments and cleaning procedures should be adopted.

The same *P_d,max_* (~0.38 W/m^2^) was recorded by another pilot RED fed with treated water (anaerobic-oxix activated sludge process) at conductivity of 1.0–2.5 mS/cm and seawater at 50 mS/cm [429]. Actually, the seawater solution was obtained by mixing a desalination brine with the treated water. Suspended particles were removed from both streams by cartridge filter and fibre filter (10 μm pore size). *V_OC_* was 28.6 V (200 cell pairs), 20% lower than the theoretical one due to effects of divalent ions and reduction of driving force along the channels. A 5 wt% Na_2_SO_4_ electrolyte was fed to the electrode compartments, obtaining 0.9 L/h H_2_ with ~100% efficiency by cathode reduction. Without pre-filtration, the performance was stable over 300 h, but a subsequent reduction of power density was observed (up to 80% after 800 h). Cleaning without chemicals removed clogging from the disassembled stack and restored its performance. However, cleaning in place methods should be developed with short interruptions of the RED process.

Filtration pre-treatments of a domestic WWTP effluent (1.13 mS/cm) were compared, i.e., 100 μm filtration, rapid sand filtration or river bank filtration [430]. Seawater (48.35–58.38 mS/cm) was pre-treated with sand filtration, bead filtration and UV. During 40-day RED testing, the pressure drop increased by only 0.09–0.18 bar from the initial value of 0.03–0.04 bar when using 100 μm filtration and rapid sand filtration, respectively. With an almost stable *P_d,max_* of ~0.25 W/m^2^, *P_d,max,net_* was 0.23 and 0.22 W/m^2^, respectively. Instead, the RED operation with river bank filtration or without pre-treatment exhibited high pressure drops (~0.6 bar on average) and low *P_d,max,net_* (~0.06 W/m^2^), despite alkaline and acidic cleanings. Inlet and outlet regions of spacer-filled channels were critical points of biofilm development even at the seawater side (biological growth during storage).

The pre-treatment with polyaluminium chloride coagulant (and 0.45 μm filtration) was tested for reclaimed water (~0.5 mS/cm) [431]. Filtered seawater (~48.5 mS/cm) was the RED concentrate. Polyaluminium chloride residue affected the RED performance by increasing the CEM resistance and, thus, by reducing the power density. However, the optimized dosage removed up to 50% of the organic matter (from ~6.5 ppm TOC) and resulted in a *P_d,max_* of ~0.42 W/m^2^, increased by 20% with respect to that obtained with filtration only. Multivalent ions and NOM, instead, reduced *P_d,max_* by ~20% with respect to that produced by model solutions. The long-term operation should be tested.

A secondary effluent was treated by coagulation-flocculation, decantation and 10 μm filtration [432]. The treated stream (1.8 mS/cm, ~0.008 M NaCl) was used with 1 μm-filtered and UV-disinfected seawater (54.7 mS/cm, ~0.5 M NaCl) for RED energy recovery, showing a stable performance over 480 h. The treatment before RED and the slight increase of salinity after RED provided a water quality acceptable for reuse. *V_OC_* was 3.45 V (20 cell pairs) and *P_d,max_* was 1.43 W/m^2^ without observed fouling. This is the highest value of *P_d_* recorded so far with this kind of streams. However, no data on pressure drop nor on *P_d,net_* were provided.

RED was performed with both compartments fed by treated wastewater [433]. Similarly to the previous cases, the low-salinity solution was a reclaimed urban WWTP effluent (from membrane bio-reactor pilot plant) at conductivity of 0.6–1.8 mS/cm (corresponding to ~0.004–0.016 M NaCl). Instead of seawater, the concentrate stream was a fish canning factory wastewater, treated by a pilot aerobic granular sludge sequence batch air-lift reactor, 5 μm MF, and acidification at 4 < pH < 5, which had a conductivity of 47.0 or 87.5 mS/cm. During long-term experiments of 29 days, fouling and increase of pressure drops (clogging) were observed. Backwashing with short pulses (1–2 s) at high fluid velocity (10 cm/s) was performed. An alkaline solution through the compartment of the fish wastewater was more effective than other solutions, while distilled water through the compartment of the reclaimed WWTP effluent was sufficient. Periodic ED pulses reduced the absorption of foulants, maintaining almost constant the stack resistance. However, fouling issues did not vanish, especially those involving AEMs. Under the best conditions tested, *V_OC_* was almost constant around 1.6 V (10 cell pairs), while *P_d,max_* declined from ~0.9 to ~0.6 W/m^2^. *P_d,max,net_* corrected by excluding the effect of the blank resistance declined from ~1.3 to 0.1 W/m^2^. Therefore, measures against fouling and clogging should be further studied. Some detrimental effects of divalent ions were observed.

The integration between membrane distillation (MD) and RED was proposed to recover water and energy from urine in off-grid applications [434]. From real urine feed (12.65 mS/cm, 207 mg/L ${\mathrm{NH}}_{4}^{+}-N$, 6.33 g/L COD), the MD produced a retentate with doubled conductivity (24.1 mS/cm) and a permeate at 0.21 mS/cm. These streams were then used as feeds for an RED unit for partial remixing with energy recovery. *P_d,max_* (~0.2 W/m^2^) was comparable to that produced by NaCl solutions (0.32 W/m^2^). By increasing the temperature from 22 to 50 °C, *P_d,max_* could be increased by 70%, as shown by experiments with synthetic solutions, thus prospecting the use of waste heat. In RED tests with recirculation, ~47% of the Gibbs free energy was extracted. In optimized systems, the energy efficiency could be enhanced even compatibly with a good quality of the final diluate.

### 5.3. Recovery of Nutrients and VFAs

ED methods can recover nutrients from wastewater, thus lowering the ecological impact of discharge (eutrophication) and producing fertilizers. In some cases, VFAs are other valuable components that can be recovered along with nutrients. Studies have been performed on treated municipal wastewater (Section 5.3.1), excess sludge sidestreams (Section 5.3.2), separately collected human urine (Section 5.3.3), and waste effluents from animal farming (Section 5.3.4). 

#### 5.3.1. Municipal WWTP Effluents

Municipal WWTP effluents can provide nutrients, i.e., ammonia, phosphate, nitrate, and potassium, thus producing fertilizers. To this aim, ED systems can concentrate P-based nutrients before precipitation/crystallisation of struvite, i.e., (NH_4_)MgPO_4_·6(H_2_O), or calcium phosphates [435].

**Figure 21:**
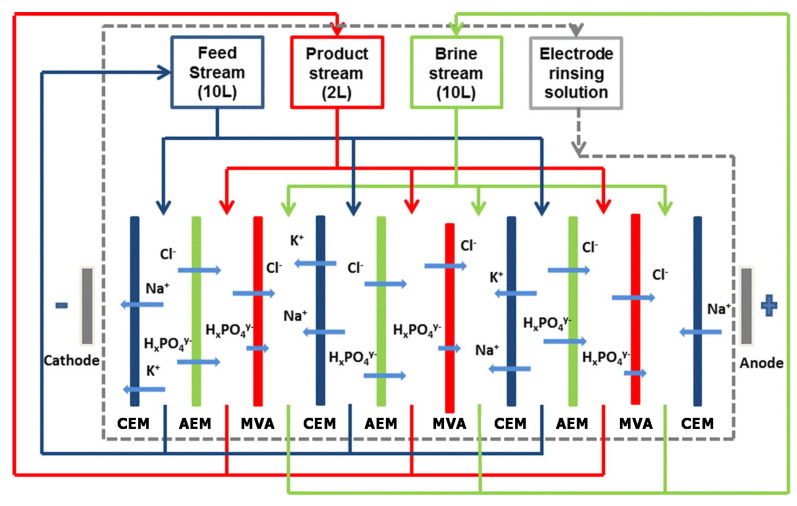
SED stack equipped with MVAs to fractionate and concentrate phosphate. Reproduced (adapted) with permission from [437], published by Elsevier, 2015.

Several studies have tested SED units with MVAs to fractionate and concentrate phosphate (Figure 21). Experiments with synthetic wastewater (3–7 mM KH_2_PO_4_ and 13–17 mM NaCl) achieved a phosphate removal of 62.3% with a product concentration of 16 mM at 44% purity, and a Cl^−^ removal of 87% [436]. Values of *η* for $H_{2}{\mathrm{PO}}_{4}^{-}$ through the AEM and for Cl^−^ through the MVA were 26.6% and 63%, respectively. These outcomes were obtained at pH = 12 in the product, as multivalent phosphates (${\mathrm{HPO}}_{4}^{2-}$ and ${\mathrm{PO}}_{4}^{3-}$) predominate under alkaline conditions. Crystallisation with CaCl_2_ in a pellet reactor produced hydroxyapatite and brushite with 82.7% efficiency, thus demonstrating the feasibility of the integrated process. 

With a more realistic synthetic municipal effluent containing an ion mixture (1 mM KH_2_PO_4_, and 2 mM ${\mathrm{NO}}_{3}^{-}$, ${\mathrm{HCO}}_{3}^{-}$, ${\mathrm{SO}}_{4}^{2-}$, Ca^2+^ and Mg^2+^), although the process required a longer operation, 41.9% of phosphate was removed and concentrated by 161% in the product, and acid cleaning removed scaling [437]. Other experiments with a synthetic secondary effluent (NaCl, NaNO_3_ and Na_2_HPO_4_ with 355 mg/L Cl^−^, 30 mg/L N, 10 mg/L P) exhibited recovery efficiencies of 56.97–64.28% for N in the brine and 67.42–73.67% for P in the product, with overall *η* of 56.7–61% and *E_spec_* of 1.63–2.92 kWh/m^3^ [438]. The pH in the tank with the product was adjusted at 10.2–10.5. However, at high values of applied voltage, lower concentration rates of P suggested water dissociation as a likely cause of pH reduction.

Even conventional ED is able to recuperate nutrients [439]. To accomplish a selective separation, a two-stage underlimiting/overlimiting ED was proposed [440]. A model macrophyte-treated wastewater (0.022 g/L Na_2_HPO_4_·7H_2_O, 0.011 g/L NaH_2_PO_4_·H_2_O, 0.481 g/L Na_2_SO_4_) was circulated through both compartments. In the first stage, ions were concentrated by cycles where the diluate was changed once reached 50% desalination (49.8% Na^+^, 46.7% $H_{x}{\mathrm{PO}}_{4}^{3-x}$, 42.6% ${\mathrm{SO}}_{4}^{2-}$) to avoid pH reduction that would occur at higher desalination percentages. $H_{x}{\mathrm{PO}}_{4}^{3-x}$ reached 0.118 g/L (concentration factor of ~10), which is satisfactory for an efficient precipitation/crystallisation. To segregate phosphate, a concentrated solution was treated with a second stage at overlimiting currents promoting water dissociation and phosphate protonation-deprotonation. Na^+^ and ${\mathrm{SO}}_{4}^{2-}$ were removed by 97.7% and 94.2%, respectively, while phosphate transfer was significantly hampered, by retaining it by 81.3% in the diluate. Studies for membrane characterisation and transport mechanisms elucidation can help to enhance such ED applications [441,442].

A pilot ED was assembled with Mg anode to provide Mg^2+^ to the concentrate and precipitate struvite [443]. Synthetic wastewater with 34.6 mg/L ${\mathrm{NH}}_{4}^{+}-N$, 10 mg/L ${\mathrm{PO}}_{4}^{3-}-P$ and 300 mg/L NaCl was used as initial solution for concentrate, anode and diluate. The concentrate chambers were connected with the anode, so that the product solution exiting the concentrate flowed through the anode and vice versa. At the optimal pH of 8.8 and with multiple cycles in the diluate, 65% of phosphate was removed as struvite, the diluate had on average less than 4 mg/L ${\mathrm{PO}}_{4}^{3-}-P$ (below 0.5 mg/L at the end of several cycles), and the concentrate had 30 mg/L ${\mathrm{PO}}_{4}^{3-}-P$. The cost of the Mg anode was 31.27 $/kg P.

#### 5.3.2. Excess Sludge Sidestreams

ED techniques were proposed also for recovery of fertilizers from excess sludge sidestreams (supernatant, centrate, filtrate) of municipal WWTPs. An economic analysis based on an ED simulator estimated a total cost of 0.392 $/kg N (29.5 m^3^/day capacity), 65% of which due to operation cost (*E_spec_* of 2.36 kWh/kg), showing the convenience of ED compared to other conventional or novel processes [444]. As Table 3 reports, either ED or ED-BMED recovered phosphate or phosphoric acid from a synthetic solution modelling the supernatant of excess sludge mixed with the influent [406], and these treatment processes may be intended also for municipal effluents. In other experiments, an integrated system with ED, struvite precipitation and ammonia stripping was developed by testing synthetic sludge anaerobic digestion sidestreams (dewatering by, e.g., centrifuge or belt filter press) [445]. The feed contained 200 mg/L P and 600 mg/L N. After concentration via ED, nutrients were recovered by the struvite reactor, while the ammonia excess was recovered via gas stripping at 40 °C. Overall removal percentages were ~86% for P and ~92% for N.

**Figure 22:**
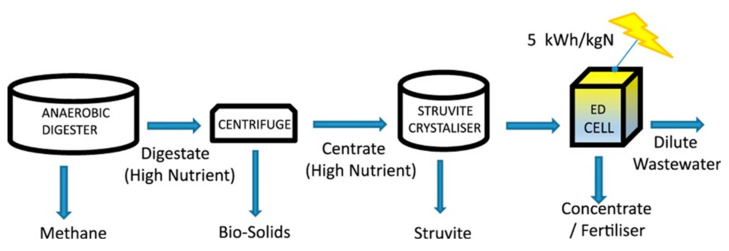
Conceptual scheme of the recovery of materials from anaerobic digestion of WWTP excess sludge, including ED concentration of nutrients from centrate after struvite precipitation for fertilizer production. Reproduced (adapted) with permission from [447], published by Elsevier, 2018.

Lab-scale [446] and pilot-scale [447] ED experiments were conducted with a real anaerobic digester supernatant (centrifuge centrate) to concentrate ${\mathrm{NH}}_{4}^{+}$ and K^+^. A crystallisation/precipitation pre-treatment was performed to recover struvite and prevent scaling (Figure 22). The ED feed (232 mg/L K^+^, 1003 mg/L Na^+^, 768 mg/L Cl^−^, 835 mg/L ${\mathrm{NH}}_{4}^{+}-N$, etc., 351 mg/L COD) was pumped with single pass through the diluate (23% reduction of conductivity) and with recirculation through the concentrate, reaching concentration factors of ~8 for ${\mathrm{NH}}_{4}^{+}-N$ and K^+^ with average overall *η* of 76% and *E_spec_* of 4.9 kWh/kg ${\mathrm{NH}}_{4}^{+}-N$ [447]. The treatment was competitive and provided a product usable as fertilizer. However, improvements were needed, especially in terms of increase of product recovery (affected by water flux and ions back diffusion) and elimination of unwanted ions like Cl^−^. The transport of pharmaceuticals (10 or 100 μg/L) was then studied [448]. Nutrients were concentrated by a maximum factor of ~5, while less than 8% of pharmaceuticals were transported to the concentrate product. However, the lower concentrations usually present in real effluents do not hinder the product use as fertilizer.

A solution prepared with 6.6 g/L NH_4_HCO_3_ (1.5 g/L ${\mathrm{NH}}_{4}^{+}$), simulating sludge reject water, was concentrated by ED with dynamic current [449]. The current density was controlled during the batch process by applying values equal to a fraction of the instantaneous limiting current density (related to the diluate electrical conductivity). This reduced the operational run time by 75% compared to the operation with fixed current, thanks to reduced effects of osmosis and back-diffusion. A more efficient separation was thus obtained: the concentration factor increased from 4.5 to 6.7, while *E_spec_* remained unchanged at 5.4 MJ/kg N removing 90% of ${\mathrm{NH}}_{4}^{+}$ at *η =* 83–96%.

Dissolved ammonia (1.7 or 4.0 g/L) in anaerobic digestion centrate solutions was converted by acid stripping through a liquid–liquid hollow fibre membrane contactor into ammonium salts (NH_4_NO_3_ or NH_4_H_2_PO_4_ at 5.1 wt% or 10.1 wt% in nitrogen), which were then concentrated by ED [450]. Under optimal conditions (10.1 wt%), the ED produced a liquid fertilizer at 15.6 wt% NH_4_NO_3_-N, with *E_spec_* of 0.21 kWh/kg and *η* of ~93%.

A treatment integrating BMED, struvite precipitation, and multi-stage membrane capacitive deionisation (MCDI) was demonstrated for recovering phosphorus and ammonium from a synthetic supernatant (12.5 mM ${\mathrm{NH}}_{4}^{+}$ and 2.5 mM ${\mathrm{PO}}_{4}^{3-}$) [451]. BMED was used only to alkalify the wastewater, thus facilitating struvite precipitation, while MCDI was employed to separate excess ammonia in a small volume. *E_spec_* of the BMED was ~1 kWh/m^3^. The integrated process removed ~100% of phosphorous and ~77% of ammonia, recovering ~81% of high-quality effluent and ~19% of concentrated stream meeting reuse standards.

VFAs are other products extractable from excess sludge. To recover them, as well as nutrients, thermally hydrolysed waste activated sludge was fermented anaerobically, and after screening and MF, the permeate was concentrated by ED [452]. The 6.15 g/L total VFAs present in the MF permeate were concentrated by ED to 19.82 g/L, corresponding to 92% of transferred mass, while 0.92 g/L ${\mathrm{NH}}_{4}^{+}$ and 0.16 g/L ${\mathrm{PO}}_{4}^{3-}$ were concentrated to 3.02 and 0.45 g/L, respectively. After that, struvite precipitation was performed to remove the excess ammonium and phosphate, and fermentation was conducted to produce polyhydroxyalkanoates. ED and precipitation significantly enhanced their accumulation in the fermentation broth (from 24 mg/L to 165 mg/L), thus offering a cost-effective valorisation process.

ED techniques can also recover lactic acid from the organic fraction of municipal solid waste hydrolysate [453].

#### 5.3.3. Human Urine

Human urine comprises up to 91–96% water, with urea (CO(NH_2_)_2_) being the major solute fraction (50% of TOC) and with other organic and inorganic components, including P and K [454]. Anthropogenic urine represents a small fraction of domestic wastewater. Nevertheless, nutrients are present in it, along with micropollutants (endocrine disrupting compounds and pharmaceuticals). Therefore, suitable treatment processes have been proposed for separately collected urine to separate and concentrate nutrients (fertilizers) from micropollutants, including ED [455]. IEMs enable ion passage, while retaining neutral organics, proteins and microorganisms. Therefore, ED produces a nutrient-enriched concentrate stream (starting from water with or without salt), and a waste urine stream (diluate).

After MF, in experiments with lab-scale [456] and pilot-scale [457] ED units, maximum concentration factors of nutrients were 3.3 and 4.1, respectively, with urine desalination of 85–99%. The urine contained 4.85 g/L ${\mathrm{NH}}_{4}^{+}-N$, 0.23 g/L ${\;\mathrm{PO}}_{4}^{3-}-P$, 1.96 g/L Na^+^, 3.83 g/L Cl^−^, 1.72 g/L K^+^, 0.67 g/L ${\mathrm{SO}}_{4}^{2-}$, and 4.36 g/L COD in the former study, while it contained 2.9 g/L ${\mathrm{NH}}_{4}^{+}-N$, 0.18 g/L ${\;\mathrm{PO}}_{4}^{3-}-P$, 1.6 g/L Na^+^, 3.0 g/L Cl^−^, 1.4 g/L K^+^, 0.7 g/L ${\mathrm{SO}}_{4}^{2-}$, and 3.6 g/L COD in the latter study. The compartments of concentrate were filled at the start of the experiment; then, the concentrate flow was generated only by water transport. *η* values up to 50% were observed for ions, suggesting that part of the current was transferred by charged fractions of COD [456]. During a 90-day operation, spiked micropollutants (mixture of pharmaceuticals and hormones) were adsorbed and, after saturation, partially permeated the membranes, reaching the concentrate [456]. However, natural levels of micropollutants (e.g., ~100 μg/L ibuprofen) in the feed would allow much longer operations (e.g., 400 days) without permeation [457]. Membranes cleaned after 195 operating days exhibited a desalination rate enhancement of 35%. Ozonation removed completely micropollutants. Fertilisation by the concentrate showed good performances [457]. However, the ED method would be economically feasible only for large-scale plants, while other processes are affordable for developing countries [458].

**Figure 23:**
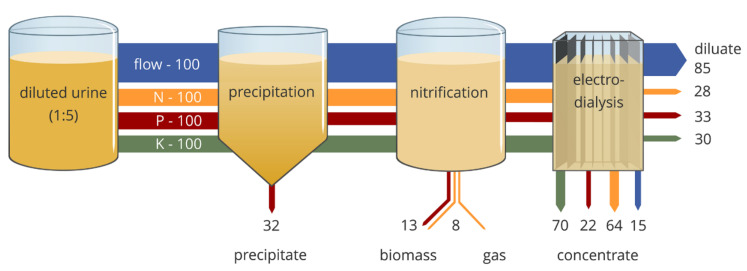
Flowsheet with mass balances of the pilot plant for urine-water solution treatment. Reproduced with permission from [459], published by Elsevier, 2018.

A system with precipitation, anaerobic nitrification and ED concentration was developed and operated for ~7 months [459] (Figure 23). The first two treatment processes minimized scaling and biofouling in the ED step. The influent was with 20% or 40% urine in water (151 mg/L ${\mathrm{NH}}_{4}^{+}-N$, 0.4 mg/L ${\mathrm{NO}}_{3}^{-}-N$, 42 mg/L ${\;\mathrm{PO}}_{4}^{3-}-P$, 449 mg/L Na^+^, 753 g/L Cl^−^, 401 mg/L K^+^, 31 mg/L Ca^2+^, 11 mg/L Mg^2+^, 14 mg/L ${\mathrm{SO}}_{4}^{2-}$ in the 20% solution). After precipitation by NaOH dosage for Ca^2+^ and Mg^2+^ removal, the biological treatment (moving bed biofilm) oxidised organics and stabilised N via ammonification–nitrification, which converts urea (volatile and thermally unstable) into nitrate. The ED transferred 70%/80% of ions in 15%/20% of the starting volume by treating an influent with 20%/40% of urine, respectively (concentration factors of 3–5), with *E_spec_* of 4.3 kWh/m^3^. The P-rich solids from precipitation and the ED concentrate produced fertilizers; the ED diluate could allow water recovery.

#### 5.3.4. Animal Farming

The augmentation of intensive livestock production caused by massive meat consumption implies several environmental impacts, including those associated with inappropriate disposal of raw or digested animal manure (e.g., eutrophication). Animal manure is a slurry which contains faeces, straw, urine, and water, but sometimes the solid fraction is separately collected [460]. The nutrient content (P, K, N) could allow fertilisation by animal manure. Additionally, biogas can be produced via anaerobic digestion [461]. Unfortunately, the direct agricultural use is limited by transportation costs and foul odours release. However, separation and concentration of nutrients from raw or digestate manure can solve these problems, and ED techniques (after solid–liquid separation) have been studied to this aim, especially for effluents from pig farms, which account for ~36% of the meat market [462].

After vacuum filtration, ammonium contained in liquid swine manures (3.29 or 5.14 g/L NH_3_-N, 2.09 or 2.52 g/L K^+^, 0.2 or 0.24 g/L P, 20.66 or 40.32 g/L soluble COD, VFAs, solids, etc.) was concentrated into a 1 g/L KCl feed concentrate, by testing different IEMs [463]. Total ammonia reached ~14.4 g/L (of which free ammonia, NH_3_, represented ~6%) with *η* ≈ 75%. Most of the remaining current was used for K^+^ transport (3.8 concentration factor), while a small quantity of phosphorus was transferred (0.45 concentration factor). Volatilisation of free ammonia caused a 17% loss of total ammonia, whose concentration was also limited by water transport. Coupling RO with ED did not bring improvements. Fouling phenomena impaired the IEMs properties resulting in a decline of ED performance [464]. An alkaline–acidic cleaning was fully successful for the CEMs, but restored 80% of conductivity of AEMs, which were likely affected by permanent organic fouling (dark coloration). After cleaning, current density and desalination rate were 95% and 91% of the values measured with new IEMs. To limit losses of ammonia by volatilisation, its transfer to an acid solution by air stripping of the concentrate was tested [465]. Total ammonia nitrogen was concentrated by ~7 times (from a feed at 3.2 g/L) with *η* = 64–71%. However, the acidic trap recuperated only 14.5% of NH_3_ present in the concentrate. Since the residual total ammonia in the swine manure was 1.2 g/L, the NH_3_ recovery could be enhanced at higher pH values.

Nutrients were recovered by EDR treatment of pig manure digestate [466]. The raw manure was completely digested. Then, pre-treatments prior to ED included: acidification for P extraction from solids, 0.4 mm sieving, flocculation, centrifugation. The generated effluent (2637 mg/L ${\mathrm{NH}}_{4}^{+}-N$, 492 mg/L ${\mathrm{PO}}_{4}^{3-}-P$, 3259 mg/L K^+^, 8894 mg/L Cl^−^, 691 mg/L Na^+^, 1021 mg/L Ca^2+^, 555 mg/L Mg^2+^, 2.8 g/L soluble COD, VFAs, solids, etc.) was the diluate feed. All ammonium and most of phosphate (84%) were removed, obtaining a concentrate product at 4.2 g/L ${\mathrm{NH}}_{4}^{+}-N$ and 0.7 g/L ${\mathrm{PO}}_{4}^{3-}-N$, with overall *η* of 46.8–61.3% and *E_spec_* of 0.13 kWh/L. At every polarity reversal (15 min), acidic cleaning was performed. Reversible fouling was removed, but irreversible effects of organic fouling were observed as well. However, they became stable, likely because the foulants transport inside the membrane was hindered by superficial fouling, thus suggesting the feasibility of long-term operations. The antibiotics fate in the EDR process was studied with spiked (from 50 μg/L to 5 mg/L) pre-treated pig manure, by investigating sorption and migration mechanisms and fouling formation [467]. The main conclusion was that antibiotics can be transported to the concentrate, thus presenting the possible need for further treatment.

The ED stack with Mg anode for struvite precipitation discussed at the end of Section 5.3.1 was also tested with a solution that was 10 times more concentrated (100 mg/L ${\mathrm{PO}}_{4}^{3-}-P$) simulating swine wastewater digestate [443]. The final diluate had on average ~20 mg/L ${\mathrm{PO}}_{4}^{3-}-P$, the concentrate product had ~100 mg/L ${\mathrm{PO}}_{4}^{3-}-P$.

**Figure 24:**
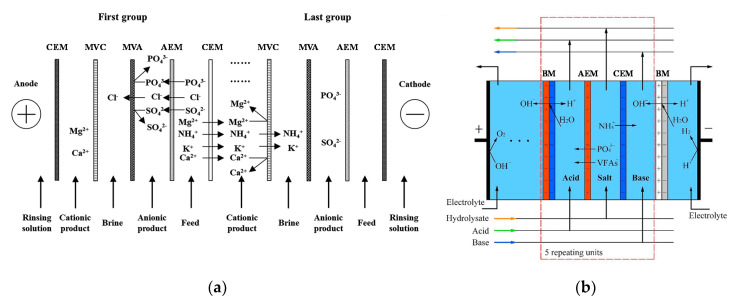
Schematics of ED methods for animal farming effluents: (**a**) four-compartment SED of swine manure digestate; (**b**) BMED of pig manure hydrolysate. Panel (**a**) is reproduced (adapted) with permission from [468] published by Elsevier, 2019. Panel (**b**) is reproduced (adapted) with permission from [469], published by Elsevier, 2018.

The simultaneous fractionation of cations and anions into several streams by SED offers an interesting approach to recover nutrients from digested swine manure [468]. The tested SED stack (Figure 24a) was built with repeating units of four membranes, i.e., AEM, CEM, MVC and MVA, and four channels, i.e., feed, cationic product (Mg^2+^ and Ca^2+^), brine (K^+^ and ${\mathrm{NH}}_{4}^{+}$) and anionic product (${\mathrm{PO}}_{4}^{3-}$ and ${\mathrm{SO}}_{4}^{2-}$). The initial feed was prepared with NaH_2_PO_4_ (40 mg/L P), NH_4_Cl (500 mg/L N) Na_2_SO_4_ (100 mg/L SO_4_), KCl (400 mg/L K), MgCl_2_ (60 mg/L Mg), CaCl_2_ (100 mg/L Ca) and NaCl (3.192 mM). The other initial solutions were with 0.1 M NaCl. The feed conductivity was practically reduced to zero, obtaining fractionations from ~33% (K^+^) to ~90% (${\mathrm{PO}}_{4}^{3-}$), *η* from ~2% (${\mathrm{SO}}_{4}^{2-}$) to ~30% for ${\mathrm{NH}}_{4}^{+}$, and *E_spec_* from ~1 kWh/kg NH_4_Cl to ~0 kWh/kg NaH_2_PO_4_. The two divalent ion products were mixed, obtaining phosphate precipitation by NaOH dosage.

BMED recovered nutrients and VFAs from pig manure hydrolysate (after acidification and solid−liquid separation, the effluent contained ~4.0 g/L ${\mathrm{NH}}_{4}^{+}-N$, ~1.8 g/L ${\mathrm{PO}}_{4}^{3-}-P$, 9.54 g/L VFAs, 52.68 g/L COD, residual solids, etc.) [469]. The salt tank was filled with the effluent, while the acid and base tanks with deionized water (Figure 24b). Preliminary experiments with model solutions exhibited low values of recovery efficiency, *η* and purity, due to ion diffusion. The migration of Cl^−^ and ${\mathrm{SO}}_{4}^{2-}$ (as well as ${\mathrm{NH}}_{4}^{+}$) was faster than other ions and constant until reaching low concentrations in the feed compartment. Instead, ${\mathrm{PO}}_{4}^{3-}$ and acetate remained in the feed compartment until that time, and then started to migrate. Therefore, a two-stage process enhanced significantly the performance. Once reached the inflection point of voltage (galvanostatic mode) corresponding to the start of ${\mathrm{PO}}_{4}^{3-}$ and VFAs transport, the produced acid and base were substituted with demi water. Two acid and two base products with higher purity were thus generated: acid I contained mainly strong acidic ions (~30 g/L Cl^−^), acid II had high concentrations of acetate and ${\mathrm{PO}}_{4}^{3-}$ (~15 and 5 g/L, respectively, with recovery efficiency of 87% and 77%), base I and II contained mainly ${\mathrm{NH}}_{4}^{+}$ (~10.5 and 3.2 g/L, respectively, with recovery efficiency of 60%) with Cl^−^ impurity. Air stripping of base I recovered NH_3_, while acid I acidified the pig manure. P and VFAs may be extracted from acid II by further processes.

### 5.4. Regeneration of Liquid Desiccant Solutions for Air Conditioning

Liquid desiccant air conditioning has emerged as an efficient and energy-saving alternative to vapor compression air conditioners in buildings. In particular, liquid desiccant can dehumidify air by absorption. Membrane processes can replace conventional thermally driven evaporation methods in the regeneration (concentration) of hypersaline liquid desiccant solutions, by avoiding droplet carry-over and by lowering energy consumption [470]. Several studies have addressed the ED regeneration of LiCl or LiBr liquid desiccant solutions.

The application potential and the benefits over thermal regeneration were first shown by theoretical studies, where ED was powered by photovoltaic energy [471]. A two-stage ED was more advantageous, by saving more than 50% of energy compared to a single stage under optimized conditions [472,473]. An experimental setup was then developed, obtaining a maximum difference in LiCl mass concentration in the regenerated desiccant solution between start and end of ED of 0.03 wt% (experiments conducted with initial regenerate concentration of ~21–23 wt%) [474]. Low *η* values were observed (<55%), which is not surprising, given the hard conditions where the IEMs have to work, i.e., high concentrations and high concentration gradient. *η* values spanned over a large range (21–65%) in other experiments, showing the important role played by the concentrate (regenerate) −diluate concentration difference [475]. The same occurred towards *E_spec_* and the overall coefficient of performance of the liquid desiccant air-conditioning system with ED regeneration, COP, defined as the refrigerating capacity divided by the power consumption (*E_spec_* ≈ 1.4–16 kWh/m^3^, COP ≈ 0.3–4). However, at high current densities and concentration differences, *η* and COP were governed by liquid desiccant concentration and flow rate, membrane properties and stack design. Experiments and simulations showed that higher values of concentration difference, initial regenerate concentration and applied current, reduced *η* (20–70%) [476]. However, the concentration difference had lower effects on the COP and energy efficiency. In contrast, the increase of the initial concentration from 27 wt% to 35 wt% reduced *η*, but enhanced the COP from 4 to 6.2 (*E_spec_* ≈ 8.3–8.7 kWh/m^3^).

In other experiments, *η* values between 55.17% and 73.54% were found [477]. With initial concentrations of 23.96 wt% and 28.77 wt% for spent and regenerate solution, respectively, when a concentration difference of 5.86 wt% was reached, a concentration decrease in the regenerate was even observed. Negative effects derived from osmosis and electro-osmosis from diluate to concentrate, and salt back diffusion. Further tests and simulations showed that water transport was more significant than salt transport, and increased as the applied current increased and the initial solution concentration decreased [478]. It was concluded that minimizing the concentration difference between the two solutions can improve the ED regeneration. Its performance was then investigated and optimized by the Taguchi method, and the percentage contribution of each factor was evaluated by analysis of variance [479]. The optimal initial concentration was 27.5 wt% (without concentration difference, as expected). The applied current was the main parameter affecting the energy consumption, but had mild effects on the concentration increase. Instead, it was mostly affected by the concentration difference between the two solutions (accounting for ~78% of the effects) and, at a lesser extent, by the initial regenerate concentration. Compared to average values, optimal conditions led to an energy saving of 31.7% (*E_spec_* ≈ 25 kWh/m^3^) and to a concentrative effect increased by 9.4% (regenerate at 36.22% wt).

**Figure 25:**
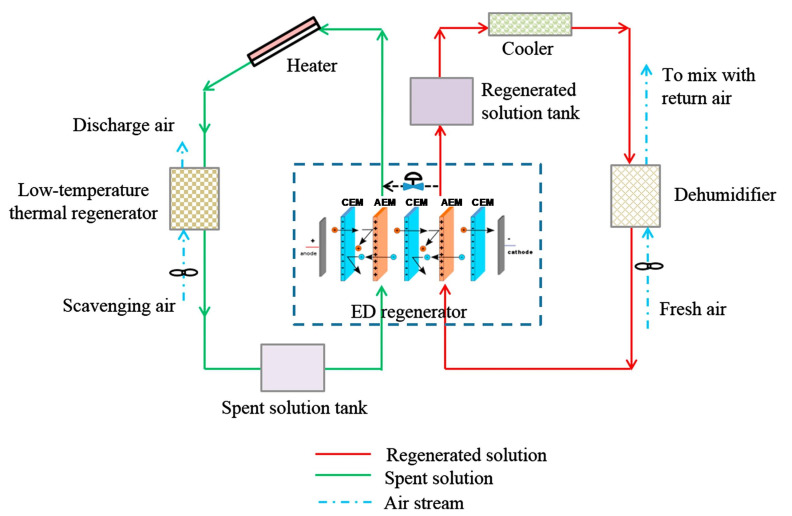
Scheme continuous ED regeneration integrated into a liquid desiccant dehumidification system. Reproduced (adapted) with permission from [480], published by Elsevier, 2019.

A hybrid method combining continuous ED and thermal regeneration of the spent solution by a low-temperature heat source was developed [480] (Figure 25). The technical feasibility of the system was assessed by modelling validated by ED experiments. Simulations of one week’s summertime weather in Darwin, Australia, showed that the two outlet concentrations from the ED regenerator were maintained at 29.93–30.17 wt% and 26.70–26.85 wt%, respectively (from inlet concentrations of 29.80–30.05 wt% and 26.80–26.95 wt%). The computed water removal in the low-temperature thermal regenerator and water absorption in the dehumidifier amounted at 128.6 kg and 126.6 kg, respectively. The largest part of energy for desiccant regeneration was consumed by ED (85%, corresponding to *E_spec_* of 22 kWh/m^3^), while the COP was on average equal to 0.5, thus suggesting that high-performance IEMs are crucial for the system efficiency. Experiments were designed by Taguchi’s method, and results were analysed by analysis of variance, showing that increasing inlet temperatures from 30 to 45 °C and from 20 to 30 °C for the spent and regenerate solutions, respectively, resulted in an enhancement of regenerate concentration increase of 19% and to a similar energy saving [481]. These tests confirmed that a higher initial concentration of the regenerate causes higher *E_spec_* and a lower concentration increase, which is considerably affected also by the initial concentration difference.

The ED regeneration of LiBr solutions showed lower values of *η* (9–31%) compared to LiCl solutions, because of the higher concentration (~45 wt%) [482]. As the operating conditions were let to vary, *η* and the COP changed significantly. However, a maximum COP of 4.26 was reached. A mathematical model simulated LiCl, LiBr and CaCl_2_ solutions regeneration, confirming that initial concentration and applied current are important factors in all cases [483]. Concentrations of 15, 25 and 15 wt% were suggested for LiCl, LiBr and CaCl_2_ solutions, respectively (*η* ≈ 70% and *E_spec_* ≈ 0.1 kWh/mol).

## 6. Waste Brine from Desalination or Ion Exchange

Desalination technologies provide an important contribution in response to water scarcity. The global desalination capacity reached ~100×10^6^ m^3^/day [484,485], by producing ~142 × 10^6^ m^3^/day waste brine [485]. Therefore, brine management is a critical issue facing the desalination industry. RO holds ~60–70% of the desalination market share [485,486] and has several applications. About 50% of RO plants produces potable water from brackish or sea water, ~40% provides ultrapure water to industries, and some installations reclaim polluted or waste effluents, or process food [487]. Brackish water RO (BWRO) has water recoveries of 85–90%, while seawater RO (SWRO) of 35–50%, limited by high osmotic pressure [487]. RO retentate is usually discharged or evaporated. Similarly, environmental impacts are related to the disposal of IXRs regeneration spent brines and ED desalination brines. However, novel brine management methods at low environmental impact are oriented by (near) ZLD strategies towards waste disposal minimisation and resources (water and others) recovery [488,489,490]. Membrane processes are under study for this aim [1,488,489,490]. In particular, ED techniques may recover water and salt (Section 6.1), acid and base (Section 6.2), or energy (Section 6.3).

### 6.1. Water and Salt Recovery

ED has been studied to recover water and salt (diluate and concentrate, respectively) from BWRO brine (Section 6.1.1), SWRO brine (Section 6.1.2) or wastewater RO (WWRO) brine (Section 6.1.3) in ZLD approaches. Moreover, ED has been proposed for the regeneration of spent NaCl brines from IX (Section 6.1.4).

#### 6.1.1. BWRO Brine

ED of BWRO brine can boost water recovery (diluate product) and facilitate the salts separation (concentrate product) in ZLD desalination.

**Figure 26:**
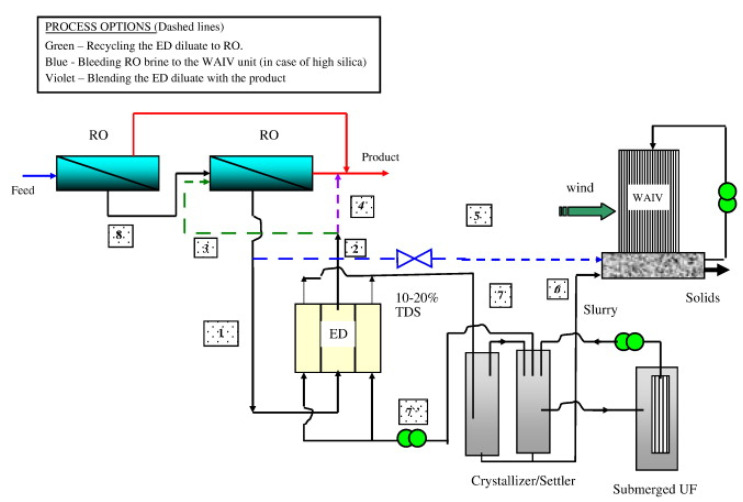
BWRO-ED desalination process. EDR treats the RO retentate (1). The EDR diluate product (2) can go through the second RO step (3) or, if it is sufficiently desalted, is conveyed with the permeate (4). The EDR concentrate (7) is sent to the seeded crystallizer/settler, and to the submerged UF module, thus the crystal-free concentrated stream (7′) recirculates to the EDR. The bleed stream (6) is evaporated by the WAIV unit. Reproduced with permission from [492], published by Elsevier, 2010.

A pilot EDR treated BWRO brine, and a gypsum precipitator for the ED concentrate protected the stack, since CaSO_4_ was near to saturation [491]. The diluate salt concentration decreased from ~340 mN to ~20 mN, allowing for a total water recovery of 97–98%. Other performances were: concentrate concentration increase to 10% (from 1.5%), *η* = 60–75%, *E_spec_* = 7–8 kWh/m^3^. Despite the slow transport, the attainment of silica saturation level limited the brine concentration. Concentration and water recovery were similar for the BWRO-ED system depicted in Figure 26 (feed concentration ~0.3%, retentate ~1%) [492]. Scaling was minimized by acidification, EDR operation, and alleviation of sparingly soluble salts super-saturation via crystallisation, settling and MF or UF. Wind-aided intensified evaporation increased finally the TDS concentration over 30%. *η* was of 81%, *E_spec_* was of 5–6 kWh/m^3^, and the estimated cost was 0.408 €/m^3^ for treating 100 m^3^/h feed with 98% water recovery, thus showing the process competitiveness.

Pilot tests were conducted with a commercial-size ED unit (two electrical stages and four hydraulic stages) fed by BWRO brine (~10 g/L TDS, water recovery of 82.5%) [493]. ED recovered 55% of its feed, raising the overall recovery to 92.1%. MVMs obtained by modification of commercial IEMs changed the product composition. Compared to original IEMs, the MVMs ED achieved the same conductivity reduction (up to 60% of 19.5 mS/cm), while requiring higher *E_spec_* (up to ~70% more, ~4 kWh/m^3^). Efficient concentrative ED operations are designed by multi-stage configurations within more complex schemes. For example, two- or three-stage batch ED with concentrate split concentrated a 3.5 wt% NaCl solution to 17.9 or 20.6 wt%, with average *η* = 82.9% or 84.8%, and *E_spec_* = 0.31 or 0.45 kWh/kg salt (18.83 and 27.06 kWh/m^3^), respectively [494].

#### 6.1.2. SWRO Brine

SWRO brine treatment via ED recovers water and/or a high-concentration NaCl stream usable to produce coarse salt (via evaporation-crystallisation) or for other purposes, e.g., as raw material for the chlor-alkali industry.

**Figure 27:**
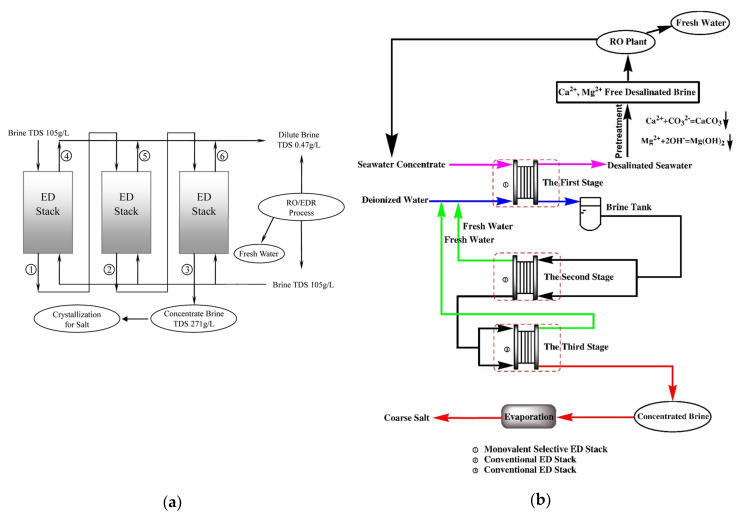
Flowcharts of three-stage ED systems for water reclamation and salt production from SWRO brine: (**a**) conventional ED; (**b**) ED with MVMs in the first stage, followed by conventional ED in the second and third stage. Panel (**a**) is reproduced (adapted) with permission from [495], published by Elsevier, 2014. Panel (**b**) is reproduced (adapted) with permission from [499], published by Elsevier, 2017.

ED stacks assembled with different IEMs were used with a simulated SWRO brine (10.5% TDS) [495]. A multi-stage operation (Figure 27a) produced two solutions with concentrations of up to 27.13% and 470 mg/L. The concentrated brine was suitable for producing edible salt, the diluate (water recovery of ~68%) was suitable more for industrial use than for drinking.

A real SWRO brine (70 g/L TDS) was concentrated through a pilot ED unit (single-pass diluate) equipped with MVMs, tested for 24 months to produce a feed for the chlor-alkali industry [496]. *η* ranged from 80% to 92% for Cl^−^. The concentrate stream was even depleted in most multivalent ions because of water transport. Instead, the Cu and Ni concentration increased due to the transported monovalent Cl-complexes. Better performances were observed at higher temperatures (27 °C), which led the salt concentration to a maximum of 245 g/L. *E_spec_* values comparable with the target of ED plants producing edible salt from seawater (0.12 kWh/kg salt, > 200 g/L) were obtained at lower concentrations (185 g/L). Instead, a process enhancement would be needed to meet the requirements of electrolysis for chlorine production (300 g/L, high purity) along with competitive costs. Experimental data from the same pilot plant validated a modelling tool [497]. Model predictions highlighted again that the RO-ED system was competitive in the edible salt market, but did not reach the targets for industrial use. Among different synthetized MVCs, the best permselectivities measured with a model SWRO brine were $P_{Na^{+}}^{Mg^{2+}}=0.09$ and $P_{Na^{+}}^{Ca^{2+}}=0.8$, which can provide concentrates at purity sufficient for the chlor-alkali process [498].

A three-stage ED was developed for recovering water and salt from SWRO brine (~45 g/L TDS, 60 mS/cm) [499] (Figure 27b). The first ED was performed with MVMs to retain divalent ions in the diluate feed and transfer NaCl to the concentrate (deionized water). The produced diluate (70% desalination) can return to RO after removal of divalent ions, while the concentrate went through two concentrative steps with conventional IEMs. The conductivities of the concentrate products were 42.4, 73.2 and 105 mS/cm, and coarse salt 85% pure in NaCl was produced by final brine evaporation. The water recoveries relative to the initial diluate of each stage were 90%, 86% and 82%, obtaining fresh water from the last two stages. The total *E_spec_* was of 2.3–2.4 kWh/kg NaCl.

Another strategy for enhancing the salt purity consists of using an NF pre-treatment, reducing the divalent ions concentration [500]. From an artificial SWRO brine (66.8 g/L TDS), the NF rejected Ca^2+^, Mg^2+^ and ${\mathrm{SO}}_{4}^{2-}$ by 40, 87 and 100%, respectively, recovering water by 54.3% as permeate (58.7 g/L TDS). The ED tests achieved a maximum concentration of 160 g/L NaCl with some impurities (5 g/L) at *E_spec_* of 1.4 kWh/kg NaCl. The final ED diluate (~95% of the NF permeate) had a minimum concentration of 25.25 g/L TDS, hence it could go to the RO. Similarly, the NF retentate could go to further recovery processes.

Theoretical approaches, including process simulators, techno-economic estimations and thermodynamic evaluations, can drive the design of optimized systems. Several hybrid schemes and multi-stage operations are viable, prospecting promising developments [501,502,503,504]. A techno-economic analysis found that the salt production costs of an SWRO-ED-crystallizer plant (RO feed at 50 m^3^/h) may be competitive (61–111 $/ton salt), i.e., lower by 19–55% than those of conventional standalone ED-crystallizer systems [505]. However, the process main aim (brine minimisation or salt production) and the site-specific features (water, salt and electricity prices) may require different strategies. For example, in another analysis, feeding the ED diluate with SWRO brine instead of seawater reduced the water production costs by 87% (i.e., from 27 to 3.5 $/m^3^), but increased the salt production costs by 26% (i.e., from 135 to 170 $/ton salt), by considering an SWRO plant capacity of 150,000 m^3^/day [506]. Optimal currents further reduced the water costs (3.0 $/m^3^), but increased *E_spec_* by 26% to 12.7 kWh/m^3^. The complete salt production scheme (SWRO-ED-crystallizer) was competitive with the SWRO desalination only in some Middle-East countries, where the salt price is higher than 104.5 $/ton.

**Figure 28:**
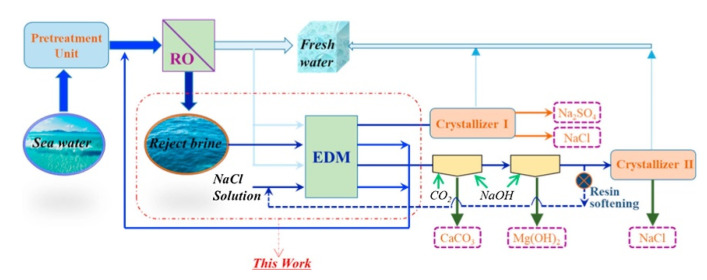
Conceptual scheme of SWRO-EDM for water and salts recovery. Reproduced with permission from [41], published by Elsevier, 2019.

EDM of BWRO [507] or SWRO [41] brine was proposed. The complete conceptual scheme of SWRO-EDM is depicted in Figure 28. EDM separates ions from SWRO brine (diluate 1) into two high-solubility salt solutions products (the initial feed is SWRO permeate), one with Na^+^ salts (concentrate 1) and another one with Cl^−^ salts (concentrate 2), following the metathesis MX + NaCl → NaX + MCl, where NaCl is supplied by the substitution solution (diluate 2). The diluates recirculation to RO increases water recovery, crystallizer I recovers Na_2_SO_4_ and NaCl from concentrate 1, crystallizer II recovers NaCl from concentrate 2 after Ca^2+^ and Mg^2+^ precipitation. In the EDM experiments, initial solutions were an SWRO retentate at 50 g/L TDS, an NaCl substitution solution with the same conductivity, and deionized water (concentrates). To find optimal operating conditions, preliminary experiments with partial concentration were performed, exhibiting *E_spec_* values lower than 1 kWh/kg salt. Then, a maximum concentrates concentration of ~200 g/L TDS was attained (while desalinating the diluate products at 5 mS/cm) with negligible co-ion leakages and scaling. However, 170 g/L was the concentration recommended to preserve the process efficiency. The feasibility of the overall process scheme should be assessed by further studies.

#### 6.1.3. WWRO Brine

ED was studied to recover further water from RO brines produced in industrial/municipal WWTPs (WWRO brine).

At an industrial WWTP of ~1900 L/min (heavy metal clarification, settling UF, RO), a six-stack EDR plant treated ~265 L/min WWRO retentate with TDS at 3000–5000 ppm [508]. The EDR was designed for *E_spec_* of 4 kWh/m^3^ and a cost of 0.33 $/m^3^. It reclaimed 85% of the WWRO reject brine as diluate (550 ppm), which recirculated to the treatment line, while the remaining 15% waste concentrate (45,000 ppm) went to evaporation ponds.

**Figure 29:**
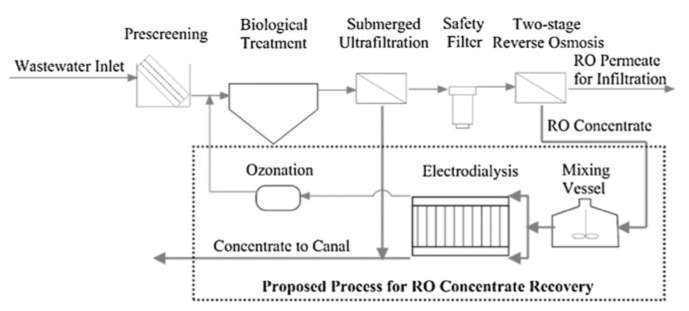
Scheme of the WWTP with RO-ED water recovery. Reproduced with permission from [509], published by Elsevier, 2011.

A WWTP receiving mainly a domestic effluent was endowed with UF and RO tertiary treatment to reuse water in groundwater recharge [509]. A pilot ED stack was tested to desalinate the WWRO retentate and recirculate it into the biological step (after ozonation preventing organics accumulation), while discharging the ED concentrate (Figure 29). The WWRO retentate had ~4.8 mS/cm conductivity, with high scaling potential (Ca^2+^, Mg^2+^ and carbonates), which was lowered by acidification-decarbonation with HCl. Batch or feed and bleed experiments provided a diluate with 75% desalination (average overall *η* ≈ 70–85%), while a long-run test (42 h) attained a 69% desalination, thus the effluent could be recirculated, though it needed a TOC reduction. The ED addition enhanced water recovery from 75% (standalone RO) to 95%. An ED operational cost of 0.19 €/m^3^ was estimated, 20% of which went from *E_spec_* of 0.9 kWh/m^3^ [510]. The capital cost was actually prohibitive (~15 €/m^3^) for a plant capacity of 300 L/h, but the significant abatement with a full-sized plant could make the system feasible. The membrane processes produced significant CO_2_ emissions, but if they were driven by renewable energy, the total emissions could be lower than those from conventional methods. The precipitation/crystallisation in a pellet reactor with fluidized bed showed that 80% of Ca^2+^ removal made the ED operation stable (*η* ≈ 70%) without any scaling [511]. The integration of a phytoremediation pre-treatment (willow field for nutrients and organics removal) with ED recycled effectively the treated WWRO concentrate in the WWTP [512]. However, again, an oxidation step after ED was needed.

A pilot plant integrating a membrane bioreactor with RO and EDR was developed for treating municipal landfill leachate (15 mS/cm, 2250 mg/L COD) [513]. The RO had a water recovery of 84% and a rejection > 95% for most components. The EDR produced a diluate product at 30–65 mS/cm and recovered 67% of water, leading to an overall water recovery over 93%. 

Recovery of nutrients and/or organics via ED from RO concentrate of food industry wastewater was assessed [514]. Experiments with model solutions (salts mixture, ~20 mM as total concentration) without or with organic compounds (120 g/L TOC) showed that monovalent ions and multivalent ions can be separated from each other under suitable operating conditions (low currents, for example), especially by using MVAs. However, separating nutrients (${\mathrm{NO}}_{3}^{-}$ and $H_{x}{\mathrm{PO}}_{4}^{y-}$) from other ions (Cl^−^ and ${\mathrm{SO}}_{4}^{2-}$) was not feasible. The organics fate was strongly affected by molecular weight and charge, as the transport was slower for larger molecules and even slower for zwitterions and uncharged compounds. With a real WWRO retentate (90 mM Cl^−^, 4.5 mM ${\mathrm{SO}}_{4}^{2-}$, 70 ppm TOC), ions were almost completely removed, while more than 85% of the organics was retained within the diluate.

When RO treats wastewater, organic micropollutants (e.g., pesticides) may interact with the IEMs functional groups and may by adsorbed by the IEMs, with important implications such as fouling/poisoning and release during cleaning [515].

Some ED applications were studied for desalinating RO brine from petrochemical industry wastewater treatment, showing interesting results despite the scaling and fouling potential. A pilot EDR exhibited removal efficiencies above 90% for Cl^−^ and alkalinity, and of 76% for TDS, from a WWRO brine with 1104 mg/L TDS, and this RO-EDR process enhanced significantly the water recovery (87.3%) [516] compared to the EDR-RO scheme [352] (41%, see Section 4.3.2), thus offering the chance of reuse (cooling towers). Another EDR study achieved TDS removal of 50% (from 8663 mg/L TDS in the feed) with 85% of water recovery that reduced the brine volume by ~6.5 times [517]. No organic fouling was observed; however, the stack resistance increased during operation due to scaling at the concentrate side. A comparison among ED, NF and IX assessed the separation of NaCl and natural organic matter (NOM) [518]. The WWRO retentate had 6.9 g/L TDS and 35 mg/L NOM. ED removed ~90% of NaCl and recovered ~97% of water, retaining more NOM (e.g., TOC by 65%) when using MVMs. All the tested technologies exhibited results opening new opportunities to recover solid NaCl from brines in ZLD perspective.

The response surface methodology modelled and optimized the ED for RO concentrate (1950 mS/cm) reclamation in coal-fired power plants, finding a reduction in conductivity of 75.3% with *E_spec_* of 0.11 kWh/m^3^ and ~50% of water recovery with optimized conditions [519].

**Figure 30:**
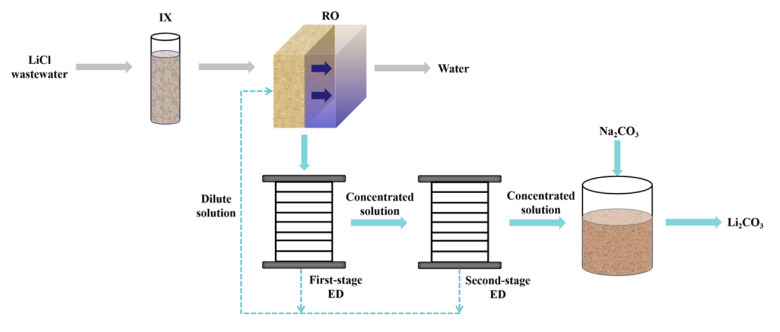
Scheme of the IX-RO-ED system for concentrating LiCl from industrial lithium-containing wastewater. Reproduced with permission from [520], published by American Chemical Society, 2019.

An IX-RO-ED treatment process for industrial Li-containing wastewater was developed [520] (Figure 30). The effluent (1268.9 mg/L Li^+^, 17.87 mS/cm) from a Li-ion batteries production plant was softened to prevent scaling. Then, it was concentrated by RO and a two-stage ED, thus obtaining fresh water (RO permeate) with increased recovery (ED diluate recycle) and a concentrate solution suitable for Li_2_CO_3_ precipitation by Na_2_CO_3_ addition. Under optimal conditions, the RO retentate (60 mS/cm) was split into diluate/concentrate ED feed solutions with 3:1 volume ratio, obtaining water recoveries of 67.51%, 78.73%, and 69.44% in the RO step, the first and the second ED, respectively (*E_spec_* in ED of ~30 and 50 kWh/m^3^). A final LiCl concentration of ~87 g/L was reached with average *η* of 67.52%, total *E_spec_* of 0.772 kWh/kg and a total cost of 0.47 $/kg (process capacity of 282 kg/year).

#### 6.1.4. IX Spent Brine

**Figure 31:**
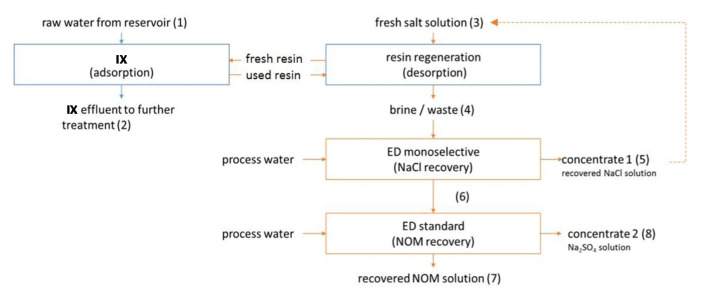
Process scheme of IX water production and two-stage ED treatment of brine. (1) Surface water from IJssel lake (the Netherlands), (2) ceramic MF and advanced oxidation, (3) NaCl regenerating solution, (4) spent regeneration brine, (5) concentrate from monovalent selective ED, (6) diluate from monovalent selective ED, (7) NOM-containing diluate from conventional ED, (8) multivalent salts-containing concentrate from conventional ED. Reproduced (adapted) from [521], published by Elsevier, 2019.

Studies on ED regeneration of spent brines from IX treatment of surface water have exhibited promising results. A two-stage pilot ED treated an NOM-containing spent brine from anion exchange resin regeneration [521] (Figure 31). Electrostatic interactions and formation of metal-organic complexes caused the NOM removal by IX and the transfer of these compounds to the regeneration brine. The spent NaCl brine (8530 mg/L Na^+^, 9050 mg/L Cl^−^) contained NOM at 700 mg/L as dissolved organic carbon, but also ${\mathrm{NO}}_{3}^{-}$ (113 mg/L), ${\mathrm{SO}}_{4}^{2-}$ (3350 mg/L) and ${\mathrm{HCO}}_{3}^{-}$ (2660 mg/L). The first ED step was conducted with MVAs, while the second one was with standard membranes. Both the ED steps used fresh water (RO permeate) in the concentrate, while the spent brine flowed through the diluate of both stages. As a result, with a removal of 85% for Cl^−^ and 65% for Na^+^, the ED stage 1 produced a monovalent salt solution (concentrate) with sufficient quality to be reused for IX regeneration, while the ED stage 2 produced a multivalent salts solution (concentrate, with predominance of Na_2_SO_4_) and a 470 mg/L NOM solution (diluate). The potential reuse of the NOM solution should be further studied, since its quality was impaired by heavy metals.

A simpler process was adopted for an IX spent brine with NaCl, sulphate and NOM concentrations of 90, 1, and 1 g/L (as dissolved organic carbon), respectively [522]. A single ED step (spent brine as diluate, 2 g/L NaCl solution as concentrate) with MVMs was effective, yielding a pure NaCl solution with 88.8% removal, negligible membrane fouling, and *E_spec_* of 2 kWh/kg salt.

### 6.2. Salt Conversion into Acid and Base

BMED can valorise brines by salt transformation into acid and base. Moreover, the desalted feed may improve water recovery (ZLD approach). Studies on this application are presented for BWRO brine and SWRO brine in Section 6.2.1, and for WWRO brine and IX spent brine in Section 6.2.2.

#### 6.2.1. BWRO Brine and SWRO Brine

The cost-effectiveness of acid/base production was demonstrated with artificial solutions (up to 390 mM NaCl) representing also BWRO concentrates [269] (see Section 4.2.2).

Several studies have been devoted to concentrated solutions from seawater desalination. A model SWRO brine was prepared with a salts mixture (~61 g/L) without Ca^2+^ and Mg^2+^, and BMED produced mixed acids and bases up to 1 M [523]. The feed was desalinated up to 80% (from 70 to 12 mS/cm), but its pH decreased to ~2 due to proton leakage. *η* values between 50% and 80% indicated co-ion leakages as well, and impurities (e.g., ${\mathrm{SO}}_{4}^{2-}$ in the acid) made the products unsuitable to reach commercial chemicals quality standards. However, they could be intended for uses not requiring high purity. The employment of nanocomposite MVAs synthetized by commercial AEMs coating, led only to a 10% reduction of ${\mathrm{SO}}_{4}^{2-}$ in the acid compared to the original AEMs (~6 mM) [524]. However, the MVA was stable over more than 90 h operation ($P_{Cl^{-}}^{SO_{4}^{2-}}$ ≈ 0.8 = constant, against ~1.08 for the AEM). By using a 1 M NaCl feed and a photovoltaic solar array simulator, a BMED process powered by photovoltaic energy was characterized [525]. In overflow configuration, the manipulation of flow rate for pH control resulted in a drop in *E_spec_* from 7.3 kWh/kg acid (reference case at constant current) to 4.4 kWh/kg at variable current. An acid stream at a constant concentration of ~1 M HCl was produced for 30 h. Other BMED experiments produced up to 3.31 M HCl and 3.65 NaOH (from 1 M NaCl feed) [526]. *E_spec_* was in the range 21.8–41.0 kWh/kg HCl at constant currents, and 26.7–43.5 kWh/kg HCl at variable currents. Azeotropic distillation was simulated as post-concentration step, producing 11.4 M HCl (35 wt%, commercial level) with overall *E_spec_* of ~40–60 kWh/kg HCl. A life cycle assessment included environmental burdens associated with brine disposal and carbon footprint [527]. It was shown that renewable energies can be crucial for an overall process sustainability. However, though photovoltaic energy reduced the carbon footprint of the BMED-distillation process, it was still higher than that of industrial production from H_2_-Cl_2_ reaction, excluding the contribution of the transportation [526].

A real brine from seawater desalination (~42 g/L) pre-treated for Ca^2+^ and Mg^2+^ removal (precipitation by NaOH and CO_2_) was used [528]. Preliminary tests were conducted with model solutions for optimisation purposes, finding *η* = 50–74% and *E_spec_* ≈ 7.5 kWh/kg. With the real effluent, the BMED produced continuously ~1 M acid and base without visible fouling. These products were usable at the desalination plant. For other industrial uses necessitating high quality standards, they would require purification. The low-salinity diluate product enhanced water recovery (direct reuse or recirculation to RO).

An SWRO brine (~60 g/L) was cleaned from Ca^2+^ and Mg^2+^ by NF and chemical precipitation, then the effluent (52 g/L) went to BMED [529]. The feed was almost fully desalted, and the NaCl conversion into HCl and NaOH was over 70%, obtaining ~1 M products at *η* of ~77% and *E_spec_* of ~2.6 kWh/kg NaOH. Improvements were achieved with a combined process where an SWRO brine (~70 g/L) was purified and concentrated (~100 or ~200 g/L) by ED with MVMs, and then was converted (1.6 or 2 M acid, 1.2 or 2 M base) by BMED [530]. The diluate produced by BMED was at ~20 mg/L, the conversion was between 46% and 84%. The ED *E_spec_* was 0.055–0.217 kWh/kg NaCl, the BMED one was 1.82–3.62 kWh/kg NaOH (*η* = 55–88%). The associated operating cost exceeded the market prices of the products. Therefore, in situ uses and/or stringent brine management regulations could justify the process.

**Figure 32:**
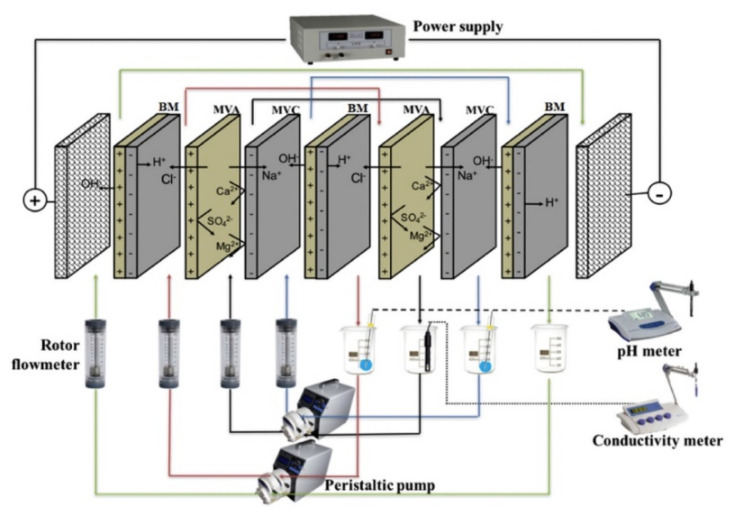
Scheme and experimental set-up of BMSED for acid/base recovery from SWRO brine. Reproduced with permission from [531], published by Elsevier, 2018.

BMED equipped with MVMs (bipolar membrane selectrodialysis, BMSED) was used with SWRO model brines at 70 or 105 g/L [531] (Figure 32). The process, which used commercial membranes, was highly selective ($P_{Ca^{2+}}^{Na^{+}}$ = 3.6–10.6, $P_{SO_{4}^{2-}}^{Cl^{-}}$ = 31.0–67.5) and thus produced high purity (approaching 99.99%) HCl and NaOH solutions at concentration up to 1.9 and 2.2 M, respectively. The final salt water was still at high concentration (~50 g/L) and could return to RO.

#### 6.2.2. WWRO Brine and IX Spent Brine

BMED for RO concentrates was actually first proposed for a WWTP effluent (2590 mg/L TDS, 9 mS/cm) softened by IX [532]. Mixed acids and mixed bases at ~0.2 N concentration were produced, along with a diluate at conductivity below 2 mS/cm. For an RO capacity of 37,850 m^3^/day, the estimated process cost of ~0.7 $/m^3^ was lower than those of conventional disposal (e.g., evaporation pond) or thermal ZLD processes.

BMED was proposed for waste neutralisation brine (20.4 mS/cm) from acid and base effluents regenerating IXRs used for surface water desalination [533]. The process concept included an ED step with MVCs followed by IX to concentrate and soften the brine and increase water recovery (ED diluate recycle) before BMED. Acid and base products from BMED were reusable for IXRs regeneration, and the produced diluate could recirculate to the ED concentrate. The saline water conductivity increased to 40 mS/cm by ED. BMED desalinated this solution up to ~5–10 mS/cm, and produced 0.9 M acid and base at *η* of 47% and *E_spec_* of 6.25 kWh/kg HCl. Higher concentrations were reached with worse performance, due to co-ion leakage and current leakage (shunt currents).

### 6.3. Energy Recovery

**Figure 33:**
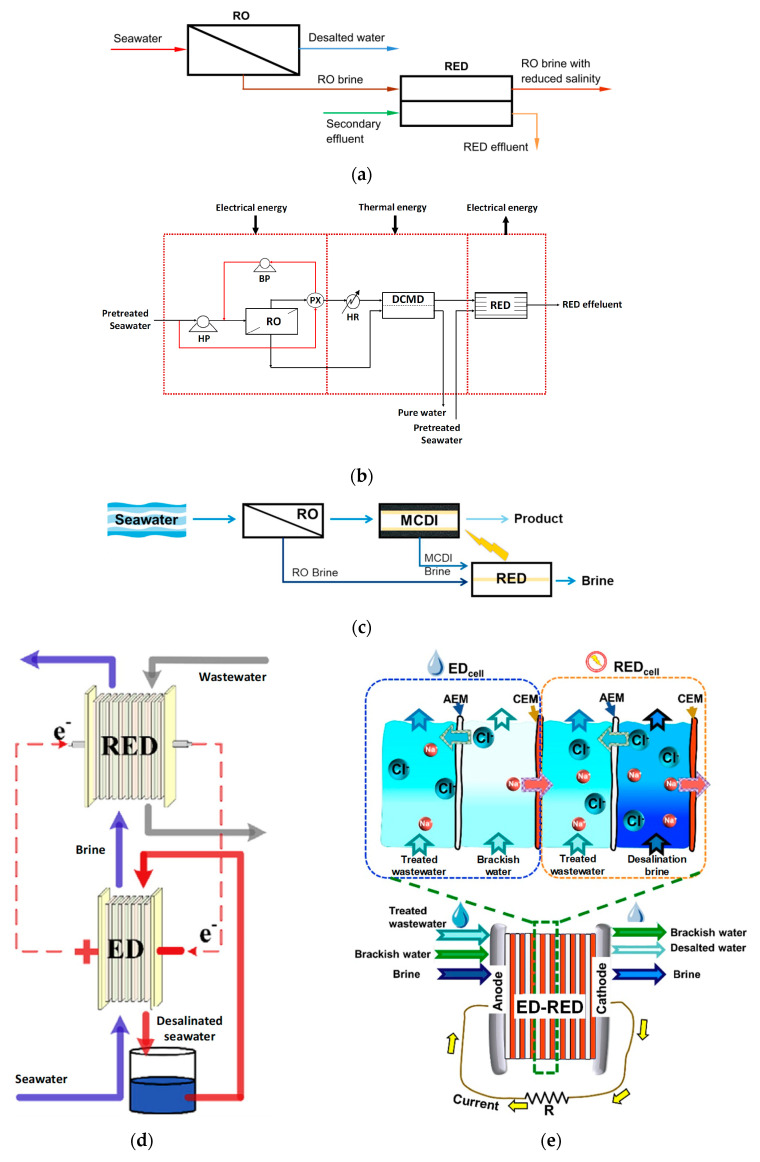
Desalination systems with RED using desalination brine for recovering energy: (**a**) RO-RED scheme in which RED receives the SWRO brine and a secondary effluent; (**b**) RO-MD-RED scheme; (**c**) RO-MCDI-RED scheme; (**d**) ED-RED scheme; (**e**) ED-RED process integrated in single stack (four-channel repetitive unit, coupling an ED cell with an RED cell). Panels (**a**–**e**) are reproduced with permission from [424,536,537,538,539] (adapted), respectively, all published by Elsevier, 2013, 2019, 2019, 2017 and 2020, respectively.

The valorisation of desalination waste brines can be done in the form of energy recovery through salinity gradient power technologies [534,535]. Brines from RO desalination (of seawater, typically) can be employed as high-salinity feed (concentrate) for RED to produce electrical energy, reducing the overall energy consumption of desalination. Moreover, the RO brine is partially desalted prior to discharge. RED units require also a low-salinity feed; thus, using reclaimed wastewater as RED diluate can enhance the energy recovery compared to that achievable when using the desalination plant influent, e.g., seawater. Figure 33a shows the RO-RED scheme in which seawater is desalinated by RO, and RED receives the SWRO brine and a secondary effluent [424]. This scheme avoids issues of seawater contamination by organic micropollutants that, instead, may occur in the opposite scheme, i.e., RED-RO (Section 5.2). However, the overall energy balance may be less favourable in the RO-RED configuration, despite the higher power produced by RED due to the higher-concentration concentrate. For example, model predictions of the energy consumption are ~1 kWh/m^3^ for RO-RED and ~0.5 kWh/m^3^ for RED-RO [424]. More complex schemes were assessed, i.e., with RED pre- and post-treatment or with brine recirculation, but similar energy performances were predicted. In all cases, the costs should be evaluated.

Using desalination brines as concentrate, energy recovery by RED has been evaluated with different feed solutions and within different desalination schemes. Figure 33 depicts some of them.

Some integrated schemes involve a third process, e.g., membrane distillation (MD, Figure 33b) or membrane capacitive deionisation (MCDI, Figure 33c), between RO and RED, enhancing water recovery and/or energy saving. In the ED-RED coupling shown in Figure 33d, RED uses the ED brine (seawater desalination) and treated wastewater. ED and RED offer the possibility of internal integration in one stack with four-compartment repeating units (Figure 33e) and could by coupled with other desalination processes (e.g., RO) by using their brines as high-concentration streams boosting the energy recovery.

Table 4 reports several studies on energy recovery via RED using desalination brines, including those illustrated in Figure 33. As shown in Table 4, *P_d,max_* spans in a wide range in the order of ~1 W/m^2^. It was shown that the addition of RED can reduce the energy consumption of desalination. Promising results were exhibited by ED-RED couplings, thus posing the bases for the development of self-sufficient or low-energy consuming systems. In all cases, however, a critical issue is represented by the capital costs.

## 7. Discussion, Conclusions and Outlook

ED and unconventional configurations of ED, i.e., BMED, SED, EDM and EDI, have great potential in desalination and valorisation strategies of wastewater for a broad range of applications to recover water and/or other valuable components. The main ones are metals, salts, acids and bases, nutrients, and organics. Energy recovery via RED is another possibility.

ED methods can be applied for effluents originating from various industrial processes (Section 4). In the separation of heavy metal ions (Section 4.1), such as Ni, Cu, Zn, Cr, Cd, and Pb, ED can provide solutions suitable for reuse, e.g., plating baths and rinse waters, including solutions with complexing agents (cyanide or organic acids), and tanning solutions. Two-stage operations (either both or one with MVMs) can improve purity, while EDI can reduce the energy consumption when treating diluted solutions (including low radioactive effluents). ED techniques (including complexation-enhanced ED) can also produce reusable solutions from heavy metal ions mixtures. BMED and SED have been poorly studied so far, but have exhibited promising results. Future research should be focused on experiments with real effluents, by assessing long-term operations, and should aim at scaling up the systems. Cost analyses are needed to assess the techno-economic feasibility.

For the regeneration of acid/base and salt conversion (Section 4.2), ED methods have been studied with a variety of industrial wastewaters. In the presence of heavy metal ions (waste solutions from pickling and other metallurgical processes), the use of proton-blocking AEMs and proton-selective CEMs is crucial for recovering acids by ED concentration. Interestingly, NF membranes instead of CEMs can increase significantly the proton/metal permselectivity. Further studies on the development and testing of special membranes would be beneficial. BMED is a valid alternative and can also convert salts into acids and bases, either with or without previous precipitation of heavy metals, if present. The regeneration of spent solutions from chemical absorption of flue gases (SO_2_, H_2_S, CO_2_) is an interesting application of ED techniques, which may offer economic advantages. Various effluents containing organic matter were treated as well, finding only minor fouling issues in many cases. There are some commercialized systems and pilot plants. The scaling up should be extended to a wider range of applications.

Desalination (Section 4.3) via ED enables water reuse by treating salty wastewater from different industrial sources. Produced water of oil and gas extraction poses challenges related to high energy consumptions in the case of high-salinity feeds. Preliminary studies show that optimized systems can be competitive even for these solutions, but significant efforts are still needed in this direction. Dealing with fouling, cleaning procedures and EDR operation may have a partial effectiveness, as also shown by pilot plants. However, pulsed electric fields can minimize fouling phenomena. Therefore, this technique deserves further studies. Pilot installations have reclaimed wastewater of refineries and petrochemical industries, drainage water of coal mines, and wastewater of power plants. The treatment costs were shown to be attractive in some cases, but further economic analyses are needed. Energy recovery via RED is an interesting option, thus it should be explored more deeply in the future, by paying attention to the investment costs. 

ED methods may be effective for various other industrial wastewaters (Section 4.4). Single salts or mixtures, waste effluents from pulp and paper manufacturing, textile processing, and bio-refining are just some examples. However, only a small number of studies have been conducted so far for each application. Therefore, further research is required to improve the performance (e.g., in terms of selectivity and energy consumption/recovery) and to develop techno-economically competitive systems for the various types of industrial wastewater.

There are several possible applications for municipal wastewater and other effluents (Section 5). In the desalination of municipal WWTP effluents (Section 5.1), ED can be a cost-effective treatment enabling water reuse (e.g., irrigation), as shown by several field plant applications. Interesting results were shown by some studies on ED coupled with FO for recovering high-quality water or for controlling salinity build-up, thus this coupling is deserving of more attention in the future.

Treated wastewater may be used as a low-salinity stream coupled with seawater or other waste effluents as high-salinity stream in order to recover energy via RED (Section 5.2). The presence of organics requires the development of cost-effective pre-treatments and cleaning methods against fouling and clogging, enabling long-term operations with stable net power. Some pilot installations have been tested, but techno-economic analyses are needed. The integration of membrane distillation with RED has been proposed to recover water and energy from urine, but further studies are needed to assess the process feasibility.

The recovery of nutrients (Section 5.3) via SED or ED is another option, but energy consumptions and costs still need to be evaluated. Fertilizers can be produced also by ED treatment of excess sludge sidestreams, human urine and animal farming effluents. Pilot installations were operated, and the processes may be competitive. Pre-treatments (in the case of human urine) or cleaning and polarity reversal (in the case of digested swine manure) minimized fouling phenomena, and thus long-term operations may be feasible. In a couple of studies, VFAs were recovered along with nutrients by ED (from excess sludge) or BMED (from pig manure). Promising results were obtained in a study on SED for the simultaneous fractionation of cations and anions into several streams from digested swine manure. These emerging applications deserve further studies.

A possible application of ED in the buildings sector is the regeneration of liquid desiccant solutions for air conditioning (Section 5.4). The high-concentration solutions (for example ~30 wt% LiCl) used for this process require the development of high-performance IEMs and a delicate process optimisation in order to limit detrimental effects due to undesired transport phenomena.

Resources can also be recuperated by treating waste brine from desalination or ion exchange (Section 6). Water and salt recovery (Section 6.1) has been demonstrated at pilot scale in various zero brine discharge systems. Scaling and/or fouling were controlled in all cases, without particular problems. ED exhibited competitive costs in recovering water from BWRO brine. The same applies for ED with MVMs recovering concentrated brines for edible salt production (evaporation–crystallisation) and diluted solutions (fresh or brackish water) from SWRO retentate, as shown also by long-term testing. However, the competitiveness with respect to a standalone SWRO depends on the local water, salt and electricity prices. Instead, NaCl recovery for industrial use (e.g., in the chlor-alkali industry) is not yet feasible. EDM has been proposed to separate salts, and it was found to be efficient. However, the overall process should be assessed. Some studies showed that water recovery by ED may be feasible also from WWRO brine (either municipal effluents or industrial effluents, e.g., from petrochemical sites). In the presence of organics (e.g., food industry wastewater), attention should be paid to their molecular weight and charge, which affect their transport. Water and salt (LiCl concentrated solution) were recovered from the effluent of a Li-ion batteries production plant, thus deserving further research to promote the industrial application. A couple of studies have investigated the treatment of surface water IX spent regeneration brines containing NOM. NaCl solutions that were reusable for IX regeneration were obtained via ED with MVMs, thus minimizing waste brine disposal. Further studies should be focused on the potential reuse of the NOM solution, the process sustainability and the long-term operation.

Salt conversion into acid and base from waste brines (Section 6.2) is a recovery method that can be performed by BMED. Moreover, the desalted solution may improve water recovery. In most cases, the treatment was tested with SWRO brine. Acid and base recovered have not reached the quality standards of commercial chemicals. This, in conjunction with the high energy consumptions, does not yet allow market entry. However, in situ use at the desalination plant is possible. The use of MVMs significantly enhanced the purity of the products. Future research should be intensified in this direction, as well as in the development of highly selective membranes, in the process optimisation, in the evaluation of post-concentration systems and in the scaling up. 

Waste brines from desalination plants are suitable as concentrated solutions for energy recovery via RED (Section 6.3). Low-energy consuming systems were demonstrated in a few recent studies. However, capital costs may increase significantly, and thus economic analyses should reveal the actual feasibility. Other critical points for further research are the development of scaled-up systems, the testing with real solutions, and the evaluation of the net power density. Addressing all these aspects is necessary in order to attempt the implementation of integrated methodologies in real systems.

The application of ED techniques in wastewater treatment offers new opportunities for environmental protection and recovery of resources. Techno-economic challenges are still present, but great efforts have been made, mainly in the last 20 years, opening promising perspectives within efficient ZLD systems. Some commercial applications and several pilot installations are accompanied by hundreds of studies with laboratory tests.

Some process limitations can be alleviated or even remediated. EDR operation, pulsed electric fields, pre-treatment and cleaning procedures against fouling can maintain or restore, at least partially, IEM properties. However, permanent fouling and poisoning of membranes may occur. Special membranes, e.g., proton-blocking AEMs, proton-selective CEMs, monovalent selective membranes, and even UF or NF membranes, can improve process selectivity and products purity. Nevertheless, energy consumptions may be high. Therefore, in addition to developing high-performing membranes (low resistance, high selectivity, low osmotic transport), optimizing system design and operation is essential to implement competitive processes. In this regard, novel concepts based on multi-stage ED configurations or integrated (electro-)membrane processes provide interesting technological solutions. Performance with real effluents, scaling up, long-term operation, overall sustainability, and techno-economic analysis have still to be assessed for several applications. An abatement in the membrane cost will be important to improving the process economics.

Please note that studies on similar or hybrid processes have been growing, i.e., EDI with configurations deviating from conventional ED stacks (important role of electrode chambers) [42,45,46,47,183], RED and fuel cell (Fenton)-RED with wastewater treatment at the electrode compartments [53,57,544,545,546,547], concentration gradient or pH gradient flow batteries [548,549,550], membrane electrolysis and electro-electrodialysis [551,552,553,554,555,556,557,558,559,560], hybrid liquid membrane-ED [561,562,563], decoupled ED [564], shock ED [565], (membrane) capacitive deionisation [566,567,568,569,570,571,572,573,574], membrane electrode redox transistor ED [575], bio-electrochemical systems [576,577,578,579] including microbial desalination cell [580,581,582,583], microbial desalination and chemical-production cell [584], and (Fenton) microbial RED [585,586,587]. Hence, knowledge acquired on common aspects will likely promote substantial advances in ED.

In light of all of the above considerations, a realistic scenario where ED techniques will conquer a wider market share for real applications can be prospected for a not far future.

## Figures and Tables

**Table 1:** Search words used in the literature exploration on Scopus.

Search Word		Search Word
Electrodialysis	AND	Wastewater
Bipolar membrane electrodialysis		Effluent
Selective electrodialysis		Spent solution
Selectrodialysis		Recovery
Electrodialysis metathesis		Reclamation
Electrodeionisation		Reuse
Continuous electrodeionisation		Valorisation
Reverse electrodialysis		Regeneration
		Zero liquid discharge

**Table 2:** Other studies on industrial wastewaters without organic matter (adapted from [23]).

Wastewater	Treatment Process	Main Remarks	Ref.
NaCl + Na_2_SO_4_ solution, 0.01 M each	ED with MVAs	Layer-by-layer composite AEM, $P_{SO_{4}^{2-}}^{Cl^{-}}$= 11.5	[312]
NaCl + Na_2_SO_4_ solution, 0.05 M each	ED with MVAs	MVAs with hydrophobic alkyl side chain, max $P_{SO_{4}^{2-}}^{Cl^{-}}$= 13.1, long-term stability	[362]
NaCl + MgCl_2_ or LiCl + MgCl_2_ solutions, 0.1 M each	ED with MVCs	Zwitterion structure MVCs, $P_{Mg^{2+}}^{Na^{+}}$$=58.4\;\mathrm{and}\;P_{Mg^{2+}}^{Li^{+}}=6.5$	[363]
NaCl+MgCl_2_ solution, 0.1 M each	ED with MVCs	Zwitterion structure MVCs with hydrophobic alkyl side chain, max $P_{Mg^{2+}}^{Na^{+}}=25.3$	[364]
Model solutions with two salts with the same counter-ion among NaCl, Na_2_SO_4_, MgCl_2_, MgSO_4_ and NaNO_3_, 0.01 M each	ED or ED with MVMs	Max separation efficiency ~68% for cations by MVMs (comparable to NF), but lower for anions	[365]
NaCl + Na_2_SO_4_ solution, 8 mM each	SED	${\mathrm{SO}}_{4}^{2-}$ purity > 85%, *η* ≈ 50%	[39]
MgCI_2_ + Na_2_SO_4_ solutions, 0.3–0.5 M each	EDM	*η* > 100%, *E_spec_* ≈ 0.9–1.6 kWh/kg, MgSO_4_ purity ~98%	[40]
Na_2_SO_4_ solutions, 0.01 M/0.3 M	RED-alkaline polymer electrolyte water electrolysis	*V_OC_* ≈ 12 V (200 cell pairs), *P_d,max_* = 0.04–0.11 W/m^2^ by changing solutions velocity and temperature, H_2_ production 50 cm^3^/(h·cm^2^)	[366]
$\mathrm{Catalyst}\;\mathrm{plant}\;\mathrm{model}\;\mathrm{wastewater}\;({\mathrm{Na}}^{+},\;{\mathrm{Cl}}^{-},\;{\mathrm{Mg}}^{2+},\;{\mathrm{Ca}}^{2+},\;{\mathrm{SO}}_{4}^{2-}),\;25.8\;g/L\;\mathrm{TDS}$	Two-stage ED with MVCs	On-line membrane modification, $P_{Na^{+}}^{Ca^{2+}}$ reduced from 0.36 to 0.11, $P_{Na^{+}}^{Mg^{2+}}$ from 0.81 to 0.12, *η* = 75–92%, 1 g/L diluate, stable long-run, but larger water transport, membrane resistance, and *E_spec_* (up to ~35% more)	[367]
Photovoltaic industry simulated wastewater, 120–180 mg/L NaF and/or 750–2000 mg/L NaNO_3_	ED	With single salt, max removal efficiency ~60% and 75% for $F^{-}\;\mathrm{and}{\;\mathrm{NO}}_{3}^{-}$ in 6 min, under optimal conditions *E_spec_* = 0.25–0.36 kWh/m^3^; with mixture, ion competition affected only F^−^ removal	[368]
F^−^ solutions: single salt at 25–200 mg/L, binary and ternary mixtures with 100 mg/L $F^{-}+{\mathrm{Cl}}^{-}\;\mathrm{and}/\mathrm{or}\;{\mathrm{SO}}_{4}^{2-}$ at same equivalent concentration	ED	High removal efficiencies, *E_spec_* = 0.02–0.49 kWh/m^3^, Cl^−^ affected F^−^ separation, ${\mathrm{SO}}_{4}^{2-}$ did not	[369]
Synthetic secondary effluent of graphite industry, 10–30 mg/L NaF, 6 g/L NaCl	ED	Response surface methodology, F^−^ removal 99.69% with *E_spec_* = 0.76 kWh/m^3^ under optimal conditions	[370]
B artificial wastewater, 25–100 mg/L; binary or ternary mixtures with 100 mg/L B + ${\mathrm{Cl}}^{-}\;\mathrm{and}/\mathrm{or}{\;\mathrm{SO}}_{4}^{2-}$ at same or doubled equivalent concentration	ED	Max removal of B ~80%, enhanced at high pH (10.5) due to a predominance of $B{\left(\mathrm{OH}\right)}_{4}^{-}$, hindered by Cl^−^ and not by ${\mathrm{SO}}_{4}^{2-}$, *E_spec_* = 0.02–1.24 kWh/m^3^	[371]
Acidic model solution from B-selective sorbents regeneration, 0.2 M HCl or 0.1 M H_2_SO_4_ + 1.0 or 5.2 g/L H_3_BO_3_	Two-stage ED with pH increase	Regenerating acid (HCl or H_2_SO_4_) recovered in the concentrate ~90%, ~93% of H_3_BO_3_ (non-ionic) retained within the diluate and concentrated in the 2nd stage after alkalinisation, reusable solutions	[372]
B-containing industrial landfill leachate, 62.8–76.5 mg/L B (+ ${\mathrm{SO}}_{4}^{2-}$, Cl^−^, Ca^2+^ and Mg^2+^)	Two-stage ED with pH increase	Desalination in the 1st stage 80%, $B{\left(\mathrm{OH}\right)}_{4}^{-}$ removal in the 2nd stage 97% under alkaline conditions, max *η =* 25–28%, estimated cost 1.27 $/m^3^	[373,374]
Model nuclear power plant effluent, 60–400 mg/L H_3_BO_3_	Three-compartment EDI	Max removal ~45%, optimal pH = 10	[375]
NH_4_NO_3_ model wastewater from fertilizer production, 0.012 M	ED	Thin heterogeneous IEMs vs. commercial ones: higher limiting current density due to larger back-diffusion and electroconvection; lower alkalisation due to lower water dissociation	[376]
Synthetic solutions with single acid or salt: H_2_SO_4_, HNO_3_, NH_4_NO_3_, NaCl, LiCl, Na_2_SO_4_, 0.06–0.3 M	ED or BMED-ED	ED concentrator without flow through concentrate chambers, acid concentration 1.16 M at *η* = 89% for BMED and 26% for ED, *E_spec_* = 0.83 kWh/mol ${\mathrm{SO}}_{4}^{2-}$	[377]
Alkaline liquid from bauxite solid residue (Bayer process) washing (2.4 g/L ${\mathrm{Al}}^{3+},+K^{+},\;{\mathrm{Na}}^{+},\;F^{-},\;{\mathrm{SO}}_{4}^{2-}$…)	ED with aeration	NaOH recovery, NaAl(OH)_4_ separation, TDS and OH^−^ removal 61.3% and 76.6%, *η* = 60%, *E_spec_* = 11.15 kWh/kg	[378]
Synthetic or real wastewater from mineral carbonation for CO_2_ sequestration, 0.05–1.0 M (NH_4_)_2_SO_4_, 0.05–0.54 M (NH_4_)HSO_4_, (+MgSO_4_, NH_3_, Fe(II), Fe(III)…)	BMED	Different setups for regenerating rock-derived solutions after leaching or after carbonation, $E_{\mathrm{spec}}=1.7–350\;\mathrm{MJ}/\mathrm{kg}\;{\mathrm{NH}}_{4}^{+}$	[379]
Model solution from Li-ion waste batteries, Li^+^ and Co^2+^ 0.02 M each	BMED with complexation	Co-EDTA chelated anions and Li^+^ separated in the acid and base compartments, respectively, removals 99%, but Co absorption in AEM; metal recovery enhanced in semi-batch operation for the feed	[380]

**Table 3:** Other studies on industrial wastewaters with organic matter (adapted from [23]).

Wastewater	Treatment Process/Exp. Device	Main Remarks	Ref.
Solutions with octanoic acid or anionic surfactants; alkaline bleach plant filtrate from sulphate pulp mill, 1370 mg/L COD	IEM resistance measurement cell	Slight inorganic fouling on CEM by bleach plant filtrate, significant organic fouling on AEM by all solutes	[29]
Solutions with carboxylic acids (propanoic, octanoic and decanoic acid); alkaline bleach plant filtrate from sulphate pulp mill, 1850 mg/L COD	IEM resistance measurement cell, ED	No CEM fouling, AEM fouling due to organic anions, especially compounds with longer chain, and at higher currents	[381]
Solutions with 16 charged or neutral trace organic contaminants, 0.1 mg/L with 100 g/L NaCl	ED	Adsorption governed by electrostatic interactions, transport mostly diffusion driven, migration of charged components only at very low NaCl concentration	[382]
Solutions with NaCl, Na_2_SO_4_ or MgCl_2_ and acetic acid, phenol or glucose, 0.8 eq/L salts and 0.1 M organics	ED	Phenomenological model: convection-diffusion of neutral organics affected by steric effects and ion hydration	[383]
Solutions with NaCl, Na_2_SO_4_ or MgCl_2_ and acetic acid, phenol, glucose or acetate	ED	Phenomenological model: transport of several organics larger with ${\mathrm{SO}}_{4}^{2-}$ than with Cl^−^, opposite trend for phenol	[384]
Wastewater from bisphenol A diphenyl phosphate production, 4.5–4.8% total salt (NaCl and sodium phenolate), pH = 13.2–13.5, diluted with pure water	RED-ED	Ultrapure water fed into the RED diluate, *V_OC_* up to 1.65 V (10 cell pairs) and *P_d,max,net_* up to 1.12 W/m^2^ in RED at dilution ratio 1.0:0.5, *E_spec_* lower than that of standalone ED (17.65 vs. 25.32 kWh/m^3^) with 27.4% pre-desalination in RED	[385]
NaCl-glycerol solution, 1.11–1.67 M NaCl and 0.06–0.6 M glycerol	ED	7 membrane pairs tested, phenomenological model: C_3_H_8_O_3_ electro-osmotic co-transport 38–64%, osmotic co-transport 16–41%, diffusion 9–28%, low glycerol/NaCl flux at low glycerol/NaCl and NaCl concentrations	[386]
Simulated dairy wastewater, 10 mM citrate, 1 mM lactate, 30 mM NaCl…	ED	Guanidinium groups in AEM as functional moiety binding oxyanions, enhanced transport of phosphate and citrate	[387]
Diluted effluent from sodium dithionate processing, 35 g/L HCOONa, 30 g/L Na_2_S_2_O_3_…	ED with MVAs	Recovery of HCOONa 69%, with 87% purity, *η* = 70%, *E_spec_* = 96 kWh/m^3^	[388]
Steel manufacturing wastewater (Cl^−^, ${\mathrm{SO}}_{4}^{2-}$, Na^+^, Mg^2+^, Ca^2+^), 2.8–4.0 mS/cm, 36–72 mg/L COD	Sand filtration-EDR	Water recovery 75%, desalination 92%, concentrate COD below discharge limit, *E_spec_* = 0.85 kWh/m^3^, operation cost 0.146 $/m^3^	[389]
Secondary effluent from spinning processes, chemical industries, and metal processors (Cl^−^, ${\mathrm{SO}}_{4}^{2-}$$,\;{\mathrm{Mg}}^{2+},\;{\mathrm{Ca}}^{2+},\;{\mathrm{NO}}_{3}^{-}$$,\;{\mathrm{PO}}_{4}^{3-}$…), 7.3 mS/cm, 41.5 g/L COD	Sand filtration-EDR	Lower techno-economic efficiency compared to fiber filtration-UF-RO	[390]
ZnO washing wastewater (Na^+^, K^+^, Cl^−^, Ca^2+^ and ${\mathrm{SO}}_{4}^{2-}$), ~0.35 M, 1.2 mM TOC	ED with MVMs	Overall *η* ≈ 80%, divalent ions retained, thus scaling prevented, stable long-term performance of pilot plant with removal target of 50% (before evaporation)	[281]
Kraft pulp mill dissolved electrostatic precipitator dust (Cl^−^, ${\mathrm{CO}}_{3}^{2-}$$,\;{\mathrm{SO}}_{4}^{2-}$, Na^+^, K^+^), 137 g/L TDS (0.1 wt% TOC in the dust)	ED with MVMs	Selective removal of Cl^−^ at *η* = 60–78% and *E_spec_* ≈ 1 kWh/kg, organics in the dust recycled with the sulphate-rich diluate, no fouling, accumulated dust simply flushed, successful long-term operation, operation saving of 800 $/1000 ton Kraft pulp	[391]
Paper mill effluent, 6046 mg/L TDS, 390 mg/L COD	MF-ED	Max TDS removal ~90%, water recovery 80%, *E_spec_* ≈ 0.5 kWh/m^3^, concentrate usable as biomass	[392]
Primary textile effluent, 2,980 mg/L TDS, 220 mg/L COD	UF-ED	Desalination ~96%, *E_spec_* = 0.9 kWh/m^3^, reusable water	[393]
Model textile effluent with 1 g/L reactive blue 194 and 40 g/L Na_2_SO_4_	Tight UF-based diafiltration-BMED	Pre-concentration at a factor of 8 and diafiltration with 8 diavolumes, UF permeate with low dye content (2.7 mg/L) and 21.06 g/L Na_2_SO_4_, 99.5% dye recovery, ~99% salt conversion into 99% pure 0.29 M acid and 0.4 M base without fouling, *E_spec_ =* 4.2 kWh/kg	[394]
Model textile effluent with 0.25 g/L Remazol Brilliant Blue R and 50 g/L Na_2_SO_4_	BMED	Effect of zeta potential of dye molecule on fouling, fouling controlled by the identification of a “critical salt concentration” below which desalination cannot proceed due to fouling, *η* =39%, desalination 74%, 72% of Na^+^ and 66.9% of ${\mathrm{SO}}_{4}^{2-}$ converted into base and acid, respectively	[395]
Tannery unhairing effluent, pH = 12, 576 mg/L S^2−^, 23,289 mg/L COD, 436 mg/L Ca^2+^, 429.6 mg/L Cl^−^	ED with protective UF membrane on AEM	Anti-fouling solution against proteins and peptides, desalination 56%, 90% of organics retained within the diluate, thus water recycling	[396]
Almond processing treated wastewater (electrocoagulation and electrooxidation), 7.2 mS/cm, 296 mg/L TOC	ED	Concentration factor of 10 in the concentrate, diluate target 0.5 mS/cm, TOC removal ~70%, water recovery 94%, no fouling, scale-up at pre-industrial scale, *E_spec_* = 1.1–2.9 kWh/m^3^	[397]
Waste brine from olive pickling process, 103.3 mS/cm, pH = 3.5, 8033 mg/L dissolved organic carbon; coupled with storm water, 3.6 mS/cm	RED	*V_OC_* = 1.37 V (~70% of the ideal one, 10 cell pairs), *P_d,max_* = 0.59 W/m^2^ enhanced (with respect to NaCl solutions at the same conductivities, 0.44 W/m^2^) by pH gradient and organic acids (lower resistance)	[398]
Lysine fermentation effluent, 152 mS/cm, 17,800 g/L $\mathrm{NH}4_{4}^{+}$$,\;71,000\;\mathrm{mg}/L\;{\mathrm{SO}}_{4}^{2-}$, 102,300 ppm TOC	MF-ED	Separation of 73.1% ${\mathrm{NH}}_{4}^{+}$ and 83.5% ${\mathrm{SO}}_{4}^{2-}$, *E_spec_* = 106 kWh/m^3^, pulsed electric field effective against fouling, demineralized waste usable as animal feed, concentrate as fertilizer	[399]
Bio-refinery effluents: molasses effluents, lignocellulosic stream, sugar cane juice, 3.2–72.4 mS/cm, 38–380 g/L COD	ED	Salt removal 96% and 63% from lignocellulosic and molasses effluents, lower from sugar cane juice, low COD loss (< 6.3%), *η* = 69–104%, *E_spec_* = 0.44–1.59 kWh/kg salt	[400]
Bio-refinery effluents: synthetic salt mixtures with sorbitol, molasses effluent	ED	Simplified process model, predictions in good agreement with experimental results	[401]
Vinasse from a distillery producing ethanol from sugar can juice, 30,500 mg/L COD, 11.5 mS/cm	UF-ED with MVMs	K^+^ recovery 72%, *E_spec_ =* 9 kWh/m^3^, *η* = 54%, concentrated stream for fertigation, diluate stream for fertigation or biogas production (anaerobic digestion)	[402]
Solutions of 3-chloro-1,2-propanediol (α-monochlorohydrin or 3-MCH) (model effluent from biodiesel production or other sources), 10 or 30% wt + 0.1 M KCl	BMED	Recovery of glycidol by dehydrohalogenation caused by OH^−^ in the base compartment, selectivity 96%, *η* = 64%, glycidol distilled with 75.6% yield	[403]
Model antibiotic effluent with 0.95 g/L penicillin, 1 g/L ${\mathrm{SO}}_{4}^{2-}$ and 1 g/L bovine serum albumin	ED with UF membrane, 3-comp. (AEM-UF-CEM)	Penicillin recovery ~20%, removal of ${\mathrm{SO}}_{4}^{2-}$ from feed and antibiotic product 90%, no fouling, *E_spec_* = 0.058–0.082 kWh/g, estimated profit 6850 $/ton produced penicillin (8 L/day wastewater)	[404]
Effluent from anaerobic digester–decanter, 13,800 mg/L COD, 1700 mg/kg total N, 1800 mg/kg Cl^−^, 2,900 mg/kg Na^+^…	ED	Separation 70–96% for monovalent ions, < 50% for divalent ions, *E_spec_* = 6–11 kWh/m^3^ for water recovery 50–95%	[405]
Simulated supernatant of excess sludge mixed with influent from anaerobic-aerobic biological treatment, 100 mg/L P *	ED or ED-BMED	$\mathrm{ED}:\;{\mathrm{PO}}_{4}^{3-}$ recovery 95.8%, ED-BMED: 0.075 M H_3_PO_4_ recovered, *η* ≈ 70–80%, *E_spec_* = 5.3–29.3 kWh/kg	[406]
$\mathrm{Effluent}\;\mathrm{of}\;\mathrm{upflow}\;\mathrm{anaerobic}\;\mathrm{sludge}\;\mathrm{blanket}\;\mathrm{reactor}\;\mathrm{from}\;\mathrm{potato}\;\mathrm{processing},\;2.5\;\mathrm{mM}\;\mathrm{phosphate}\;(+K^{+},\;{\mathrm{NH}}_{4}^{+},\;{\mathrm{Cl}}^{-}\ldots )\;*$	SED-struvite precipitator	6.8 mM phosphate in SED product (from 0.8 mM initial product, struvite effluent), average overall *η* ≈ 70%, desalination 95%, phosphate recovery 93%, *E_spec_* = 16.7 kWh/kg phosphate	[407]

* These effluents did not contain organic matter; however, as they come from biological treatment, they could have, in general, residual organics.

**Table 4:** Recent experimental studies on energy recovery via RED from desalination brines.

High-Salinity Sol.	Low-Salinity Sol.	Performance	Ref.
SWRO brine 1 or 2 M NaCl	Secondary effluent 0.02 M NaCl	*V_OC_* (5 cell pairs), *P_d,max_*: 1 M–0.02 M → 0.90 V, ~0.48 W/m^2^ 2 M–0.02 M → 1.02 V, ~0.57 W/m^2^	[423] Figure 33a
• SWRO brine 1.2 M NaCl • FO brine 2.4 M NaCl	• River water 0.01 M NaCl • Seawater 0.6 M NaCl	*P_d,max_*, estimated maximum reduction of *E_spec_*: 1.2 M–0.01 M → 1.48 W/m^2^, 7.8%; 2.4 M–0.01 M → 1.86 W/m^2^, 13.5%; 1.2 M–0.6 M → 0.09 W/m^2^, 0.5%; 2.4 M–0.6 M → 0.37 W/m^2^, 2.2%	[540]
BWRO brine 31.3 mS/cm (~0.4 M), 19.5 mg/L dissolved organic carbon	Brackish groundwater 8.3 mS/cm (~0.095 M), 4.1 mg/L dissolved organic carbon	*V_OC_* = 0.53 V (10 cell pairs, 78% permselectivity), *P_d,max_* = 0.07 W/m^2^; with NaCl solutions at the same conductivity, *P_d,max_* = 0.09 W/m^2^ indicated effects of NOM and divalent ions	[398]
MD brine 4, 5 or 5.4 M NaCl (from 1 M feed, i.e., SWRO retentate)	Seawater 0.5 M NaCl	At 20 °C and 0.7 cm/s, *V_OC_* = 1.23–2.1 V (25 cell pairs), *P_d,max_* = 0.45–1.1 W/m^2^, water recovery 92%; at 10–50 °C, 5 M and 0.7 cm/s, *V_OC_* ≈ 1.7 and *P_d,max_* ≈ 0.5–1.05 W/m^2^; at 20 °C, 5 M and 1.1 cm/s, *P_d,max_* ≈ 1.1 W/m^2^ and *P_d,max,net_* ≈ 0.67 W/m^2^	[541] Figure 33b
• SWRO brine 1 M NaCl • MD brine 5 M NaCl	• Brackish water 0.1 M NaCl • Seawater 0.5 M NaCl	*V_OC_* (25 cell pairs), *P_d,max_*: 1 M–0.1 M → 2.1 V, 0.39 W/m^2^; 5 M–0.1 M → 3.4 V, 1.5 W/m^2^ (*P_d,max,net_* ≈ 1.2 W/m^2^, H_2_ production by alkaline polymer electrolyte water electrolysis cell 44 cm^3^/(h·cm^2^)); 1 M–0.5 M → 0.71 V, 0.05 W/m^2^; 5 M–0.5 M → 1.9 V, 0.55 W/m^2^	[542]
MD brine 2–5 M NaCl (from 1 M feed, i.e., SWRO retentate)	Seawater 0.5 M NaCl	At 20–60 °C, water recovery 75–95%, *V_OC_* = 1.26–1.95 V (25 cell pairs), *P_d,max_* ≈ 0.22–1.1 W/m^2^, exergetic efficiency 49% under best conditions, electrical energy consumption (1.3 kWh/m^3^) reduced by 23% and *E_spec_* (4.4 kWh/m^3^) reduced by 16.6% through RED inclusion	[536] Figure 33b
SWRO brine 1.1–1.5 M NaCl (RO with 30–50% water recovery from 43 g/L feed, i.e., high salinity seawater)	MCDI brine ~0.023 M NaCl (MCDI with 50–80% water recovery from ~0.85 g/L feed, i.e., SWRO permeate)	*P_d,max_* ≈ 2.45–2.83 W/m^2^, *E_spec_* = 2.0 kWh/m^3^ reduced by ~39% compared to RO-RO and by ~17% compared to RO-RO-RED	[537] Figure 33c
• ED diluate 38.1 mS/cm (from ED of real seawater in salt production plant) • Seawater 48.7 mS/cm	Distilled water 0.2 mS/cm (from evaporation of ED brine in salt production plant)	*V_OC_* (10 cell pairs), *P_d,max_*: 38.1–0.2 mS/cm → 2.02 V, ~0.23 W/m^2^; 48.7–0.2 mS/cm → 2.1 V, ~0.26 W/m^2^	[543]
ED brine (from ED of simulated seawater at 30 g/L sea crystal)	Simulated wastewater 0.8 g/L NaCl, 0.1 g/L KH_2_PO_4_, 0.1 g/L NH_4_Cl, 0.5 g/L glucose	Partial desalination of seawater (~60%) by consuming only the electrical energy produced by RED	[538] Figure 33d
Desalination brine 1.2 M NaCl	• Brackish water 0.02 M NaCl • Seawater 0.6 M NaCl	Partial desalination of brackish water (~75%) or seawater (~50%) by consuming only the self-produced electrical energy, the developed model predicted enhanced desalination by changing the operating conditions	[539] Figure 33e

## Abbreviations

AEL	Anion exchange layer
AEM	Anion exchange membrane
BM	Bipolar membrane
BMED	Bipolar membrane electrodialysis
BMSED	Bipolar membrane selectrodialysis
BWRO	Brackish water reverse osmosis
CEDI	Continuous electrodeionisation
CEL	Cation exchange layer
CEM	Cation exchange membrane
COP	Coefficient of performance
ED	Electrodialysis
EDI	Electrodeionisation
EDL	Electrical double layer
EDM	Electrodialysis metathesis
EDR	Electrodialysis reversal
EDTA	Ethylenediaminetetraacetic acid
FO	Forward osmosis
HPAM	Partially hydrolysed polyacrylamide
IEM	Ion exchange membrane
IX	Ion-exchange
IXR	Ion-exchange resin
MCDI	Membrane capacitive deionisation
MD	Membrane distillation
MF	Microfiltration
MVA	Monovalent selective anion exchange membrane
MVC	Monovalent selective cation exchange membrane
MVM	Monovalent selective ion exchange membrane
NF	Nanofiltration
NOM	Natural organic matter
RED	Reverse electrodialysis
REDI	Reverse electrodeionisation
RO	Reverse osmosis
SED	Selectrodialysis
SWRO	Seawater reverse osmosis
TDS	Total Dissolved Solids
UF	Ultrafiltration
VFA	Volatile fatty acid
WWRO	Wastewater reverse osmosis
WWTP	Wastewater treatment plant
ZLD	Zero liquid discharge
